# The N-terminus of alpha-1 giardin, a parasitic annexin orthologue, is essential for oligomerization and lipid-binding activity

**DOI:** 10.1371/journal.ppat.1014407

**Published:** 2026-10-14

**Authors:** Dawid Warmus, Corina D. Wirdnam, Erina A. Balmer, Tendai Muronzi, Manfred Heller, Özlem Tastan Bishop, Carmen Faso

**Affiliations:** 1 Institute of Cell Biology, University of Bern, Bern, Switzerland; 2 Graduate School for Cellular and Biomedical Sciences, University of Bern, Bern, Switzerland; 3 Research Unit in Bioinformatics (RUBi), Department of Biochemistry, Microbiology and Bioinformatics, Rhodes University, Makhanda, South Africa; 4 Proteomics & Mass Spectrometry Core Facility, University of Bern, Bern, Switzerland; 5 National Institute for Theoretical and Computational Studies (NITheCS), Stellenbosch University, Stellenbosch, South Africa; 6 Multidisciplinary Center for Infectious Diseases, University of Bern, Bern, Switzerland; 7 Institute for Infectious Diseases, University of Bern, Bern, Switzerland; Charles University, CZECHIA

## Abstract

Annexins are Ca² ⁺ - and membrane-binding proteins conserved across eukaryotes and involved in diverse membrane-associated processes, including unconventional protein secretion (UPS). In the intestinal parasite *Giardia lamblia*, annexins are represented by a family of 21 proteins termed alpha-giardins (αGs), all of which lack classical signal peptides yet are detected at the parasite surface and in the secretome. Here, we present a comprehensive functional analysis of αGs, with a particular focus on α1-giardin (α1G), a leading vaccine candidate against giardiasis and a putative virulence-associated UPS substrate. We show that αGs localize to peripheral endocytic compartments (PECs-PVs), specialized endo-lysosomal organelles implicated in protein release, and engage in multiprotein complexes enriched in UPS substrates. Using lipid-binding assays, cross-linking mass spectrometry (XL-MS), and structural modelling, we define conserved and divergent features of αG membrane interactions. Focusing on α1G, we combine site-directed mutagenesis, confocal microscopy, and mass photometry to demonstrate a critical role for its short N-terminal region in oligomerization and membrane association. Integration of these data with molecular dynamics simulations reveals how α1G oligomeric state and membrane occupancy could be regulated at PECs-PVs membranes. Taken together, our findings provide structural insights relevant to parasite virulence and vaccine development and contribute to a mechanistic model for annexin-mediated UPS in Giardia.

## Author summary

Annexins are proteins found in many organisms that help cells interact with membranes and transport materials without using standard secretory pathways. In the intestinal parasite *Giardia lamblia*, annexins exist as a family of 21 proteins called alpha-giardins (αGs), which are important for the parasite’s survival and infection. This study focuses on understanding the functions of αGs, especially alpha1-giardin (α1G), a promising vaccine candidate against giardiasis. We show that αGs associating with specialized endolysosomal compartments at the parasite-host interface can bind membrane lipids, unlike their cytosolic counterparts. Using laboratory experiments and computer simulations, we reveal that the short N-terminal region of α1G controls its ability to form oligomers and associate with membranes. These findings provide key mechanistic insights into how αGs interact with membranes in *Giardia* and support their role as targets for vaccine development against this widespread intestinal parasite.

## Introduction

Annexins are a protein family found in almost every eukaryote investigated to date, including animals, plants, and fungi [1–3]. The basic functions of annexins revolve around their interaction with membranes and calcium ions (Ca²⁺) [4–7] in the context of several cellular processes, including but not limited to membrane tethering [8], trafficking and organization of vesicles [9,10], ion transport [11–14], plasma membrane repair [15–18] and unconventional protein secretion (UPS) [19,20].

Annexins are characterized by a 70 amino acid sequence known as the annexin repeat (or fold) [1,4,21], each repeat presenting five alpha-helixes, four of which are parallel to each other and one perpendicular. Four to eight annexin repeats constitute the structurally conserved core region. Together, these form a curved shape with concave and convex sides (Fig 1). The convex side is responsible for interaction with Ca^2+^ and lipids [1,22] while the concave side, involving the N-terminal domain, takes part in protein complex formation [1,7]. In contrast to the core region, the N-terminal head region exhibits significant variability and a generally disordered structure.

**Fig 1:**
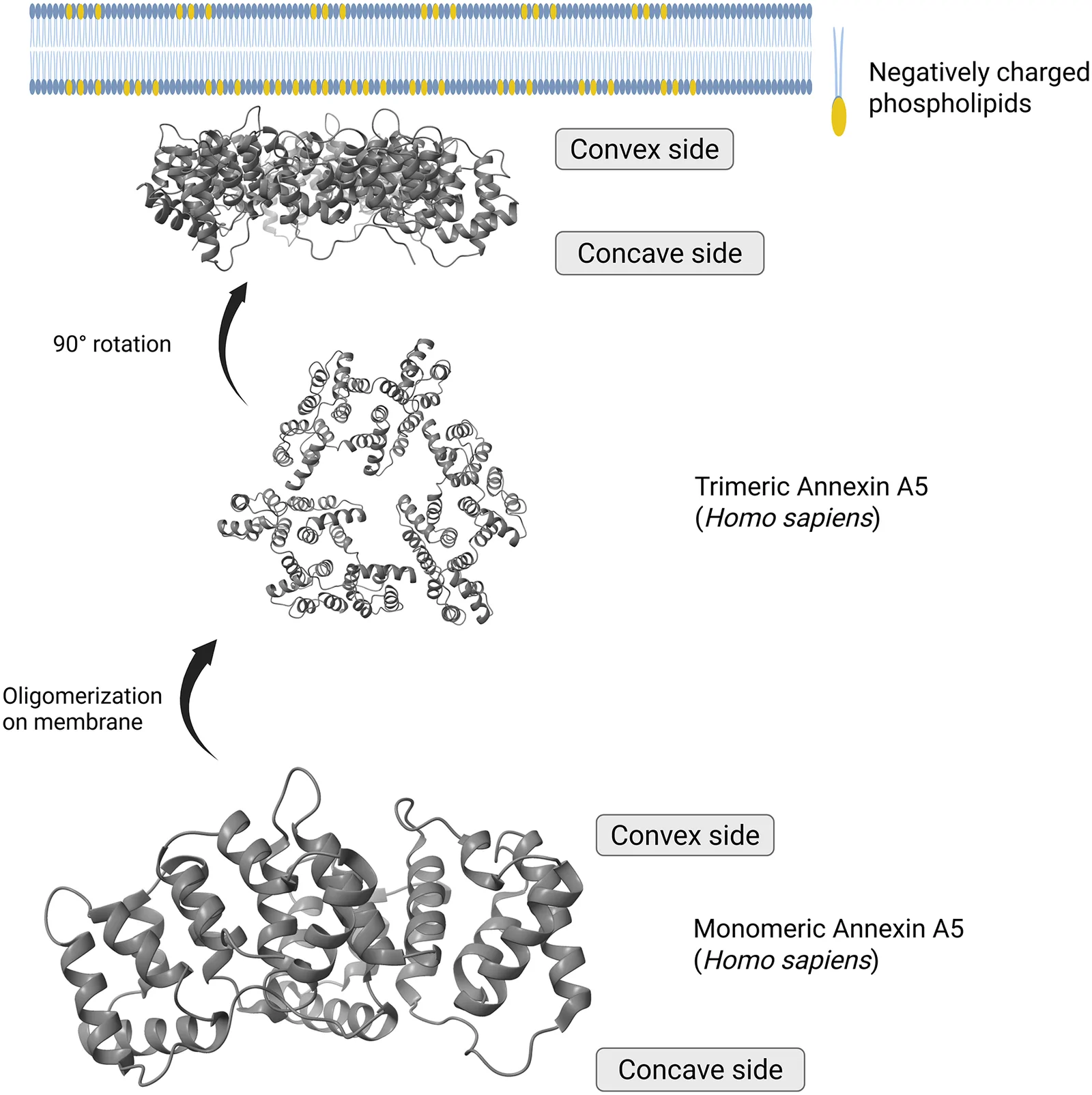
Human annexin A5 (anxA5) as a model for oligomerization and lipid binding at membranes. anxA5 is a well-characterized annexin, known to oligomerize prior to binding negatively charged lipids. As a general model for annexins, the convex side is responsible for contacting the membrane and is enriched for lipid binding residues.

At membranes, annexins can form various structures (Fig 1), ranging from simple aggregates to complex networks, depending on the specific annexin and membrane conditions [8,23–25]. The specific mechanism of membrane binding varies among different annexins and is influenced by factors such as membrane lipid composition, presence of Ca^2+^ ions and pH [1], [4,26,27]. Following membrane binding, annexins have been speculated to insert themselves into the target membrane [1,14,28]. This process is thought to involve a conformational change in annexin structure, leading to lipid bilayer destabilization.

Annexins from the intestinal protist parasite *Giardia lamblia* (syn. *intestinalis, duodenalis*) are called alpha-giardins (αGs) [1,2,29–36]. Two members of the 21-strong protein family, alpha 1-giardin and alpha 11-giardin (α1G and α11G), were previously identified as putative UPS substrates following their detection in the surface proteome [37] and secretome [38,39] of axenic *G. lamblia* cultures. Both proteins carry no *bona fide* signal sequence for secretion [37–39]. A recent investigation localized α1G and α11G to the parasite’s peripheral endocytic compartments [19] (PECs, also known as peripheral vacuoles, PVs), single membrane-bound endo-lysosomal organelles [40–43], previously implicated in protein release [19,44,45]. Furthermore, PECs-PVs-associated α1G and α11G participate in a protein complex which includes other UPS substrates, some of them recognized virulence factors in *G. lamblia* [19]. Importantly, α1G is currently considered one of the strongest candidates for vaccine development for giardiasis [34,37,46,47], a worldwide water-borne diarrheal disease widespread in areas where water sanitation is problematic and safe drinking water cannot be guaranteed.

In this report, we present a broad functional characterization of αGs in terms of their subcellular location in the *G. lamblia* system, their lipid-binding behaviour, and their protein interactomes and predicted configurations using XL-MS [48,49]. Given its importance as a vaccine candidate for giardiasis and its implication in virulence-related UPS mechanisms in *G. lamblia*, we narrow our focus to α1G. Using site-directed mutagenesis coupled to confocal microscopy and mass photometry (MP), we dissect the contribution of the short (less than five residues) N-terminal region of α1G and investigate oligomeric forms of both wild type (WT) and mutant α1G variants in solution. These data are then used to inform simulations of molecular dynamics of α1G variants, to predict the behaviour of α1G variants at PECs-PVs membranes in terms of oligomeric stability and occupancy, and to develop a new working model for α1G function at PECs-PVs membranes.

## Results

### A subset of αGs localizes in close proximity to peripheral endocytic compartments

To determine where every αG accumulates within the *G. lamblia* trophozoite, and especially to discover additional PECs-PVs-associated ones which might also be implicated in virulence-associated UPS, we generated constructs for the epitope-tagged expression of every member of the αG family and transfected them to generate episomal transgenic Giardia lines (S1 Fig). Aside from α1G and α11G, previously shown to accumulate at PECs-PVs [19], five more αGs (α5G, α6G, α12G, α15G and α17G) were also found in close proximity to these organelles (Fig 2). Several αGs presented a diffused and generally cytosolic pattern of accumulation, while others appeared distinctly at flagella (Fig 2). For three giardins (α7.3G, α14G, α18G) the expression was too low for any conclusive visualization. Giardins α7.1G and α7.2G differ in only eight amino acids, therefore we focused only on one of them, α7.2G.

**Fig 2:**
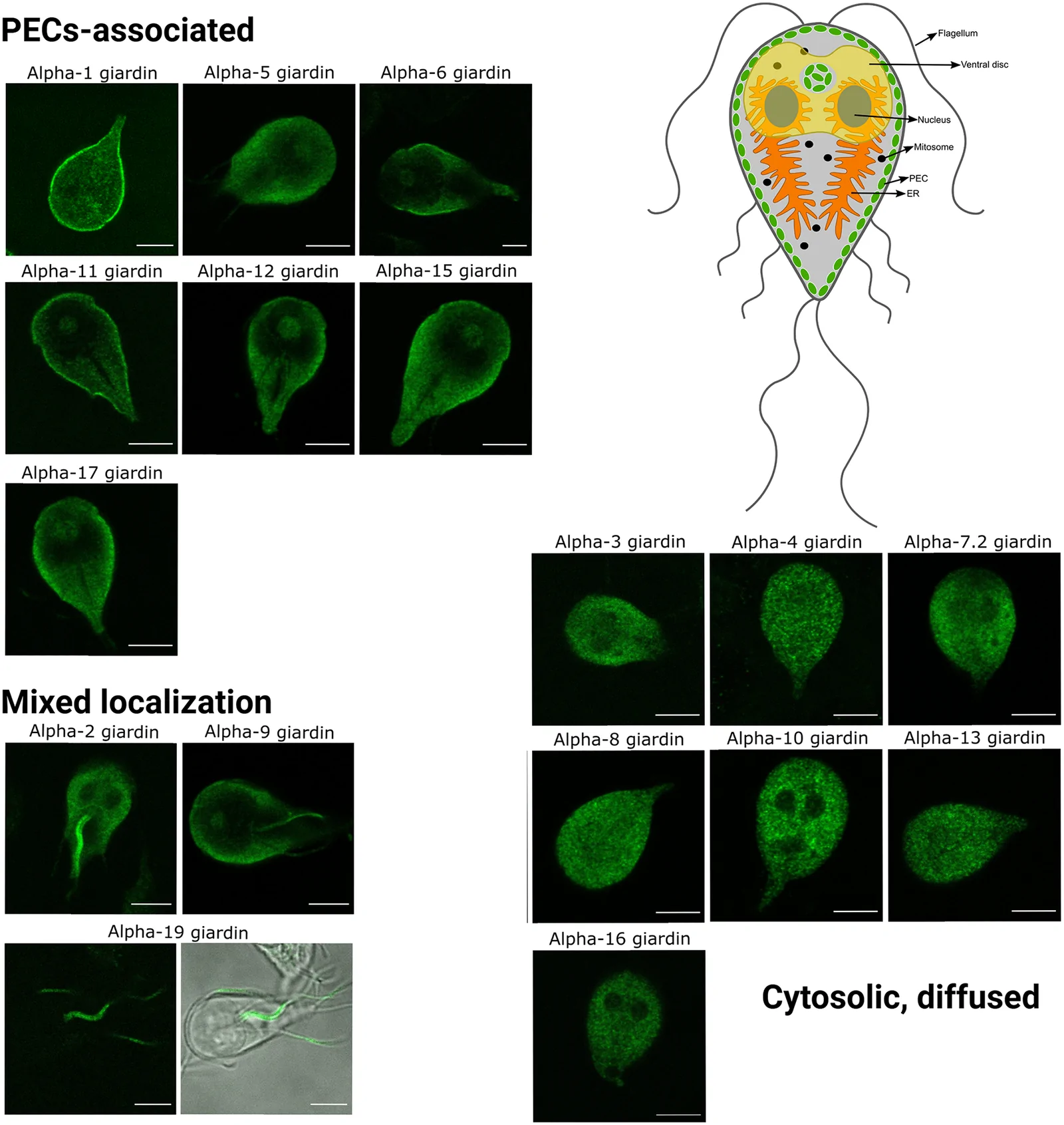
Confocal fluorescent light microscopy investigation of subcellular localization of epitope-tagged αGs. Images are grouped based on subcellular localization profiles. A scheme of a *G. lamblia* trophozoite, highlighting known compartments and PECs-PVs (green). Scale bar: 5um.

### Lipid binding activity of G. lamblia-extracted PECs-PVs-associated αG complexes and native co-IP analysis

To determine whether differentially localized αGs present distinct lipid-binding behaviour and based on confocal microscopy data (Fig 2), we selected four transgenic Giardia lines expressing PECs-PVs-associated αGs (αG1, 2, 6 and 11), and five expressing cytosolic-diffused αGs (αG7.2, 8, 9, 13, 16 and 19). Lines were chosen based on comparable levels of epitope-tagged variant expression, thereby minimizing the impact of overall protein production on testing outcomes for lipid-binding capacity/specificity.

Protein antibody-mediated pull-down using HA-tagged αGs as affinity handles was followed by incubation of membrane lipid strips with the IP-ed eluate fraction, with no calcium ion addition. Antibody-based detection of the HA tag showed that, in contrast to αG complexes extracted from lines expressing epitope-tagged αGs 7.2, 9, 13, 16 and 19, where no appreciable signal was recorded, lipid residue binding was detected for protein complexes containing PEC-PV-associated αGs, including affinity for phosphatidyl-inositol phosphate (PIP) residues (Fig 3A). α1G affinity for PIP residues was further analyzed using a PIP array (Fig 3B), highlighting PI(3)P, PI(5)P and PI(3,5)P as preferred targets for binding, in these experimental conditions.

**Fig 3:**
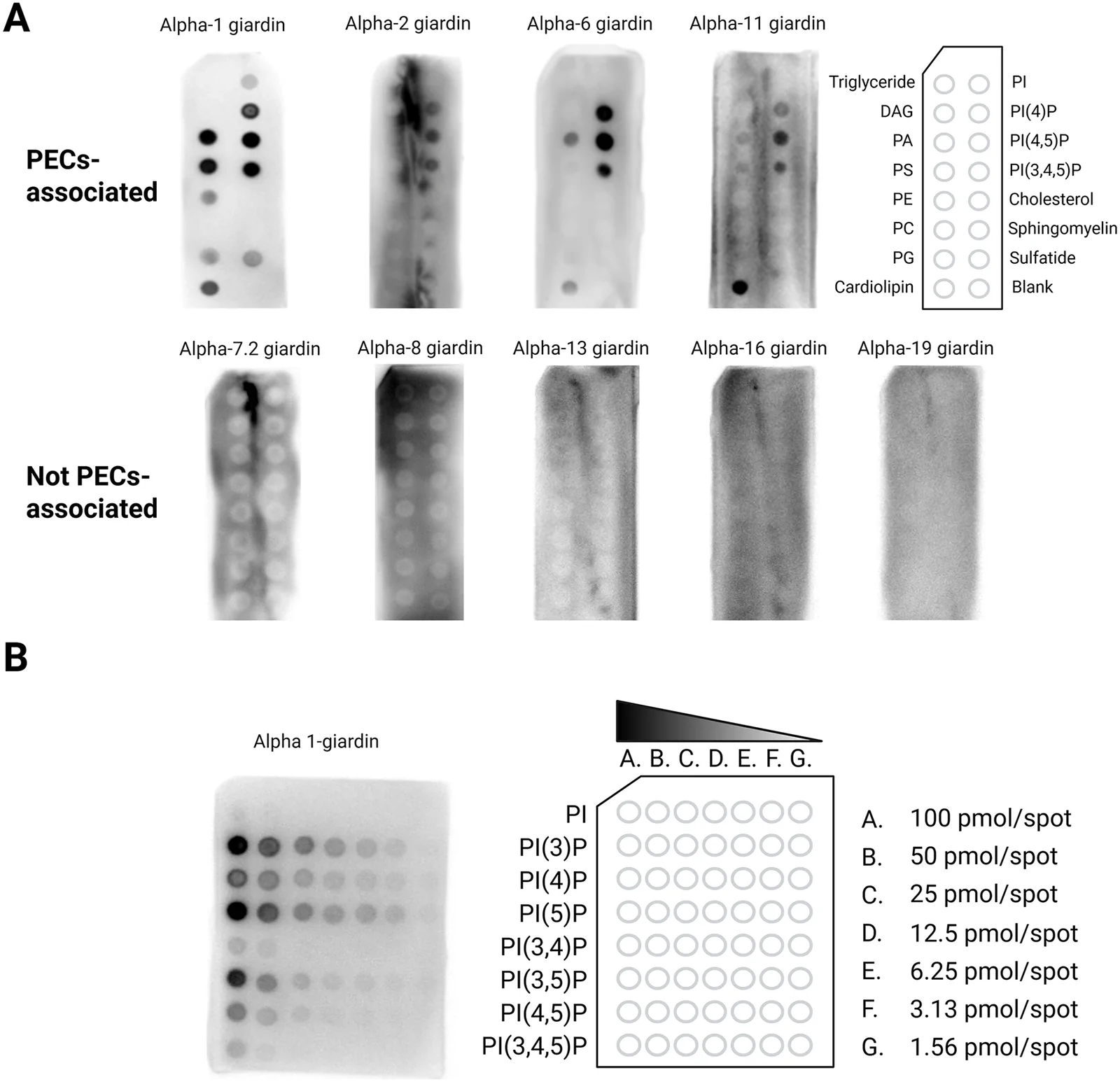
Lipid-binding activity of IP-ed epitope-tagged αGs. A. αGs associated to PECs-PVs show a distinct binding profile using lipid strips whereas lipid binding activity for cytosolic/diffused αGs is not detected in the same experimental conditions. **B.** A finer analysis of lipid binding activity for α1G, with a focus on various PIP residues, using a PIP array.

To further investigate the composition of -PEC-PV-associated αG complexes, especially in comparison to α8G complexes which are never detected at PECs-PVs and show no appreciable lipid binding activity, biological replicates of IP-ed protein complexes from Giardia lines expressing HA-tagged α1G (line G25), α6G (line G183), α11G (line G190), α8G (line G196), were analysed by tandem MS and compared to control fractions prepared from non-transgenic WB cells. A qualitative and comparative analysis using %riBAQ values across all samples (Fig 4A; S1 Data), followed by a ranking of the top ten identified proteins in each sample, shows a clear enrichment for each affinity handle compared to the WB control (Figs 4B-E; S1 Data). Core interactomes for α1G (Fig 4B), α6G (Fig 4C) and α11G-containing complexes (Fig 4E) are enriched for proteins previously shown to reside at PECs-PVs, including NEK kinases and UPS substrates with suspected roles in virulence (*e.g.,* Xaa-dipeptidase/prolidase) [19]. In contrast, the core interactome of α8G-containing complexes is consistent with its mainly cytosolic deposition and non-detectable lipid binding activity (Fig 4D).

**Fig 4:**
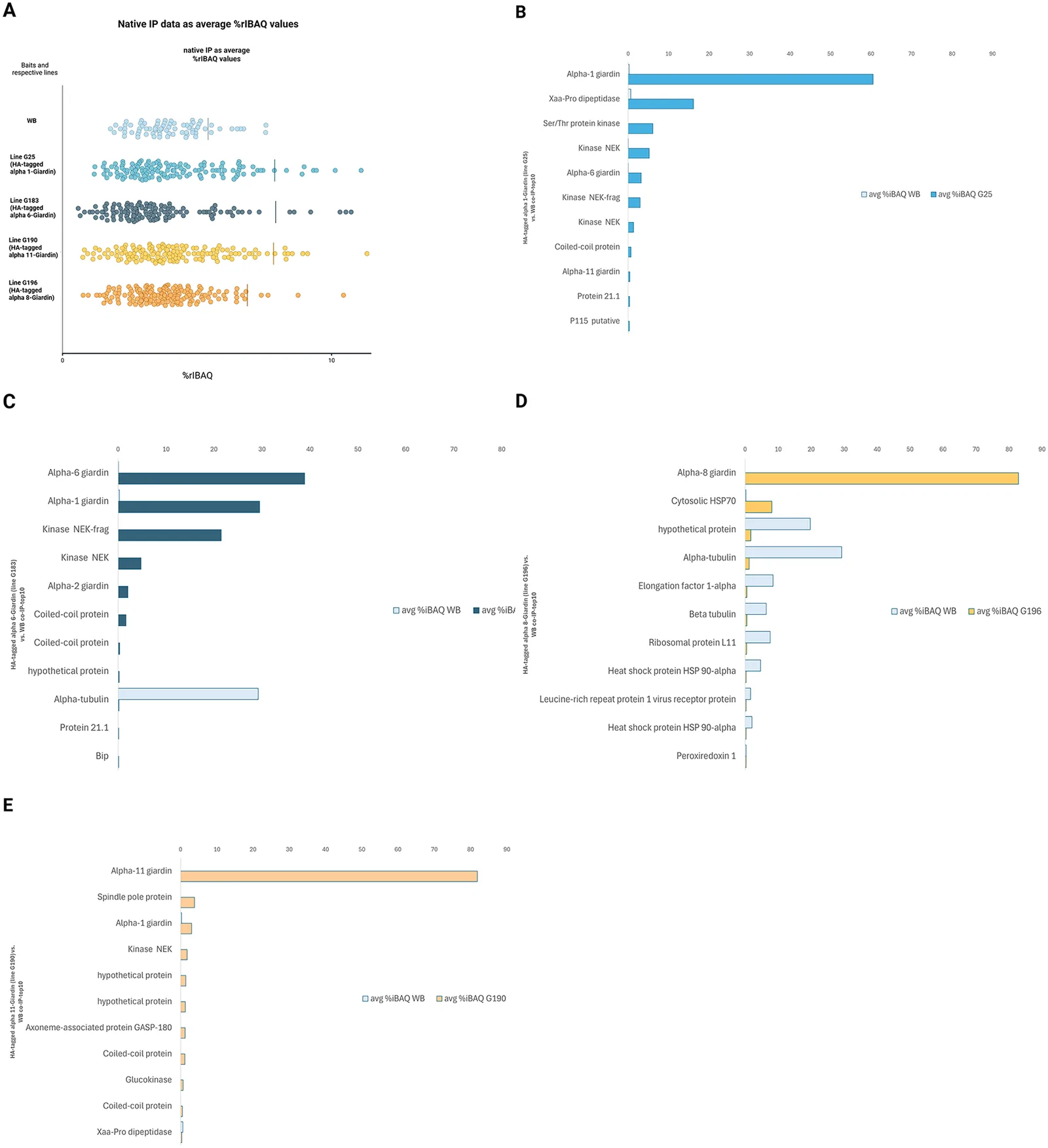
Native co-IP data analysis expressed as %rIBAQ values for HA-tagged affinity handles α1G, α6G, α8G, and α11G. **A.** Base-10 distribution of native co-IP data for each affinity handle, compared to native co-IP data for a non-transgenic WB control sample. The bar indicates the median, representing the 50th percentile where half the data points are lower and half are higher. Top identified protein hits based on %rIBAQ values for each protein pull-down using **B.** α1G, **C.** α6G, **D.** α8G and **E.** α11G as affinity handles.

### XL-MS detection of inter- and intra- links in α1G complexes informs on structural configurations and possible sites of protein-protein interaction

To measure and calculate possible configurations of α1G-containing complexes, IP-ed protein complexes from cells expressing HA-tagged α1G (line G25), α6G (line G183), α8G (line G196) and α11G (line G190), were further analyzed by XL-MS using DSSO treatment followed by detection of covalently-bound peptides (Fig 5), either from within the same protein (intra-links) or between distinct proteins (inter-links), within the isolated complex [48] (S2 Data).

**Fig 5:**
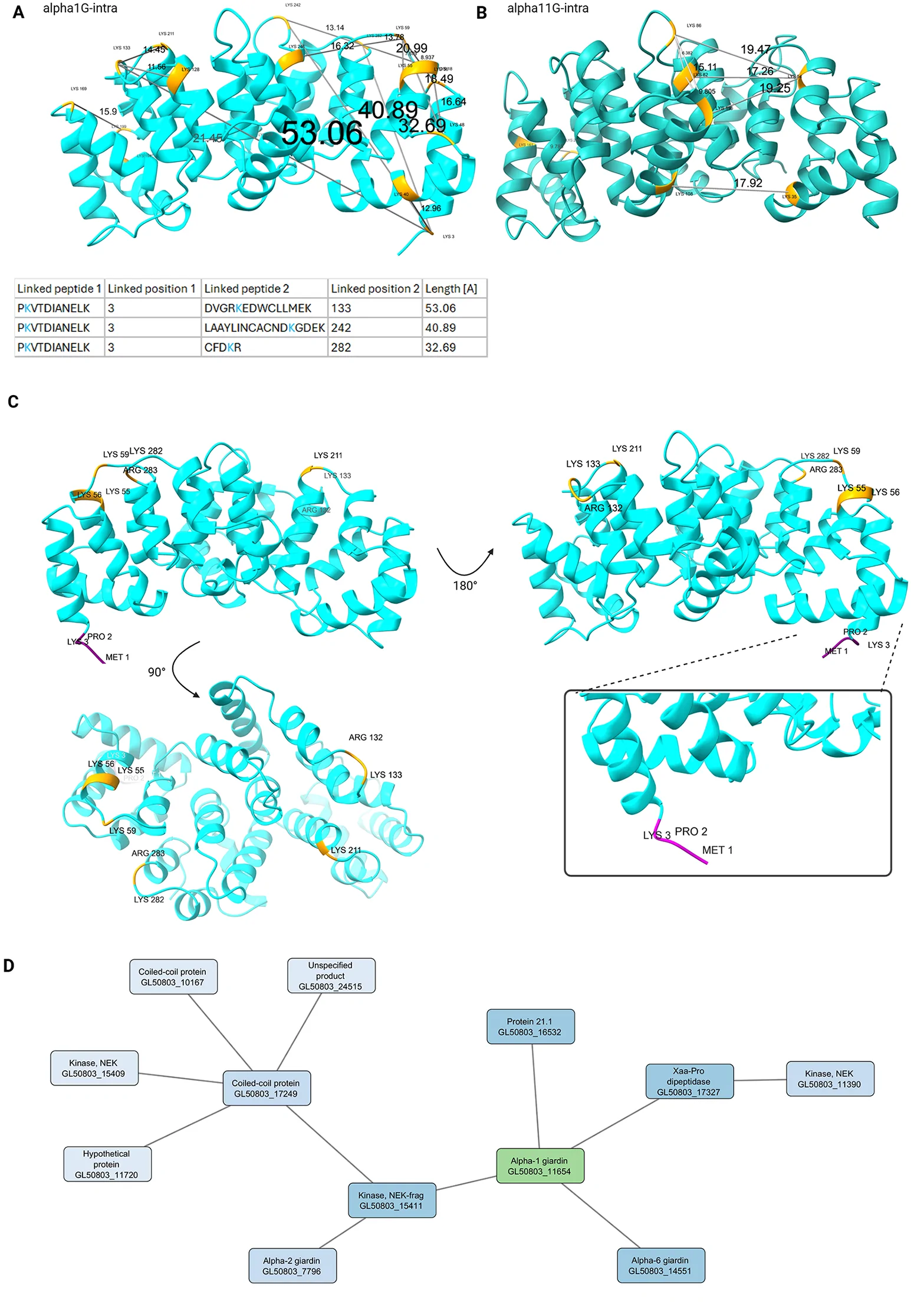
Structural modelling of inter- and intra- links, following analysis of XL-MS data for α1G and α11G. **A.** XL-MS data analysis highlights the presence of intra-links compatible with oligomerization of α1G, mediated by the N-terminal region and reported in detail in the table. **B.** XL-MS data analysis for α11G does not detect the presence of intra-links exceeding the threshold for interface crosslinking. **C.** Mapping of selected residues and structural modelling analysis of α1G highlights the short N-terminal region (fuschia) and putative lipid binding residues (orange). **D.** Visualization of tiered XL-MS data with a focus on α1G (green). First, second and third tier physical interactions are represented by gradually lighter shades of blue, respectively.

The detection of *intra*-links between covalently bound peptides from within α1G presents a discrepancy in terms of molecular interaction distances. The presence of intra-links significantly longer than the 25Å threshold (Fig 5A) can only be explained as derived from α1G homo-oligomers. In contrast, intra-links derived from within α11G (Fig 5B), α6G and α8G, never exceed the upper limit of 25Å, indicating how these αGs are unlikely to form homo-oligomers (S2 Data). The *inter*-links data (Fig 5D; S2 Data) point to a direct physical connection between α1G, a NEK kinase (ORF GL50803_15411), α6G and Xaa-dipeptidase. Surprisingly, although robustly present in all co-IP datasets previously reported [19], and also presented here, no *inter*molecular link could be detected between either α2G or α11G, to α1G.

To identify interaction sites within the predicted structure of α1G which could be important for its function and structural configuration, we mapped the exact residues deduced from both inter- and intra- links data for α1G. This analysis led to the realization that the N-terminus of this protein is a hub for interactions. In addition, based on the predicted structure of α1G, several positively charged residues on the convex side were identified as potentially important for lipid binding activity (Fig 5C).

### XL-MS informed targeted mutagenesis and subcellular localization of alpha1-giardin mutants and variants

To investigate the N-terminus of α1G and specific net positive charge residues for a possible role in protein localization, functionality and lipid-binding activity, as well as testing the predictive power of *in silico* structural analysis of the XL-MS data, we generated several constructs for expression of α1G mutant and deletion variants in transgenic *G. lamblia* lines (Fig 6). *G. lamblia* lines G25 and G282 (Fig 6A, tagged proteins) have hitherto been used to express WT HA-tagged α1G, and serve as reference-control lines. The corresponding constructs differ by only three nucleotides upstream of the start codon, with line G282 being the direct control for line G283, and both constructs give rise to the same protein product.

**Fig 6:**
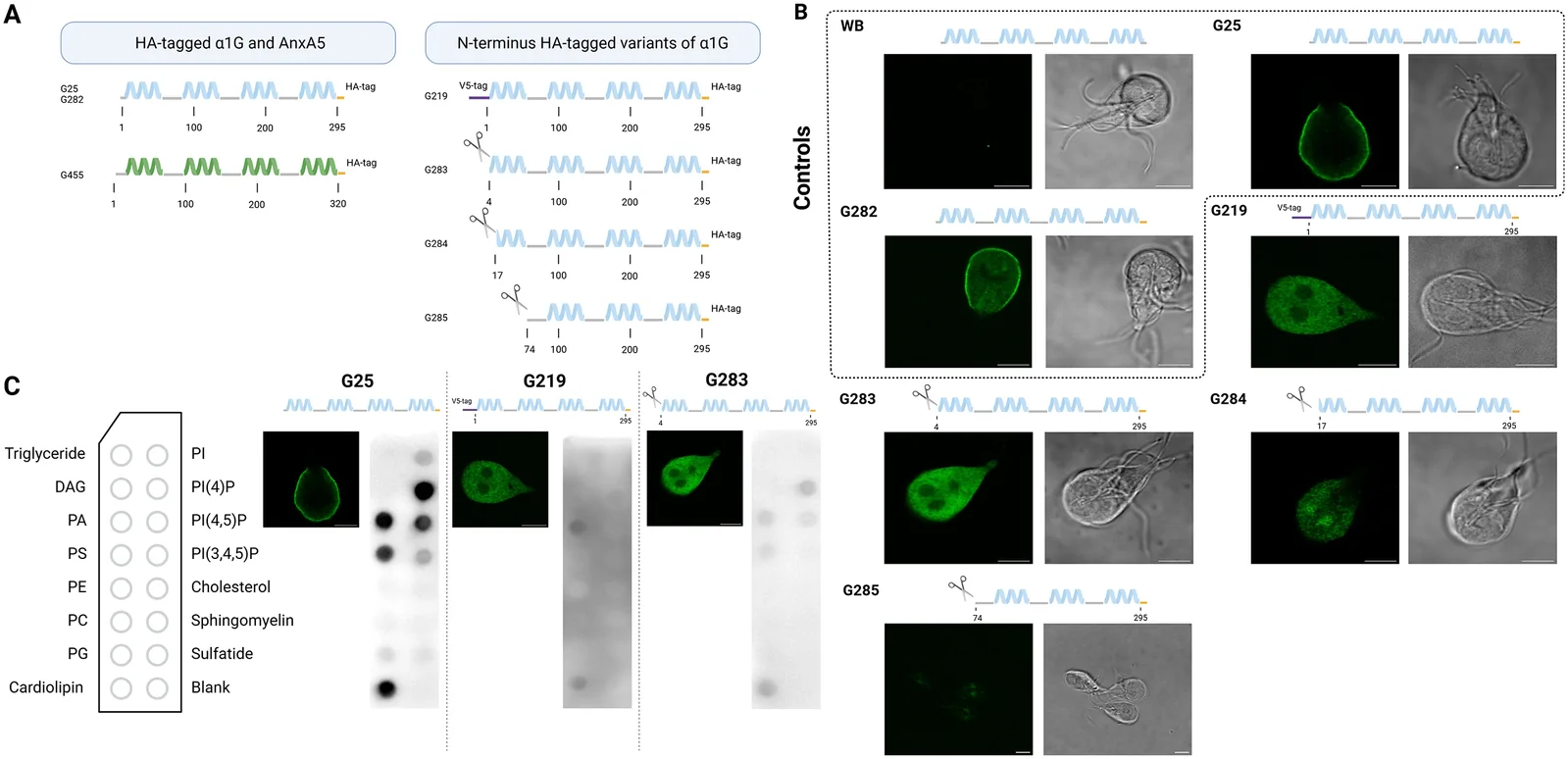
Site-directed mutagenesis and truncation analysis of α1G, compared to α8G and anxA5. **A.** *G. lamblia* lines G25 and G282 were developed to overexpress HA-tagged α1G, whereas line G455 was engineered to express codon-optimized human anxA5, similarly HA-tagged at the C terminus. Lines G219, 283, 284 and 285 were engineered to express α1G N-terminus variants, either by insertion of a V5 epitope tag or by increasingly longer truncations of the N-terminal region. **B.** Confocal microscopy analysis following IFA of lines listed in **A.** Reference control samples including a non-transgenic WB control, and lines G25 and G282, are boxed **C.** Lipid strip binding data for HA-tagged protein complexes extracted from lines G25, G219 and G283, including confocal microscopy analysis of IFA samples for each line. Scale bar: 5um. Created in BioRender. Faso, **C.** (https://BioRender.com/cw1s7xm) and licensed under CC BY 4.0.

Giardia lines G220, 222, 232, 233 and 248 were engineered to express different combinations of Lys and Arg mutants, which were posited to play a role in lipid binding (Figs 5C and S2). On the other hand, lines G219, 283, 284 and 285 (Fig 6A, N-terminus mutants) were developed to interrogate the functional role of the short N-terminal region of α1G. The outcome of the microscopy-based analysis of the expression of all listed constructs in the respective lines shows that, of all the α1G variants tested, those with a modified N terminus, either by addition of a V5 epitope tag (line G219) or by deletion of progressively larger N-terminal segments, lost targeting to PECs-PVs (Fig 6B). PECs-PVs localization is already lost upon deletion of the first three amino acid residues (line G283). In line with these data, when HA-mediated IP eluates from lines G219 and G283 were tested on lipid strips alongside the reference eluate from line G25, no appreciable lipid-binding activity could be detected (Fig 6C). All tested mutants for possible involvement in lipid binding were detected at PECs-PVs (S2 Fig).

To test each α1G variant in isolation as opposed to testing the behaviour of α1G-containing protein complexes extracted by co-IP from *G. lamblia* cell extracts, we attempted heterologous expression of α1G in both *E. coli* and in *S. cerevisiae*. Although protein expression was successful overall, both expression systems failed to provide functional protein (based on testing of lipid binding capacity on lipid strips; S3 Fig).

To interrogate αG behaviour in living cells, we engineered and transfected constructs for expression of: a) full-length α1G fused to mNeonGreen (mNG) at the C terminus (line G370), b) an mNG C-terminally tagged version of the α1G variant with a deletion of the first three amino acid residues (Δ3α1G; line G390), and c) an mNG C-terminally tagged α8G variant (line G391). A line engineered to express free mNG (line G369) was included as a control. Live cell imaging data (S1–S4 Videos) show how, in contrast to the mNG fusion to full-length α1G which associates to PECs-PVs, and in line with previous data, the -3 N-terminal deletion construct Δ3α1G fused to mNG (line G390) loses PECs-PVs targeting and mimics more closely the deposition of both a free mNG (line G369) and the mNG fusion to α8G (line G391).

### An intact N-terminus is required for oligomerization of alpha1-giardin

Mass photometry (MP) was employed to assess the oligomeric state of protein samples under native conditions. The analysis included a control sample (WB) and three test proteins: α1G (line G282), α8G (line G196), and Δ3α1G (line G283), all HA-tagged (Fig 7A and S3 Data). The WB sample showed a single, narrow peak (Fig 7A) corresponding to the background signal typically observed in MP experiments (40–70 kDa).

**Fig 7:**
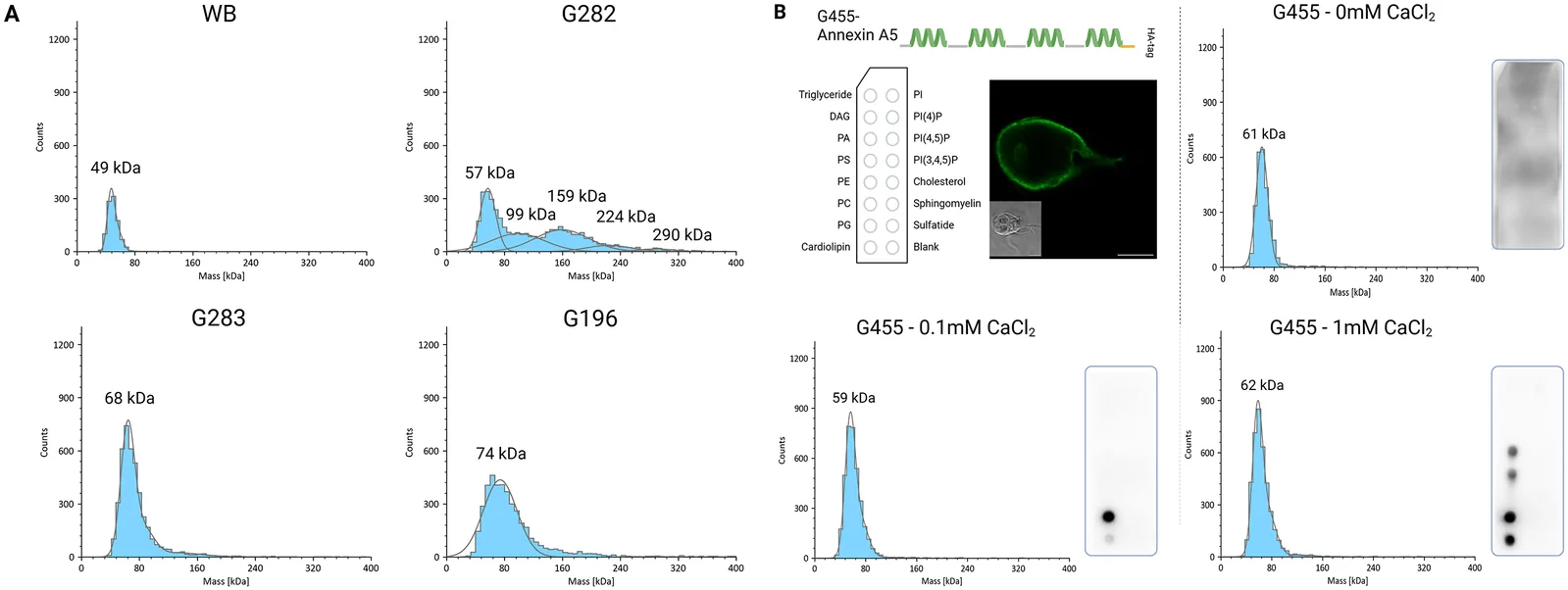
Mass photometry analysis for HA-tagged α1G, Δ3α1G, α8G and anxA5, extracted from lines G282, G283, G196 and G455, respectively. **A.** Mass distribution (in kDa) of extracted HA-tagged α1G (line G282), Δ3α1G (line G283) and α8G (line G196) containing complexes (in duplicate) in PBS, compared to a similarly prepared sample from non-transgenic WB cells as control (WB). **B.** Mass distribution (in kDa) and lipid strip binding activity of *G. lamblia*-expressed anxA5 extracted from line G455 and in HEPES, with increasing amounts of CaCl_2_. Note that no lipids were added to this solution prior to either MP or lipid strip assays.

Interestingly, α1G, with a predicted monomeric mass of 35 kDa, showed a distinct pattern of multiple mass fractions (Fig 7A; line G282). In addition to the background peak, several higher-mass species were observed at approximately 99 kDa, 159 kDa, 224 kDa, and 290 kDa. These values suggest the presence of oligomeric states, potentially corresponding to trimeric, pentameric, and higher-order assemblies. Although the higher-mass species may reflect complexes with α1G-binding partners, co-IP data indicate that α1G is the main component of extracted complexes, with other proteins present only in trace amounts.

Along with α1G, we tested α8G (line G196) as a representative cytosolic giardin, and -Δ3α1G (line G283). Both extracted proteins displayed only a single peak centered around 70 kDa (Fig 7A). In these experimental conditions, no other higher-order mass species could be detected in either sample, suggesting both proteins may also exist in a dimeric form. Interactome analysis of protein pull-down experiments of HA-tagged Δ3α1G extracted from line G283, with respect to the same analysis performed using the control line G282, registered a marked increase in %rIBAQ of the target bait and the absence of Xaa-pro dipeptidase in line G283 (S4 Data). Furthermore, XL-MS analysis of HA-tagged Δ3α1G complexes did not detect inter-links specific to WT α1G (S5 Data). At the same time, all links involving the missing N-terminus were absent (Fig 8A and 8B), while a novel 40Å link (Fig 8C) was detected. This, following *in silico* reconstruction, is consistent with a dimeric form for the mutant.

**Fig 8:**
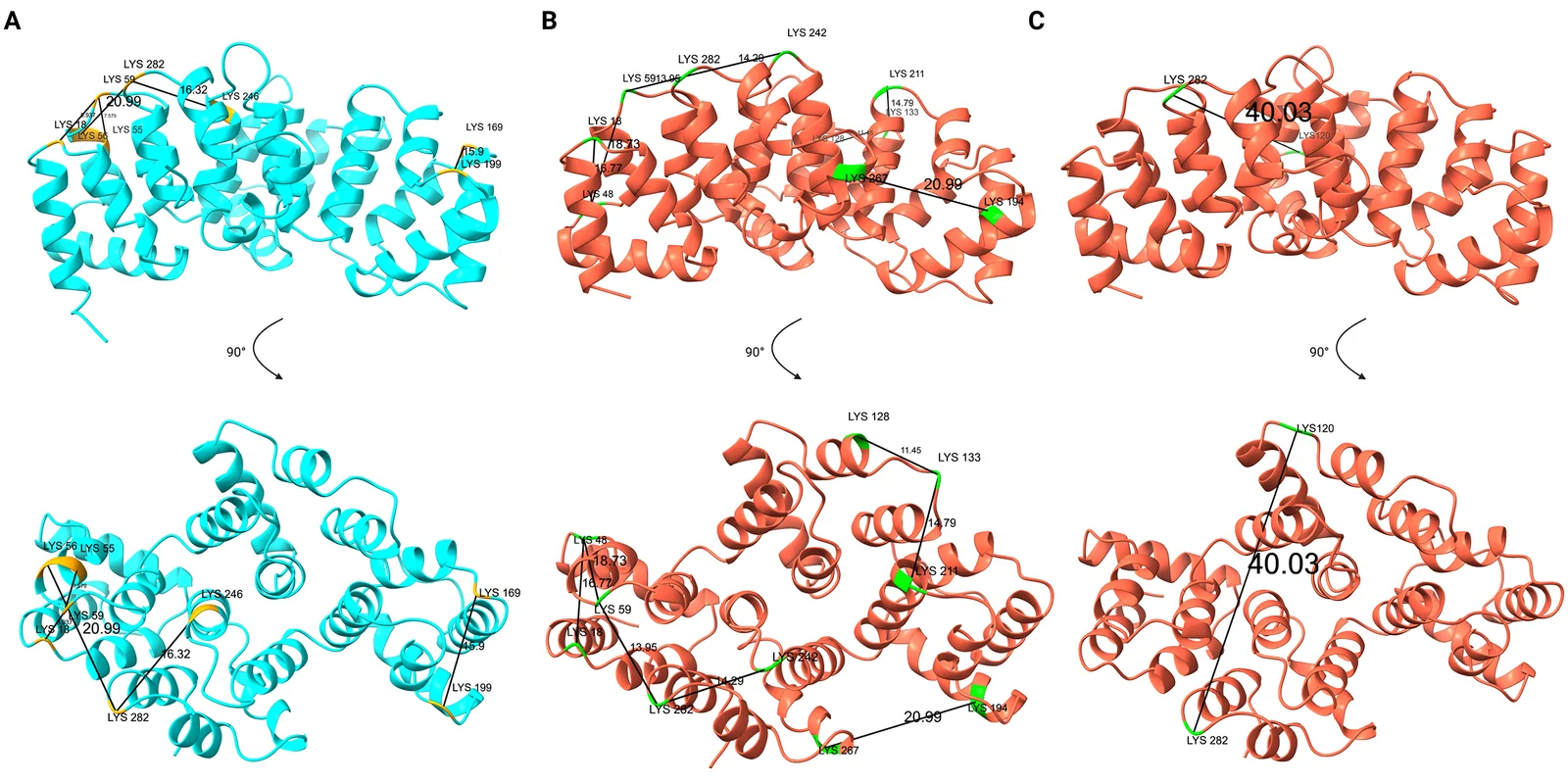
Intra-crosslinks identified by XL-MS for Δ3α1G (orange) and α1G (cyan), visualized frontally (convex-concave) and at 90° rotation. **A.** Intra-links present exclusively in α1G, with the omission of N-terminal intra-links for clarity. **B.** Intra-links common to both Δ3α1G and α1G, presented on the Δ3α1G structural model. **C.** A unique crosslink detected for Δ3α1G, presented on the Δ3α1G molecule model, is *not* detected in α1G.

### G. lamblia-expressed human annexin A5 localizes to PECs-PVs and binds negatively charged lipids

Unlike parasitic annexins, which are under-investigated, mammalian annexins have been reported on extensively. One of the better functionally and structurally characterized annexins, which was also developed as a molecular detection tool for pre-apoptotic cells, is human annexin A5 (anxA5) [8,13,18,23,50–58]. AnxA5 is known to require calcium ions and oligomerization for lipid binding. We wondered whether anxA5 expressed in *G. lamblia* would behave similarly to α1G in terms of PECs-PVs targeting and lipid binding properties. To address this question, we engineered line G455 to express HA-tagged *G. lamblia* codon-optimized anxA5. Confocal microscopy analysis following IFA showed deposition of the HA-tagged reporter at the periphery of the cell, clearly proximal to PECs-PVs (Fig 7B). *G. lamblia*-expressed anxA5 was further extracted for testing in lipid strip assays at different calcium concentrations. In the absence of calcium, there was no detectable lipid binding (Fig 7B). As calcium levels increased, we detected binding to Cardiolipin and Phosphatidylglycerol at 0.1 mM Ca² ⁺ , and additional binding to Phosphatidic acid and Phosphatidylserine at 1 mM Ca² ⁺ . The same anxA5 protein sample was analysed by MP (Fig 7B and S3 Data). No oligomer formation could be detected in the MP assay under any condition, consistent with reports showing how trimerization of human anxA5 is a lipid-dependent process [18,59].

### Molecular dynamics of oligomeric alpha1-giardin variants and membrane binding simulations

To investigate the membrane-binding behaviour of the potentially native α1G trimer, molecular dynamics (MD) simulations were performed across five distinct lipid compositions (S6 Table) with the α1G trimer deprived of its first methionine residue; thus, all residue numbers shown here are lower by 1 compared to previous representations. The base membrane contained phosphatidylcholine (PC), phosphatidylserine (PS), and cholesterol, while the other systems included either 3% or 12% of PI(3)P or PI(5)P (Supplementary videos 5–24). In all cases, the protein interacted with the membrane, but the nature of the interaction varied depending on PIP presence and type. In the case of the basic membrane, the interaction was short-lived, and the trimer dissociated quickly. However, after the addition of PIPs, the interaction between the trimer and membrane was stabilized.

To better understand which regions of α1G contribute to lipid binding, protein-lipid interaction parameters were analysed across all simulations (S6 Data). Based on the duration, occupancy, and K_*off*_, the residues most significantly interacting with PIPs were identified and grouped in two categories (Fig 9).

**Fig 9:**
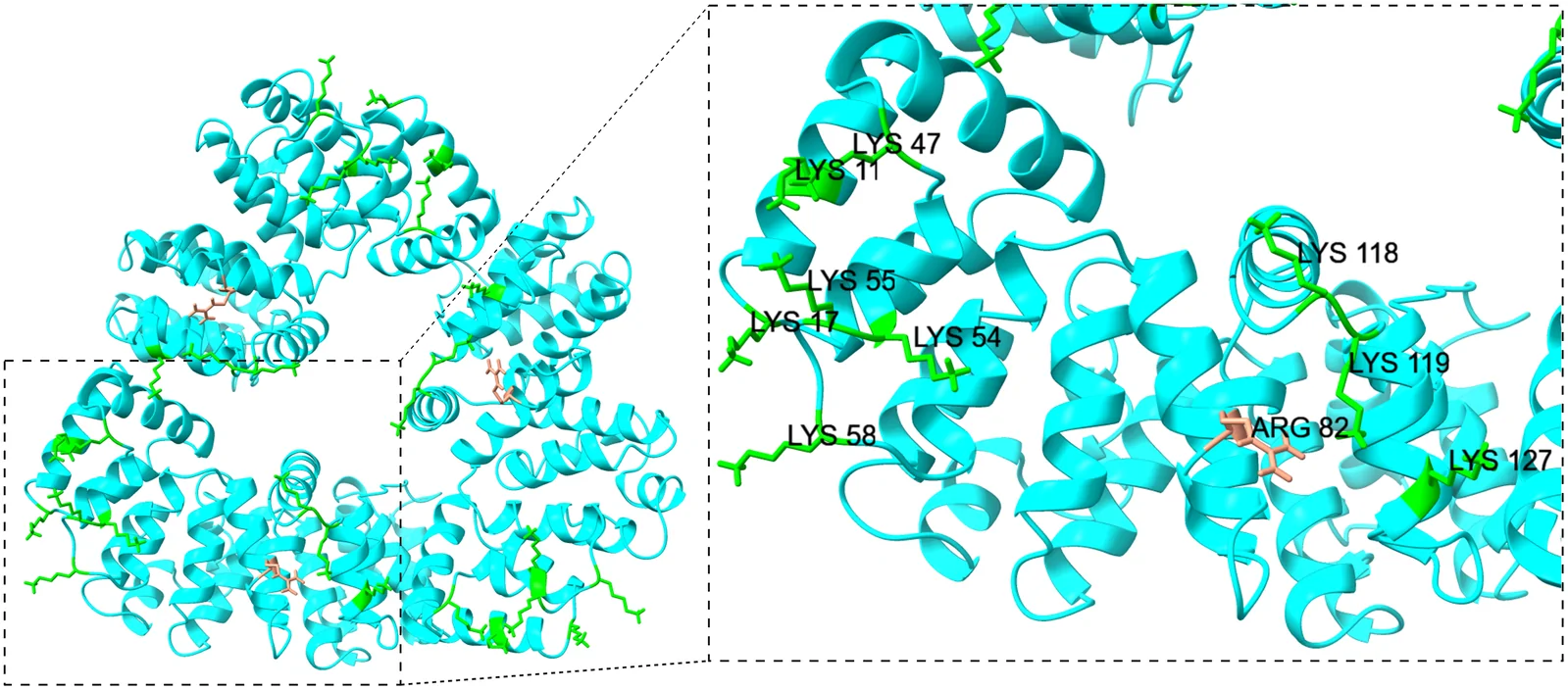
Potential lipid interacting residues of α1G based on molecular dynamics simulations. Amino acid residues involved in stable, long-lasting interactions with duration and residence times above 50 ns, and low dissociation rates (Koff < 10 µs ⁻ ¹) are shown in dark tuna. Residues involved in frequent but short-lived contacts (< 20 ns) with occupancy values above 0.5 and K_*off*_ values below 50 µs ⁻ ¹ are shown in green. For data pertaining to each residue, refer to S6 Data.

The first group contains residues that form stable, long-lived contacts - defined as interactions with duration and residence times above 50 ns, and low dissociation rates (K_*off*_ < 10 µs ⁻ ¹). Only Arg82 in the 12% PI(3)P membrane met all these criteria. In contrast, Arg82 in the PI(5)P membrane fell short of the threshold due to a slightly lower interaction duration (~35 ns). Notably, the occupancy of Arg82 is moderate, which is consistent with rarer but more stable interactions.

The second group consists of residues that interacted more transiently but frequently. These residues exhibited occupancy values above 0.5, shorter contact durations (< 20 ns) and K_*off*_ values below 50 µs ⁻ ¹. For simulation of interaction with the 3% PI(3)P membrane, only Lys55 met these criteria, whilst six residues - Lys17, Lys55, Lys58, Lys118, Lys119 and Lys127 were identified for the same simulation using 12% PI(3)P-enriched membrane. In case of the 3% PI(5)P membrane, only Lys47 and Lys55 met the outlined criteria. At 12% PI(5)P-enrichment, five residues - Lys11, Lys17, Lys54, Lys55 and Lys58 are singled out, based on the above-mentioned criteria, as the most likely sites of membrane interaction (Fig 9).

## Discussion

The data presented in this report point to a fundamental role for the N-terminus of α1G in oligomerization, lipid-binding ability and correct subcellular targeting to PECs-PVs. To synthesize all the data presented so far, while taking stock of previous reports [2,19,35–37,39], we propose a new working model for αGs at PECs-PVs, with a focus on α1G as an immunodominant and highly promising giardiasis vaccine candidate [34], implicated in UPS mechanisms at the parasite-host interface [19] (Fig 10).

**Fig 10:**
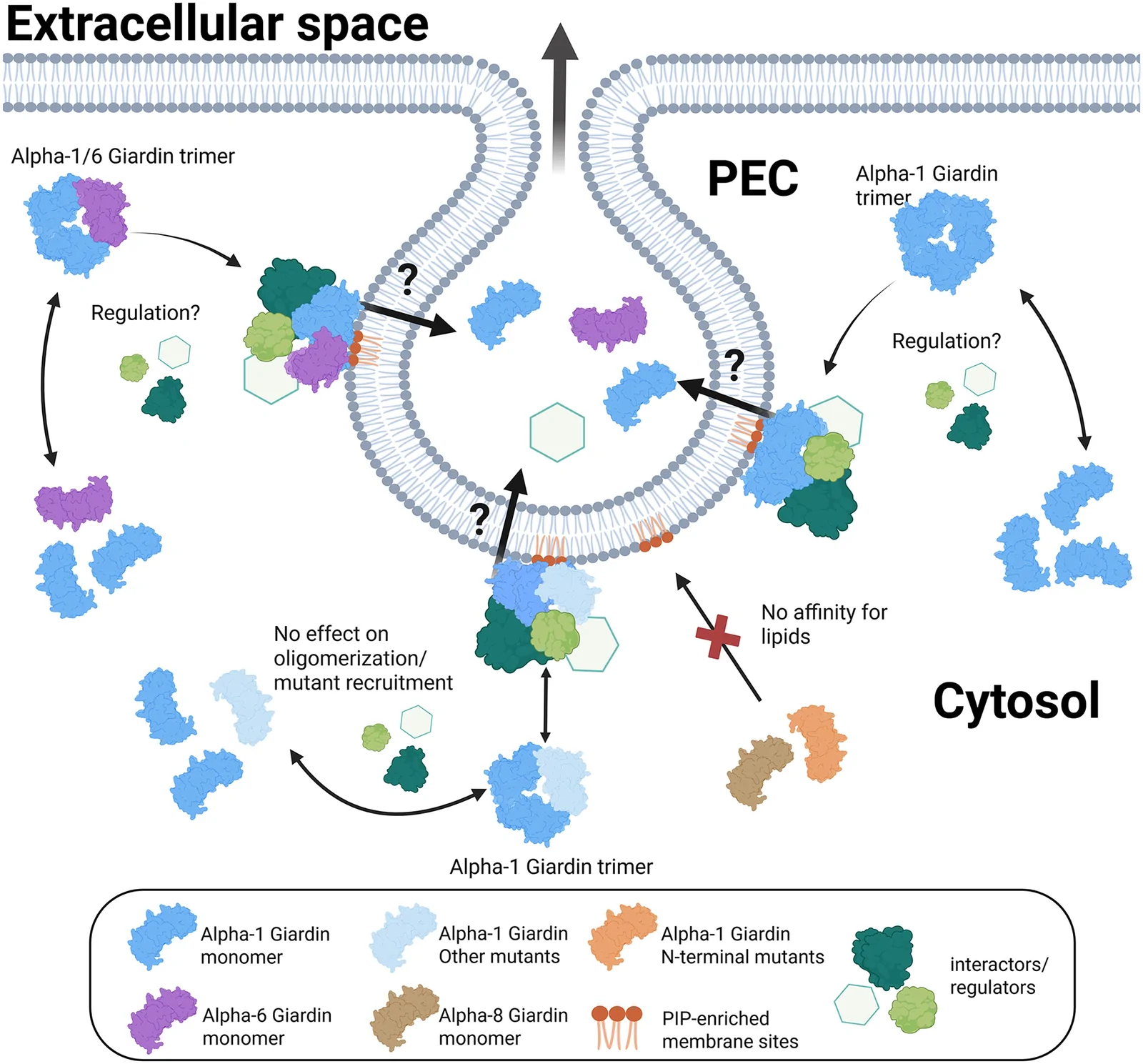
A new working model for PECs-PVs -associated and diffused alpha-giardins. α1G (dark blue) and α6G (violet) are shown as examples of PECs-PVs-associated, whereas α8G (brown) is used as an example of a cytosolic alpha-giardin. Compared to α8G which does not present detectable lipid-binding activity, both α1G and α6G bind lipids and localize to PECs-PVs. The XL-MS data point to the existence of both homo- and hetero- dimers of α1G and α6G. α1G putative lipid binding mutants (light blue) are still detected at PECs-PVs. However, α1G N-terminal variants (burnt orange; either deletion or V5-tagged mutants) mimic α8G in the absence of both PECs-PVs localization and lipid-binding activity. This indicates how the N-terminal region of α1G is an essential functional domain, likely linked to the need for oligomerization to enable lipid binding capacity. These observations are corroborated by MP data presented in this report, showing how α1G N-terminal variants remain monomeric in solution, unlike the non-mutated α1G control. Several open questions remain, including the role for interaction partners previously identified at PECs-PVs -associated complexes (green components) as regulators of oligomerization, followed by lipid binding and mediation of UPS at PECs-PVs [19]. Created in BioRender. Faso, C. (https://BioRender.com/y153lz4) and licensed under CC BY 4.0.

We postulate that αGs at PECs-PVs such as α1G (Fig 10, dark blue) and α6G (Fig 10, violet), in contrast to αGs which are not recruited to these organelles, can oligomerize and, consequently, bind specific lipid residues at PECs-PVs. Higher-order oligomerization, and therefore lipid-binding ability, is dependent on an intact N-terminus which is therefore also ultimately responsible for PECs-PVs localization. In contrast, α8G (Fig 10, brown) is never found at PECs-PVs, nor does it detectably bind lipids, despite MP data compatible with a dimeric state for both α8G and Δ3α1G. Unlike putative α1G lipid-binding mutants (Fig 10, light blue) which are indistinguishable from WT α1G, N-terminal α1G mutants (Fig 10, orange) behave similarly to α8G, further corroborating the essentiality of α1G’s N-terminal region for oligomerization, lipid binding activity, and deposition at PECs-PVs. Several open questions remain, most notably the identity of regulators of αG oligomerization (Fig 10, green components) as a pre-requisite for lipid binding at PECs-PVs, and the role for αGs at PECs-PVs in mediating UPS of selected cargo (Fig 10, green components).

### Why does heterologous expression of α1G fail to give lipid-binding protein?

An immediate question arising from the working model proposed above concerns the nature of the regulatory circuits allowing some αGs to associate to PECs-PVs membranes, while others do not, although virtually indistinguishable on a sequence level. Since the entire family of αGs has not yet been systematically tested for this property, we cannot claim oligomerization as exclusive to PECs-PVs-associated αGs. However, MP and XL-MS data do point towards a major role for oligomerization in functionality and PECs-PVs association, at least for α1G. At the same time, failure to express lipid binding-competent α1G in heterologous systems, both prokaryotic and eukaryotic (S3 Fig), points to a missing *G. lamblia*-specific component to obtain functional protein. This component may either be an essential interaction partner or a dedicated post-translational modification (Fig 10, green components). The MS data point to the presence of several phosphorylated residues which may be integral to protein function which may not be fully recapitulated in heterologous systems in the absence of a specific interaction partner. One such candidate partner regulator could be NEK kinase (ORF GL50803_15411) which, although still classified as a catalytically inactive kinase (as the majority of *G. lamblia* NEKs) [60], may act as a scaffold for other (active) kinases in *G. lamblia*. In a previous study, N-terminally tagged α1G expressed in *E. coli* was reported to successfully bind glycosaminoglycan [34]. This report is compatible with our data while also showing how the two ends of this protein perform different and independent functions which are differentially impacted by attempts at heterologous expression.

### A comparison of α1G and G. lamblia-expressed human anxA5

Epitope-tagged human anxA5 expressed in *G. lamblia* localizes to the periphery of the cell and binds negatively charged lipids. This corroborates the observation that only αGs at PECs-PVs can bind lipids, as also shown using lipid strip experiments. AnxA5 was shown to organize in trimers at the membrane [58], and its oligomerization depends on PTMs, negatively charged lipids and calcium concentration [54]. This last variable is perhaps the most conspicuous difference between α1G, anxA5 and generally most annexins. In this sense, α1G is an unusual lipid-binding annexin at unusual endocytic compartments, with unusual requirements for calcium [35]. However, the common need for oligomerization is a shared feature which points once more to the importance of this structural property.

### MD simulation of protein-membrane interactions highlights the role of PIPs and predicts residues important for membrane association

Amongst the amino acid residues highlighted by MD simulations as the most promising sites of α1G-membrane interaction, Lys11, Lys55 and Arg82 deserve particular attention. At higher PIP concentrations, each of these residues displayed significantly low K_*off*_ values suggesting that their interactions are likely critical for stabilising membrane binding (S4 Fig). According to the simulations, Arg82 and Lys55 are the most critical residues across all PIP-containing systems. Lys55 interacted with PIPs across all tested membrane compositions, with its contribution becoming more pronounced as PIP levels increased. On the other hand, Arg82 was observed to bind only to PIPs at higher concentrations, forming a stronger, more durable interaction. Additionally, Arg82 formed relatively strong interactions with PS, which further stabilized the interaction with the membrane. Collectively, these observations position Arg82 and Lys55 as the most likely crucial residues to interact with PIP in α1G. In fact, most interacting residues can be classified as mixed binders, as they engage with both PIPs and PS to varying extents. However, a subset of residues appears to preferentially bind PIPs, meeting the criterion of PS K_*off*, min_ - PIP K_*off*, max_ > 50 µs ⁻ ¹. These PIP-selective residues include Lys47, Lys58 (based on standard K_*off*_ values), Lys127 and Lys119. Taken together, these data are important to further dissect α1G lipid binding properties by narrowing down residue selection for site-directed mutagenesis and *in vitro* validation efforts.

## Conclusion

In conclusion, the data presented here highlight the critical role of the N-terminus of α1G, a promising giardiasis vaccine candidate, in its oligomerization, lipid-binding ability, and correct localization to PECs-PVs. The proposed model suggests that αGs such as α1G require an intact N-terminus for effective oligomerization and lipid interaction at PECs-PVs. Moreover, the inability to express lipid-binding competent α1G in heterologous systems points to a potential *G. lamblia*-specific component that is crucial for functionality, possibly involving specific post-translational modifications or interaction partners. The comparison with anxA5 further emphasizes the importance of oligomerization in membrane binding, highlighting unique characteristics of α1G in lipid binding at PECs-PVs. The data underscore the complex and essential role of α1G in the parasite’s biology; any insights in its structural and functional determinants, as presented in this report, provide additional information towards the fulfilment of α1G’s potential as a highly promising giardiasis vaccine candidate.

## Materials and methods

### Construct generation, transformation and cell culture conditions

The DNA constructs used in this study were generated by PCR, followed by ligation into expression vectors using either T4 DNA ligase or Gibson assembly. The sequences of the oligonucleotides and all the details of the reactions are summarized in S7 Data while all construct sequences are provided in FASTA and APE formats in S8 Data**.** Schematics for the constructs are shown in S1 Fig.

*G. lamblia* trophozoites were cultured in previously well-described axenic conditions [19,40,42]. Cells were transfected via electroporation with circular plasmid vectors and selected with the antibiotic puromycin to generate episomal transgenic lines. Immuno-blotting as well as IFAs (immunofluorescence assays) were used to check expression levels of the transfected constructs.

### Immunofluorescence assay

IFAs were performed according to previously published protocols [19,42]. Trophozoites, WT or expressing the epitope tagged protein of interest, were grown in 12ml Nunc polystyrene culture tubes (ThermoFisher Scientific). Per IFA one such tube per line was grown until the cells were forming a confluent layer. The tubes were then incubated on ice for a minimum of 30 min to detach the cells. The cells were then pelleted at 700g for 10 min, washed in PBS (phosphate-buffered saline) and fixed in 3% formaldehyde (Sigma) in PBS for a minimum of one hour or overnight. Fixation was followed by quenching in 0.1M Glycine in PBS and permeabilization in 2% bovine serum albumin (BSA) + 0.2% Triton-X-100 in PBS. For antibody staining the cells were first incubated for one hour in 2% BSA + 0.1% Triton-X-100 in PBS containing the rat-derived monoclonal primary anti-HA antibody (Roche) diluted 1:250. Then the samples were washed twice in 1% BSA + 0.05% Triton-X-100 in PBS (wash solution). The secondary antibody was diluted 1:250 in 2% BSA + 0.1% Triton-X-100 in PBS. A goat-derived anti-rat IgG (H + L) conjugated to Alexa Fluor 488 (ThermoFisher) was used for incubation in the dark at room temperature. After the second incubation cells were again washed twice in wash solution and then resuspended in Vectashield embedding medium (Reactolab) containing DAPI (4′-6-diamidino-2-phenylindole) as a DNA label. Imaging was performed at a Leica SP8 confocal microscope configured with white light lasers.

### Co-immunoprecipitation (co-IP) in native conditions and DSSO crosslinking

Native co-IPs were performed as previously described [19]. *G. lamblia* trophozoites expressing a tagged reporter construct of interest or WT control WB were grown in two T25 culture flask per line over two days until confluency was reached on both sides of the flask (turned after the first 24 hours). For harvesting, the cultures were placed on ice for a minimum of one hour for the cells to detach before they were transferred to 50ml Falcon tubes. Cells were centrifuged and resuspended in 10ml PBS with addition of protease inhibitors (Sigma) and 1% Triton-X-100. Cells were burst using sonication and then incubated in the cold for two hours with gentle rotation. Samples were transferred to Eppendorf tubes and centrifuged at 16000g for 10 min at 4°. Supernatants were filtered through 0.2μm PVDF membrane syringe filters and incubated with anti-HA agarose beads (Thermo Fisher Scientifc) for protein pulldown according to manufacturer’s instructions (40ul slurry/extract from a T25 flask). Following the overnight incubation, the beads were washed 3x with PBS + 0.1% Triton-X-100 and 3x with PBS. For simple co-IP, beads were stored frozen before analysis. For DSSO crosslinking, beads corresponding to one flask per line were resuspended in 500ul PBS and 10ul DSSO in DMSO (50mM) was added to reach approximately a 100-fold molar excess of crosslinker over the protein concentration. After an incubation of 45 minutes at room temperature, the reaction was stopped with Tris buffer (resulting in a final Tris concentration of 0.1M) for 5 min and the beads were washed 3x with PBS and stored frozen before analysis. All bead samples were handed over to the Proteomics and Mass Spectrometry Core Facility of the University of Bern for tryptic digests and peptide analysis.

### Liquid chromatography mass spectrometry for native and DSSO crosslinked samples for XL-MS

Proteins captured on the pull-down beads were denatured in 30 μL 8 M urea, 50 mM Tris/HCl pH 8, and cysteines alkylated with 50 mM iodoacetamide for 30 min in the dark after reduction with 10 mM DTT for 30 min at 37°C. The urea concentration was lowered by adding 60 μL 20mM Tris/HCl pH 8 containing 2 mM CaCl_2_, followed by digestion with 100 ng LysC for 2 hours at 37°C. Urea was further diluted to 1.6 M and digestion continued by addition of 100 ng trypsin over night at room temperature. After acidification with 1% TFA end concentration, aliquots of 5 μL were analyzed by nano-liquid reversed phase chromatography coupled to tandem mass spectrometry on an Orbitrap Fusion LUMOS mass spectrometer that was coupled with a Dionex Ultimate 3000 nano-UPLC system (ThermoFischer Scientific, Reinach, Switzerland). For non-crosslinked samples a standard data-dependent acquisition method as described elsewhere [61] was used with a homemade C18 CSH Waters separation column (1.7 μm, 130 Å, 75 μm × 20 cm) at a flow rate of 250 nL/min. The cross-linked samples were analyzed on the same system using a MS2-MS3-MS2 method. Full MS scans in the orbitrap in the range of 375–1500 m/z were acquired every 5 s. In between data-dependent CID MS2 scans on peptide ions with charge 3–8 were acquired in the orbitrap with resolution 30’000, a fixed relative collision energy of 25%, precursor isolation window of 1.6 m/z, and a MIT of 54 ms. Two MS3 CID fragmentations were triggered on MS2 fragments which formed an ion pair spaced by the DSSO-specific 31.9721 Da at a mass tolerance of 10ppm. Relative collision energy was set at 35%, normalized automatic gain control to 200%, and MIT to 120 ms. If no ion pair was detected in the MS2 CID spectrum another MS2 scan with HCD fragmentation was recorded in the orbitrap at a relative collision energy of 30% and MIT of 100 ms. The non-cross-linked data was interpreted with MaxQuant (version 2.3.1.0) applying strict trypsin cleavage rule with maximum 3 missed cleavages, carbamidomethylation of cysteines as fixed, oxidation of methionines and protein n-terminal acetylation as variable modifications allowing a maximum of three modifications per peptide, a first round search precursor mass tolerance of 15 ppm, and fragment mass tolerance of 0.4 Da, against the *Giardia lamblia* Assemblage AWB protein sequence database from *Giardia*DB (version 55) together with a small database of 230 proteins commonly found as contaminants and the reversed versions of both databases. A false discovery rate on the peptide and protein level of 1% was applied and only proteins identified with at least two unique peptides were accepted. The data of the crosslinked samples were interpreted against the same protein sequence database using Proteome Discoverer version 3.1.0.638 with built-in Sequest HT and XlinkX again using a decoy search strategy to control the false discovery rate at 1% for non- and cross-linked peptide identifications. The mass spectrometry proteomics data have been deposited to the ProteomeXchange Consortium (http://proteomecentral.proteomexchange.org) via the PRIDE partner repository [62] with the dataset identifier PXD074377 and DOI 10.6019/PXD074377 (S9 Data).

### Lipid strip protein binding assay

Proteins were obtained by anti-HA-agarose beads pulldown as described in the co-IP section. Afterwards, the beads were eluted with 2mg/ml HA peptide (MedChemExpress) in PBS.

Membranes spotted with a selection of lipids (Echelon Biosciences P-6002 and P-6100) were used for lipid binding assays according to manufacturer’s protocols with each of several αG complexes prepared as described above. The membranes were incubated in water for 5 minutes followed by a 1h blocking step in PBS containing 3% lipid-free BSA (Sigma). Protein complexes were added and incubated for 1 h. Membranes were washed 3x10min with PBS/Tween 0.05% and then incubated for 1h in PBS containing primary antibody (rat-derived monoclonal anti-HA, Roche). Membranes were washed again, incubated in secondary antibody (goat anti-rat coupled to HRP, Sigma) before a last set of washes. Membranes were then imaged using enhanced chemiluminescence (ECL, ThermoScientific). No calcium ions were added at any step of the lipid strip binding protocol when testing αGs.

Testing of anxA5 lipid binding affinity was done using the same protocol, except for the use of HEPES 25mM, pH 7 instead of PBS, with or without calcium ion supplementation (CaCl_2_).

### Structural reconstruction using XL-MS data

XL-MS data was filtered for identified intra- and interlinks with a threshold of Delta XlinkX Score ≥ 50, using the Delta XlinkX Score as a proxy for hit-reliability [63] An interactome network was generated using the Cytoscape software based on found interlinks. Found interlinks between alpha-1 giardin and alpha-6 giardin and Xaa-pro dipeptidase in the XL-MS data were visualised as linear maps informed by data on protein motifs from *Giardia*DB. To reconstruct the 3D structure of the identified associated proteins alpha-1- and alpha-6 giardin and Xaa-pro dipeptidase, structural models were sourced from alphafold.ebi.ac.uk or predicted using AlphaFold webserver [64]. The HADDOCK 2.4 web server interface [65,66] was then used to integrate our own generated post-capture XL-MS data with the sourced 3D models of the individual protein structures to construct a comprehensive model of the 3D structure of these three interactors. The resulting structure was subsequently refined and merged using ChimeraX [67], GIMP [68] and Cytoscape [69].

### Mass photometry

Mass photometry experiments were conducted using a Refeyn TwoMP system (Refeyn Ltd., Oxford, UK) equipped with the AcquireMP and DiscoverMP software packages for data acquisition and analysis, respectively, under standard settings. For the experiments, high-precision microscope coverslips, sourced from Refeyn, were used as single-use items. To preserve the droplet shape of the sample, self-adhesive silicone culture wells (Grace Bio-Labs reusable CultureWell gaskets) were employed. αG samples and WB were diluted in PBS, while anxA5 samples were prepared in 20 mM HEPES (pH 7.5), 300 mM NaCl, and the appropriate concentration of CaCl₂.

For contrast-to-mass calibration, Bovine Serum Albumin Fraction V Low Heavy Metals (Millipore) oligomers with molecular weights of 66, 132 and 198 kDa were utilized. Prior to measurements, protein stocks were diluted in stock buffers containing 20 mM HEPES (pH 7.5) and 300 mM NaCl for anxA5 experiments or PBS for αG measurements. Specifically, 2 uL of the protein solution was mixed with 18 uL of analysis buffer, yielding a final droplet volume of 20 uL with a protein concentration of approximately 1 ug/mL.

### Protein modelling and initial structure preparation

To investigate the interaction between α1G and lipid bilayers, a trimeric model of α1G was constructed. The monomeric structure was predicted using the AlphaFold web server [64], excluding the N-terminal methionine (Met1) to reflect the mature protein form, resulting in all residue numbers in the molecular dynamics simulations being shifted by −1. Based on XL-MS data identifying interaction sites, a trimeric configuration was assembled using the HADDOCK v2.4 docking platform [65,66]. The trimer was selected for simulation based on evidence from mass spectrometry indicating its oligomeric state.

Five distinct membrane systems were constructed using the CHARMM-GUI Membrane Builder webserver [70–80], with each consisting of approximately 350 lipid molecules per leaflet. All systems included PI(3)P and PI(5)P lipids at varying concentrations to assess their influence on α1G binding. The exact lipid compositions for each system are summarized in S6 Data. Water molecules were removed from the unit cell, and the α1G trimer was positioned above the membrane at a minimal distance of 10 Å from the surface to allow for potential interaction while avoiding initial steric clashes.

### Molecular dynamics simulations

The membrane system preparation was continued in CHARMM-GUI, with all components parameterized using the CHARMM36 force field, generating GROMACS-compatible files [70–81].

The TIP3P water model was used to solvate each system in a simulation box of averaged dimensions 170 × 140 × 140 Å, containing approximately 350000 atoms. Appropriate numbers of Na⁺ and Cl⁻ counterions were added to ensure electrostatic neutrality and physiological ionic strength (~0.15 mM).

Energy minimization was performed using the steepest descent algorithm, with a convergence Vmax of 1000 kJ·mol ⁻ ¹·nm ⁻ ¹. Partial equilibration was conducted on the CHARMM GUI server using the NVT and NPT ensembles to gradually relax positional restraints on the thermos-coupled components of the systems. Electrostatic interactions were treated using the particle mesh Ewald (PME) method, while van der Waals forces were computed via a force-switch cutoff scheme with a switching distance of 1.0 nm and a cutoff of 1.2 nm. Temperature coupling was applied separately to the solute, membrane, and solvent groups using the V-rescale thermostat with a reference temperature of 310 K and a coupling constant of 1.0 ps. Pressure was maintained at 1 bar using the C-rescale barostat with semi-isotropic coupling, a compressibility of 4.5 × 10 ⁻ ⁵ bar ⁻ ¹, and a relaxation time of 5.0 ps. Finally, maintaining the same barostat, production runs of 200 ns were carried out using GROMACS 2023.3 [82,83] with a leap-frog integrator and a time step of 2 fs, applying the LINCS algorithm to constrain all hydrogen-containing bonds. All simulations, including the final equilibration and production runs, were performed using 1 CPU core and GPU RTX4090 of University of Bern cluster UBELIX. Calculations were performed on UBELIX (https://www.id.unibe.ch/hpc), the HPC cluster at the University of Bern. Protein-lipid interaction was performed with the Python library PyLipID [84] with a dual cutoff of 0.15 nm – 0.25nm and bootstrapping of K_*off*_ values [84]. The dual cutoff values were selected based on the protocol in the PyLipID library documentation. Bootstrapping was automatically run by the PyLipID library based on duplicate simulations. Residues were selected based on K_*off*_ values suggested by Song et al. The duration and occupancy thresholds were set in proportion to the total simulation time and matched with the interaction behavior we wanted to focus on.

## Supporting information

S1 Fig Schematic maps of the plasmids used in this study. A. Vector backbone used for generation of transgenic Giardia lines G25, G26, G65 and G67. The respective ORF and its own promoter were amplified from giardia genomic DNA and cloned using restriction enzymes XbaI and PacI. ORFs for G222, G232 and G233 were cloned in the same way after introducing point mutations. B. Vector backbone used for generation of transgenic Giardia lines G183, G190-194, G196-G206, G219, G220, G282-285 and G455. The respective ORF was cloned downstream of the α1G promoter using restriction enzymes HpaI and PacI. C. Plasmid used to generate yeast line Y489. The codon-optimized α1G ORF was assembled downstream of the yeast PGK promoter and cloned using restriction enzymes PacI and XhoI. D. Plasmid for protein expression in bacteria. The α1G ORF was introduced into vector pTXB1 using restriction enzymes EcoRI and SapI, followed by the removal of a short sequence upstream of the ATG starting codon. Refer to S7 Data for all cloning details and to S8 Data for sequences in both.txt and.APE format.
(TIF)

S2 Fig Site-directed mutagenesis analysis of α1G. Lines G220, 222, 232, 233 and 248 were developed to analyse putative α1G lipid binding mutants at key lysine (K) and arginine (R) residues. All analysed mutant reporters still appear to deposit at the periphery of the cell, similar to non-mutated α1G. Scale bar: 5um.
(TIF)

S3 Fig Heterologous expression tests for epitope-tagged α1G in *E. coli* and *S. cerevisiae.* Both systems yield sufficient protein; however, lipid binding functionality is not maintained.
(TIF)

S4 Fig K_*off*_ values for selected lipid interaction residues. A. K_*off*_ values across all performed simulations are presented as a range of the highest and lowest values obtained. B. Bootstrapped K_*off*_ values. All K_*off*_ values and bootstrapping were calculated using a PyLipID library based on duplicate MD simulations (S6 Data).
(TIF)

S1 Data Native co-IP results for transgenic *G. lamblia* lines overexpressing HA-tagged α1G, 6G, 8G and 11G, used as baits for protein pull-down. (XLSX)

S2 Data XL_MS results for several transgenic *G. lamblia* lines and their respective HA-tagged baits. The complete data sets are provided, along with a summary of the significant inter- and intralinks grouped by bait.
(XLSX)

S3 Data Raw mass photometry data for all measurements in this study. The file includes all calibrations, sample measurements and buffer controls.
(XLSX)

S4 Data Native co-IP results for transgenic *G. lamblia* lines G282 and G283 overexpressing HA-tagged α1G and the Δ3α1G N-terminal mutant, respectively, used as baits for protein pull-down. (XLSX)

S5 Data XL-MS results for the pull-down of HA-tagged Δ3α1G complexes in line G283. (XLSX)

S6 Data Protein–lipid interaction molecular simulation data generated using the PyLipID Python library. (XLSX)

S7 Data Cloning details and oligonucleotide sequences for construct design and generation. (XLSX)

S8 Data Plasmid and construct sequences in FASTA and APE formats. (ZIP)

S9 Data Summary of proteomics datasets deposited to the ProteomeXchange Consortium via the PRIDE partner repository [62], with the dataset identifier PXD074377 and DOI 10.6019/PXD074377. (XLSX)

S1 File Raw unprocessed immunoblotting images for data shown in Figs 3, 6, 7 and S3. (PDF)

S1 Video Live cell imaging transgenic Giardia cells expressing free mNG, line G369. These data can be viewed at Dryad, https://doi.org/10.5061/dryad.02v6wwqk4.
(GIF)

S2 Video Live cell imaging of full-length α1G fused to mNeonGreen (mNG) at the C terminus, line G370. These data can be viewed at Dryad, https://doi.org/10.5061/dryad.02v6wwqk4.
(GIF)

S3 Video Live cell imaging of an mNG C-terminally tagged version of the α1G variant with a deletion of the first three amino acid residues (Δ3α1G), line G390. These data can be viewed at Dryad, https://doi.org/10.5061/dryad.02v6wwqk4.
(GIF)

S4 Video Live cell imaging of an mNG C-terminally tagged α8G variant, line G390. These data can be viewed at Dryad, https://doi.org/10.5061/dryad.02v6wwqk4.
(GIF)

S5 Video First molecular dynamics (MD) simulation for the interaction of the potentially native α1G trimer with base membrane containing phosphatidylcholine (PC), phosphatidylserine (PS), and cholesterol, viewed from the side; a1gtrimerdel1Met_base_meme_1_side. Colour legend: PS – Yellow; Other lipids – Grey; Interacting residues – White. These data can be viewed at Dryad, https://doi.org/10.5061/dryad.02v6wwqk4.
(GIF)

S6 Video First MD simulation for the interaction of the potentially native α1G trimer with base membrane containing phosphatidylcholine (PC), phosphatidylserine (PS), and cholesterol, viewed from the top; a1gtrimerdel1Met_base_meme_1_top. Colour legend: PS – Yellow; Other lipids – Grey; Interacting residues – White. These data can be viewed at Dryad, https://doi.org/10.5061/dryad.02v6wwqk4.
(GIF)

S7 Video Second molecular dynamics (MD) simulation for the interaction of the potentially native α1G trimer with base membrane containing phosphatidylcholine (PC), phosphatidylserine (PS), and cholesterol, viewed from the side; a1gtrimerdel1Met_base_meme_2_side. Colour legend: PS – Yellow; Other lipids – Grey; Interacting residues – White. These data can be viewed at Dryad, https://doi.org/10.5061/dryad.02v6wwqk4.
(GIF)

S8 Video Second MD simulation for the interaction of the potentially native α1G trimer with base membrane containing phosphatidylcholine (PC), phosphatidylserine (PS), and cholesterol, viewed from the top; a1gtrimerdel1Met_base_meme_2_top. Colour legend: PS – Yellow; Other lipids – Grey; Interacting residues – White. These data can be viewed at Dryad, https://doi.org/10.5061/dryad.02v6wwqk4.
(GIF)

S9 Video First MD simulation for the interaction of the potentially native α1G trimer with base membrane containing phosphatidylcholine (PC), phosphatidylserine (PS), and cholesterol, supplemented with 3% PI(3)P, viewed from the side; a1gtrimerdel1Met_3percPI3P_1_side. Colour legend: PIPs – Orange; PS – Yellow; Other lipids – Grey; Interacting residues – White. These data can be viewed at Dryad, https://doi.org/10.5061/dryad.02v6wwqk4.
(GIF)

S10 Video First MD simulation for the interaction of the potentially native α1G trimer with base membrane containing phosphatidylcholine (PC), phosphatidylserine (PS), and cholesterol, supplemented with 3% PI(3)P, viewed from the top; a1gtrimerdel1Met_3percPI3P_1_top. Colour legend: PIPs – Orange; PS – Yellow; Other lipids – Grey; Interacting residues – White. These data can be viewed at Dryad, https://doi.org/10.5061/dryad.02v6wwqk4.
(GIF)

S11 Video Second MD simulation for the interaction of the potentially native α1G trimer with base membrane containing phosphatidylcholine (PC), phosphatidylserine (PS), and cholesterol, supplemented with 3% PI(3)P, viewed from the side; a1gtrimerdel1Met_3percPI3P_2_side. Colour legend: PIPs – Orange; PS – Yellow; Other lipids – Grey; Interacting residues – White. These data can be viewed at Dryad, https://doi.org/10.5061/dryad.02v6wwqk4.
(GIF)

S12 Video Second MD simulation for the interaction of the potentially native α1G trimer with base membrane containing phosphatidylcholine (PC), phosphatidylserine (PS), and cholesterol, supplemented with 3% PI(3)P, viewed from the top; a1gtrimerdel1Met_3percPI3P_2_top. Colour legend: PIPs – Orange; PS – Yellow; Other lipids – Grey; Interacting residues – White. These data can be viewed at Dryad, https://doi.org/10.5061/dryad.02v6wwqk4.
(GIF)

S13 Video First MD simulation for the interaction of the potentially native α1G trimer with base membrane containing phosphatidylcholine (PC), phosphatidylserine (PS), and cholesterol, supplemented with 3% PI(5)P, viewed from the side; a1gtrimerdel1Met_3percPI5P_1_side. Colour legend: PIPs – Orange; PS – Yellow; Other lipids – Grey; Interacting residues – White. These data can be viewed at Dryad, https://doi.org/10.5061/dryad.02v6wwqk4.
(GIF)

S14 Video. First MD simulation for the interaction of the potentially native α1G trimer with base membrane containing phosphatidylcholine (PC), phosphatidylserine (PS), and cholesterol, supplemented with 3% PI(5)P, viewed from the top; a1gtrimerdel1Met_3percPI5P_1_top. Colour legend: PIPs – Orange; PS – Yellow; Other lipids – Grey; Interacting residues – White. These data can be viewed at Dryad, https://doi.org/10.5061/dryad.02v6wwqk4.
(GIF)

S15 Video Second MD simulation for the interaction of the potentially native α1G trimer with base membrane containing phosphatidylcholine (PC), phosphatidylserine (PS), and cholesterol, supplemented with 3% PI(5)P, viewed from the side; a1gtrimerdel1Met_3percPI5P_2_side. Colour legend: PIPs – Orange; PS – Yellow; Other lipids – Grey; Interacting residues – White. These data can be viewed at Dryad, https://doi.org/10.5061/dryad.02v6wwqk4.
(GIF)

S16 Video Second MD simulation for the interaction of the potentially native α1G trimer with base membrane containing phosphatidylcholine (PC), phosphatidylserine (PS), and cholesterol, supplemented with 3% PI(5)P, viewed from the top; a1gtrimerdel1Met_3percPI5P_2_top. Colour legend: PIPs – Orange; PS – Yellow; Other lipids – Grey; Interacting residues – White. These data can be viewed at Dryad, https://doi.org/10.5061/dryad.02v6wwqk4.
(GIF)

S17 Video First MD simulation for the interaction of the potentially native α1G trimer with base membrane containing phosphatidylcholine (PC), phosphatidylserine (PS), and cholesterol, supplemented with 12% PI(3)P, viewed from the side; a1gtrimerdel1Met_12percPI3P_1_side. Colour legend: PIPs – Orange; PS – Yellow; Other lipids – Grey; Interacting residues – White. These data can be viewed at Dryad, https://doi.org/10.5061/dryad.02v6wwqk4.
(GIF)

S18 Video First MD simulation for the interaction of the potentially native α1G trimer with base membrane containing phosphatidylcholine (PC), phosphatidylserine (PS), and cholesterol, supplemented with 12% PI(3)P, viewed from the top; a1gtrimerdel1Met_12percPI3P_1_top. Colour legend: PIPs – Orange; PS – Yellow; Other lipids – Grey; Interacting residues – White. These data can be viewed at Dryad, https://doi.org/10.5061/dryad.02v6wwqk4.
(GIF)

S19 Video Second MD simulation for the interaction of the potentially native α1G trimer with base membrane containing phosphatidylcholine (PC), phosphatidylserine (PS), and cholesterol, supplemented with 12% PI(3)P, viewed from the side; a1gtrimerdel1Met_12percPI3P_2_side. Colour legend: PIPs – Orange; PS – Yellow; Other lipids – Grey; Interacting residues – White. These data can be viewed at Dryad, https://doi.org/10.5061/dryad.02v6wwqk4.
(GIF)

S20 Video Second MD simulation for the interaction of the potentially native α1G trimer with base membrane containing phosphatidylcholine (PC), phosphatidylserine (PS), and cholesterol, supplemented with 12% PI(3)P, viewed from the top; a1gtrimerdel1Met_12percPI3P_2_top. Colour legend: PIPs – Orange; PS – Yellow; Other lipids – Grey; Interacting residues – White. These data can be viewed at Dryad, https://doi.org/10.5061/dryad.02v6wwqk4.
(GIF)

S21 Video First MD simulation for the interaction of the potentially native α1G trimer with base membrane containing phosphatidylcholine (PC), phosphatidylserine (PS), and cholesterol, supplemented with 12% PI(5)P, viewed from the side; a1gtrimerdel1Met_12percPI5P_1_side. Colour legend: PIPs – Orange; PS – Yellow; Other lipids – Grey; Interacting residues – White. These data can be viewed at Dryad, https://doi.org/10.5061/dryad.02v6wwqk4.
(GIF)

S22 Video First MD simulation for the interaction of the potentially native α1G trimer with base membrane containing phosphatidylcholine (PC), phosphatidylserine (PS), and cholesterol, supplemented with 12% PI(5)P, viewed from the top; a1gtrimerdel1Met_12percPI5P_1_top. Colour legend: PIPs – Orange; PS – Yellow; Other lipids – Grey; Interacting residues – White. These data can be viewed at Dryad, https://doi.org/10.5061/dryad.02v6wwqk4.
(GIF)

S23 Video Second MD simulation for the interaction of the potentially native α1G trimer with base membrane containing phosphatidylcholine (PC), phosphatidylserine (PS), and cholesterol, supplemented with 12% PI(5)P, viewed from the side; a1gtrimerdel1Met_12percPI5P_2_side. Colour legend: PIPs – Orange; PS – Yellow; Other lipids – Grey; Interacting residues – White. These data can be viewed at Dryad, https://doi.org/10.5061/dryad.02v6wwqk4.
(GIF)

S24 Video Second MD simulation for the interaction of the potentially native α1G trimer with base membrane containing phosphatidylcholine (PC), phosphatidylserine (PS), and cholesterol, supplemented with 12% PI(5)P, viewed from the top; a1gtrimerdel1Met_12percPI5P_2_top. Colour legend: PIPs – Orange; PS – Yellow; Other lipids – Grey; Interacting residues – White. These data can be viewed at Dryad, https://doi.org/10.5061/dryad.02v6wwqk4.
(GIF)
