# Supplementary material for: The N-terminus of alpha-1 giardin, a parasitic annexin orthologue, is essential for oligomerization and lipid-binding activity
Source: PLoS Pathog. 2026 Oct 14;22(10):e1014407. doi: 10.1371/journal.ppat.1014407 (PMC13686171; doi:10.1371/journal.ppat.1014407)
Supplement: Supplementary file 1 [file ppat.1014407.s014.pdf]

Raw image files for blots

Alpha-1 giardin (G25), Fig 3

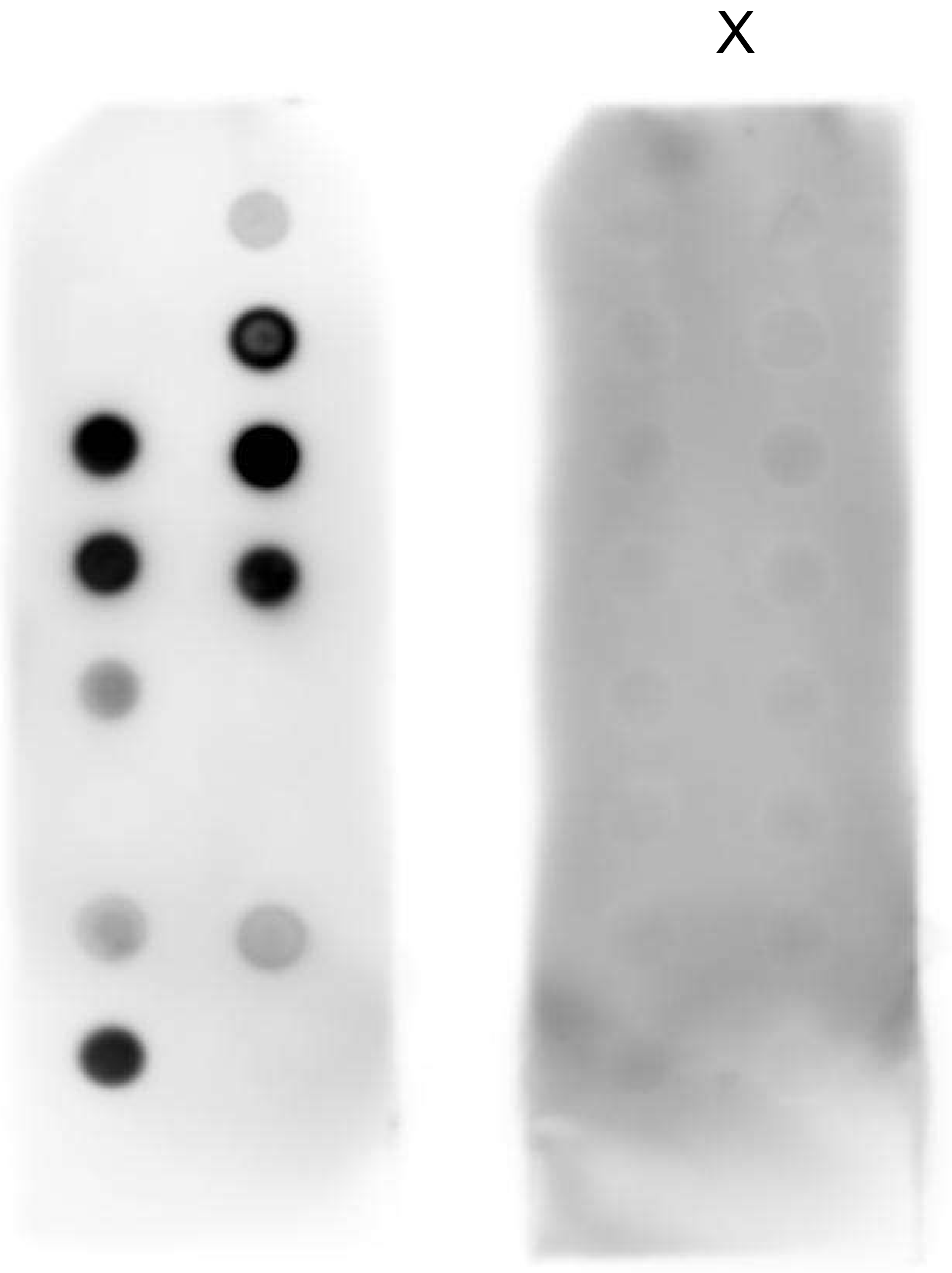

Chemiluminescence

Alpha-1 giardin (G25) PIP array, Fig 3

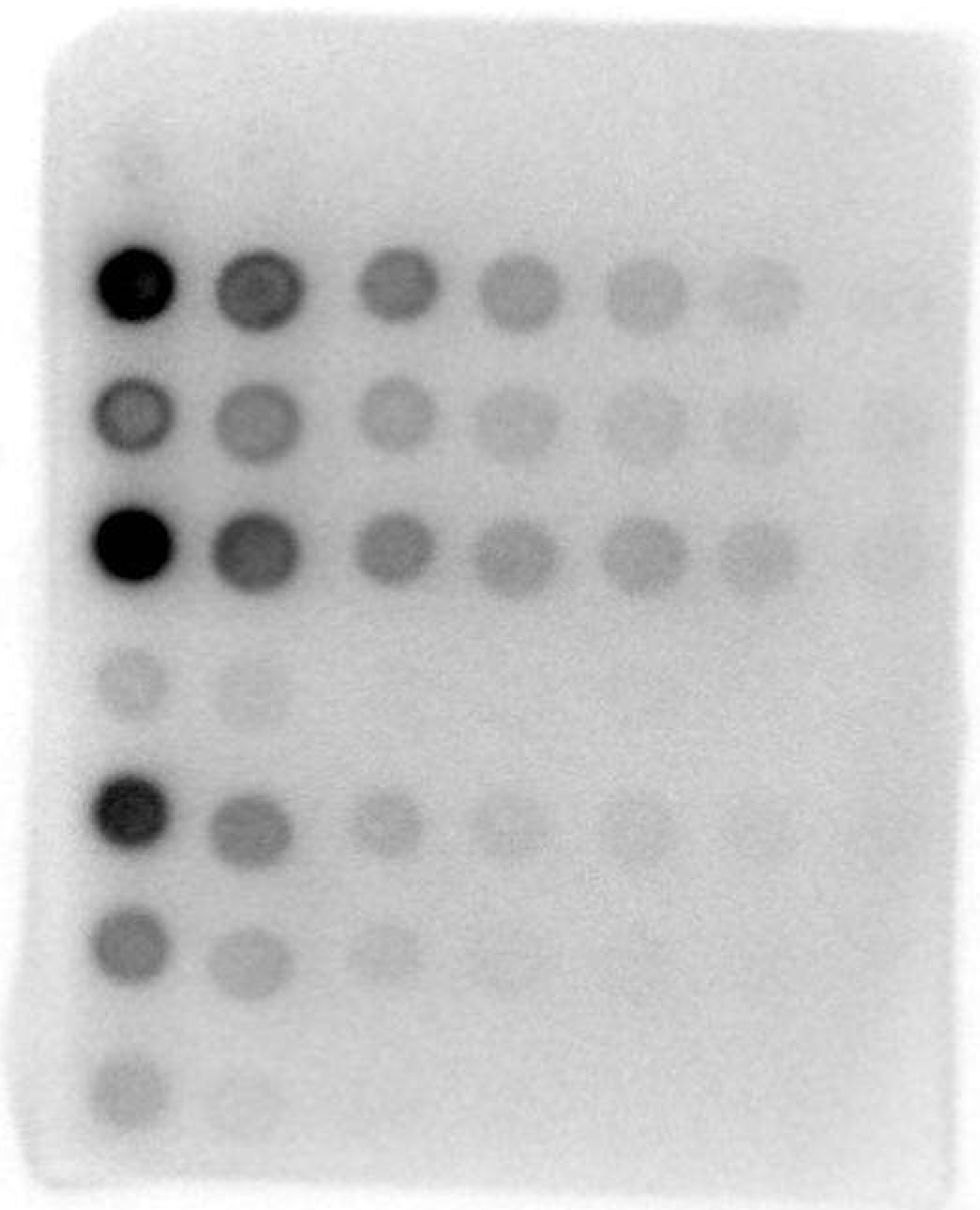

Chemiluminescence

Alpha-6 giardin (G183), Fig 3

X

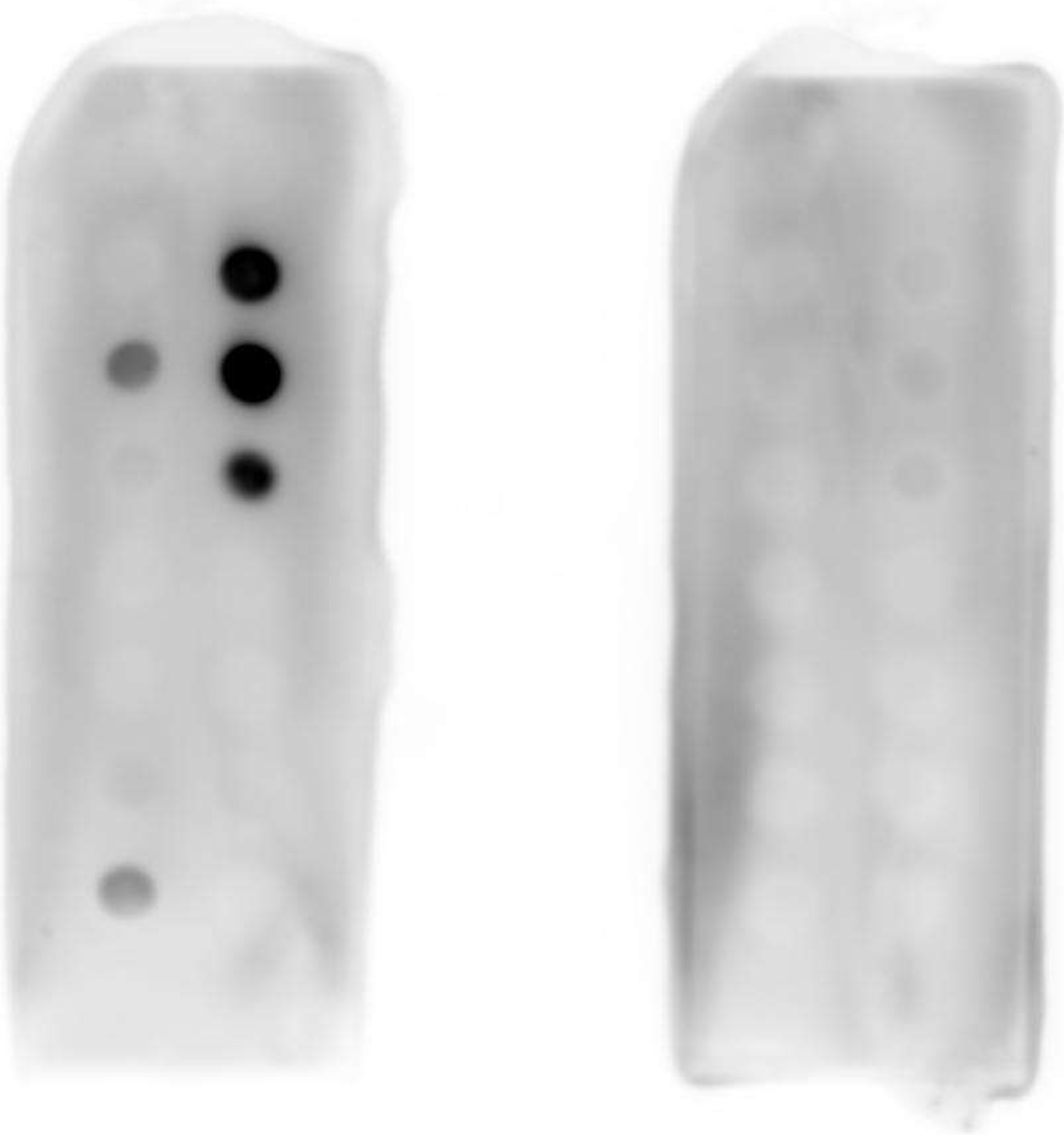

Chemiluminescence

Alpha-2 giardin (G189), Fig 3

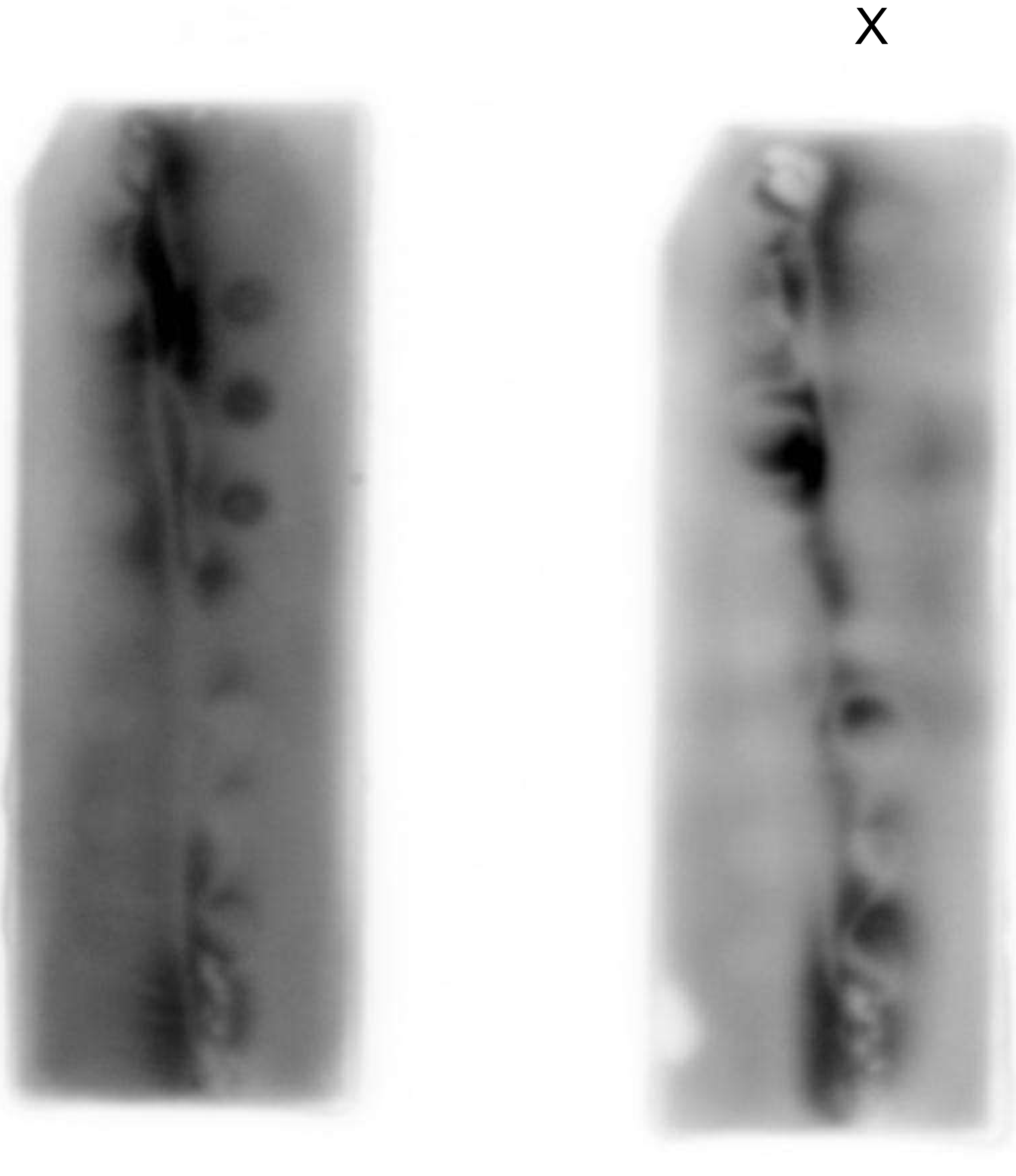

Chemiluminescence

Alpha-11 giardin (G190), Fig 3

X

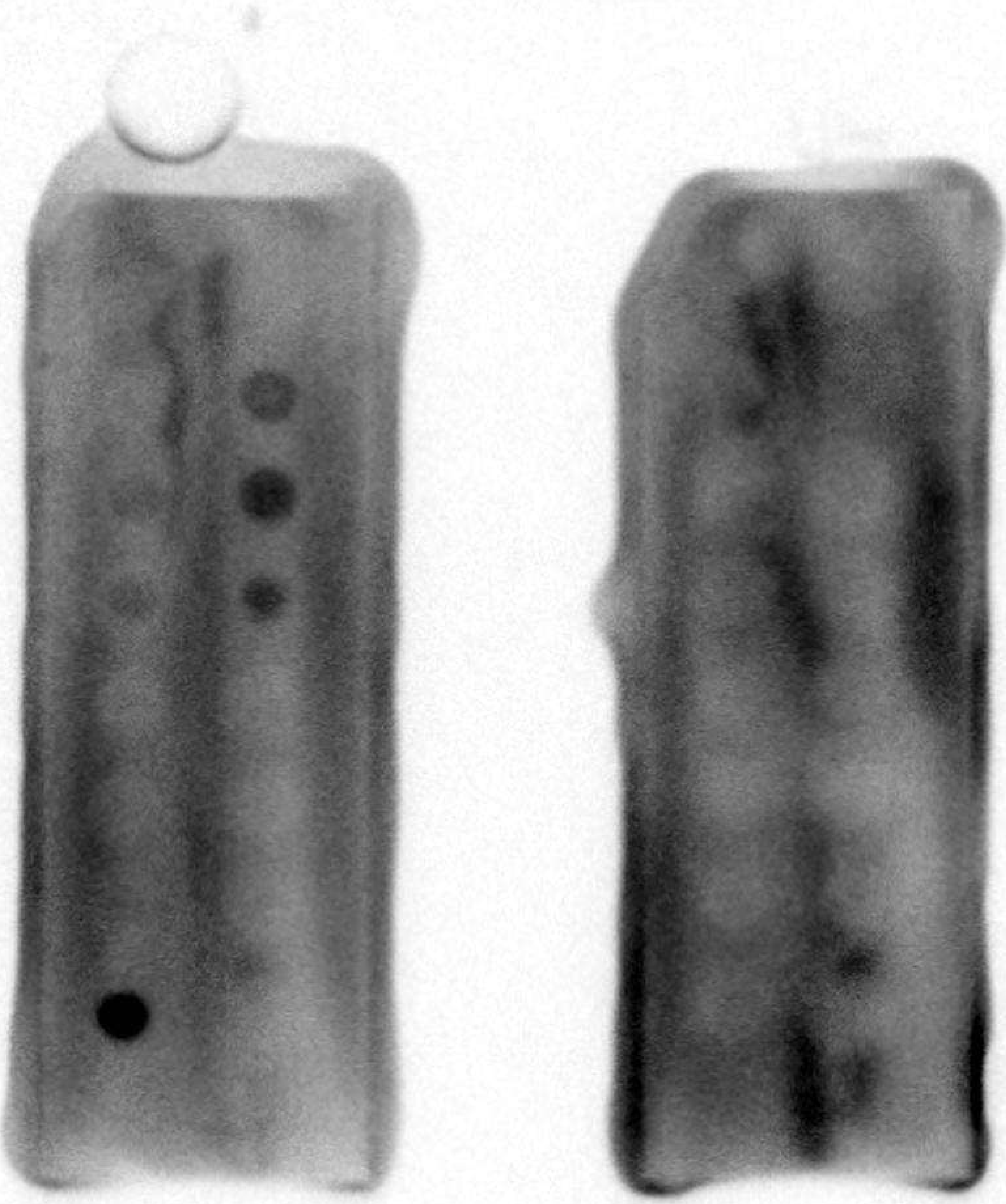

Chemiluminescence

Alpha-7.2 giardin (G194), Fig 3

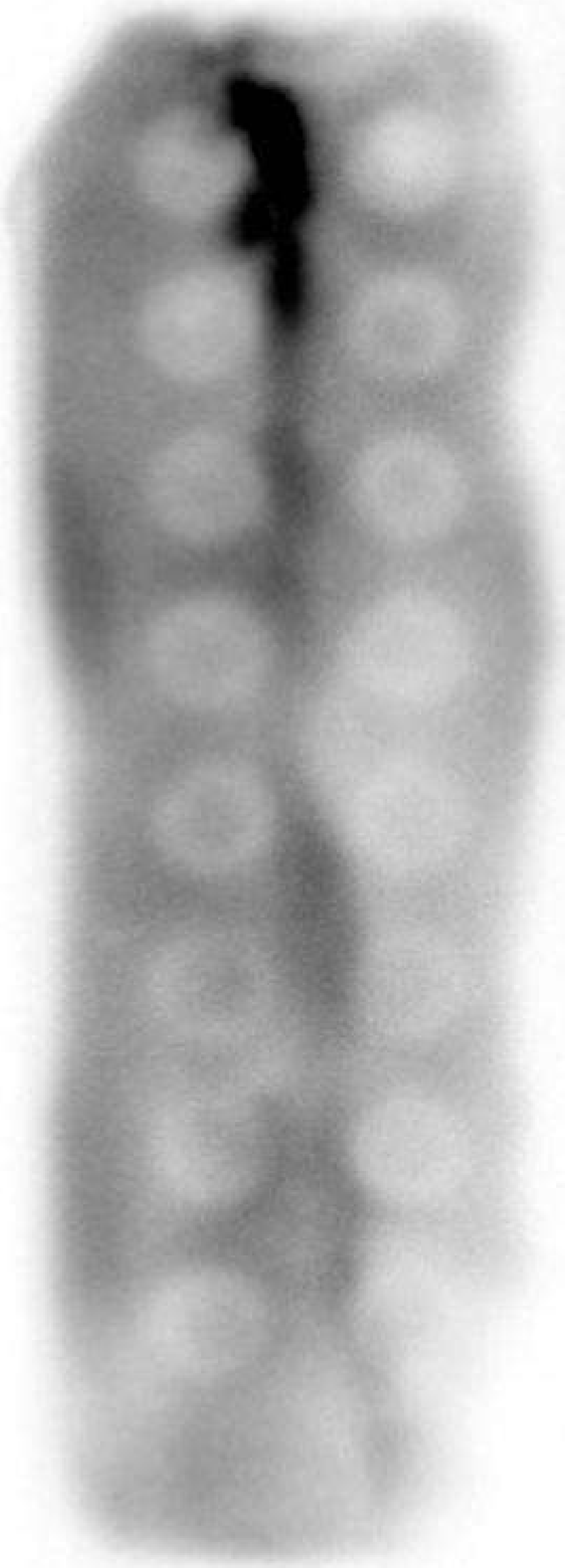

Chemiluminescence

Alpha-8 giardin (G196), Fig 3

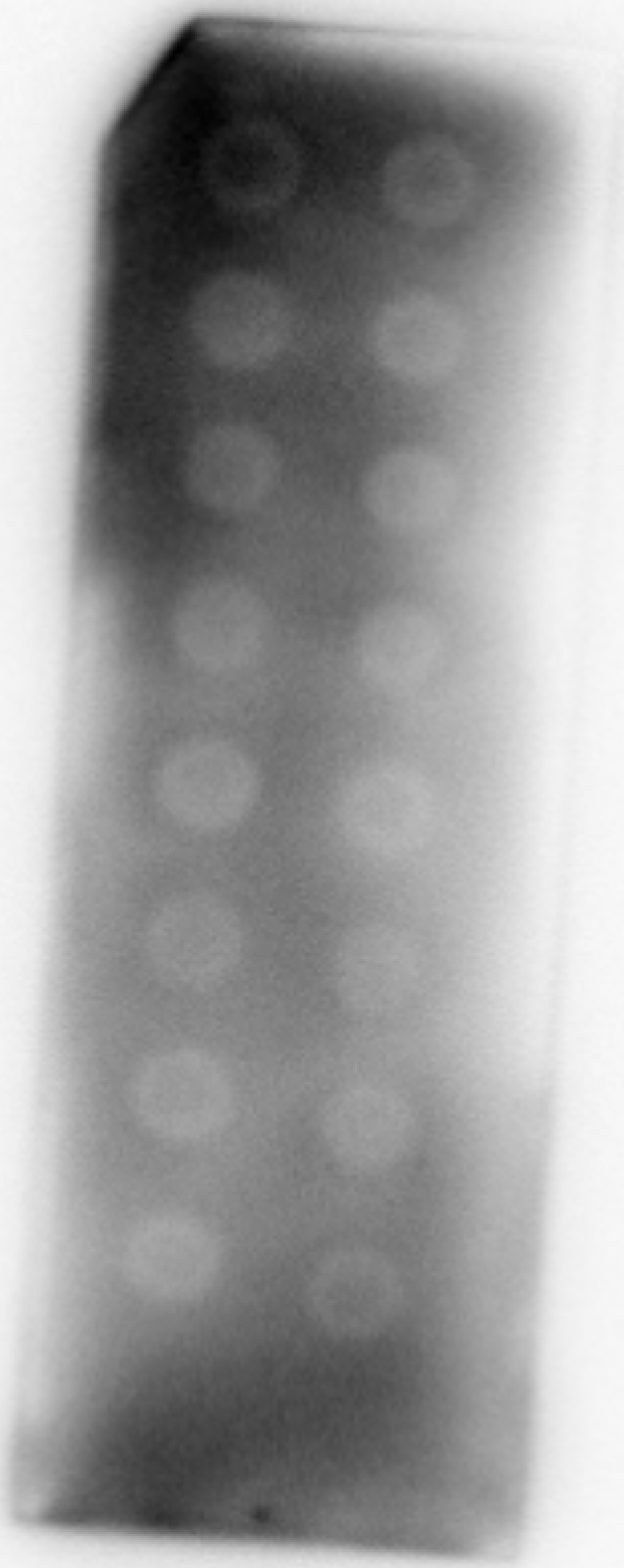

Chemiluminescence

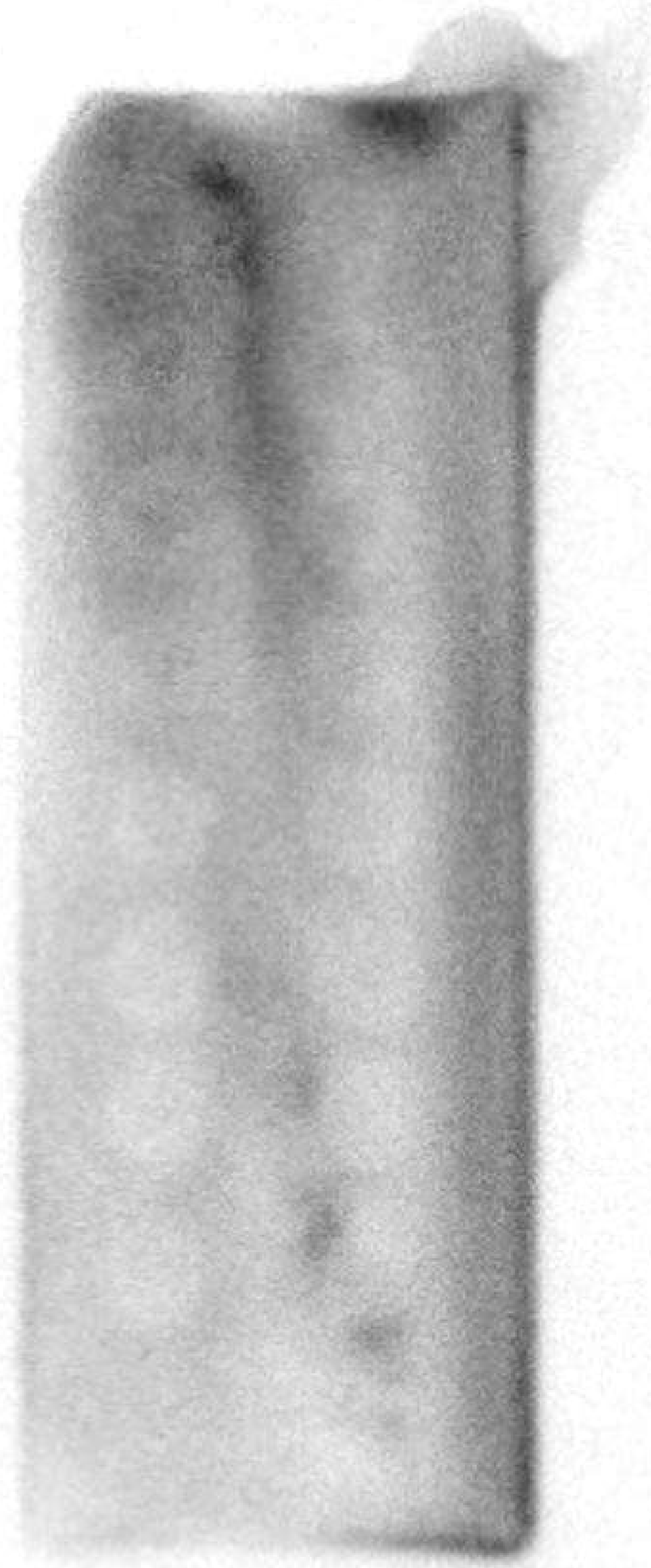

Alpha-13 giardin (G200), Fig 3

Chemiluminescence

Alpha-16 giardin (G203), Fig 3

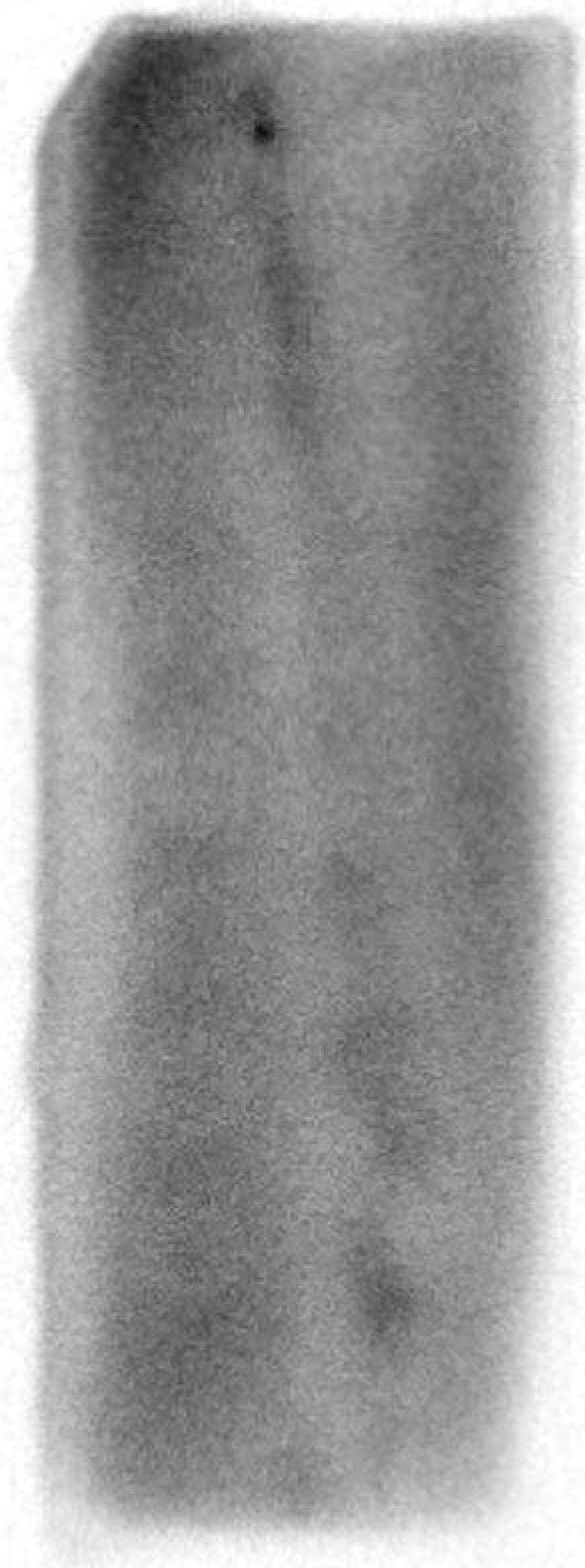

Chemiluminescence

Alpha-19 giardin (G206), Fig 3

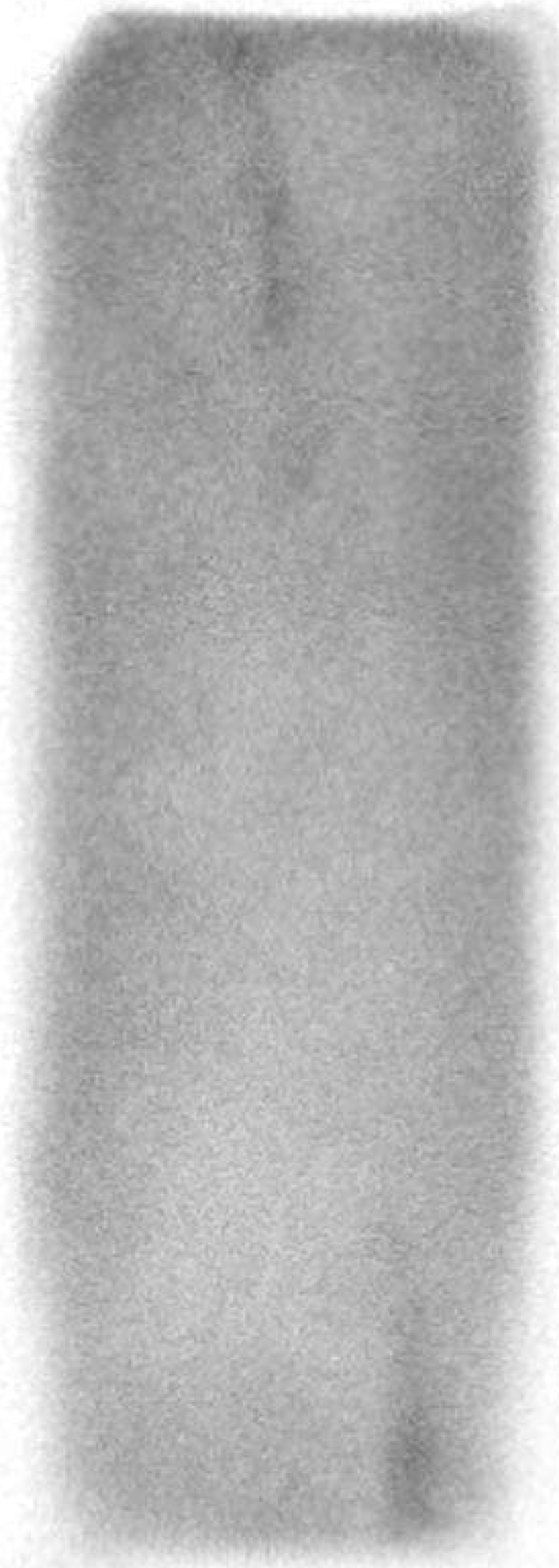

Chemiluminescence

Annexin A5 (G455), Fig 7

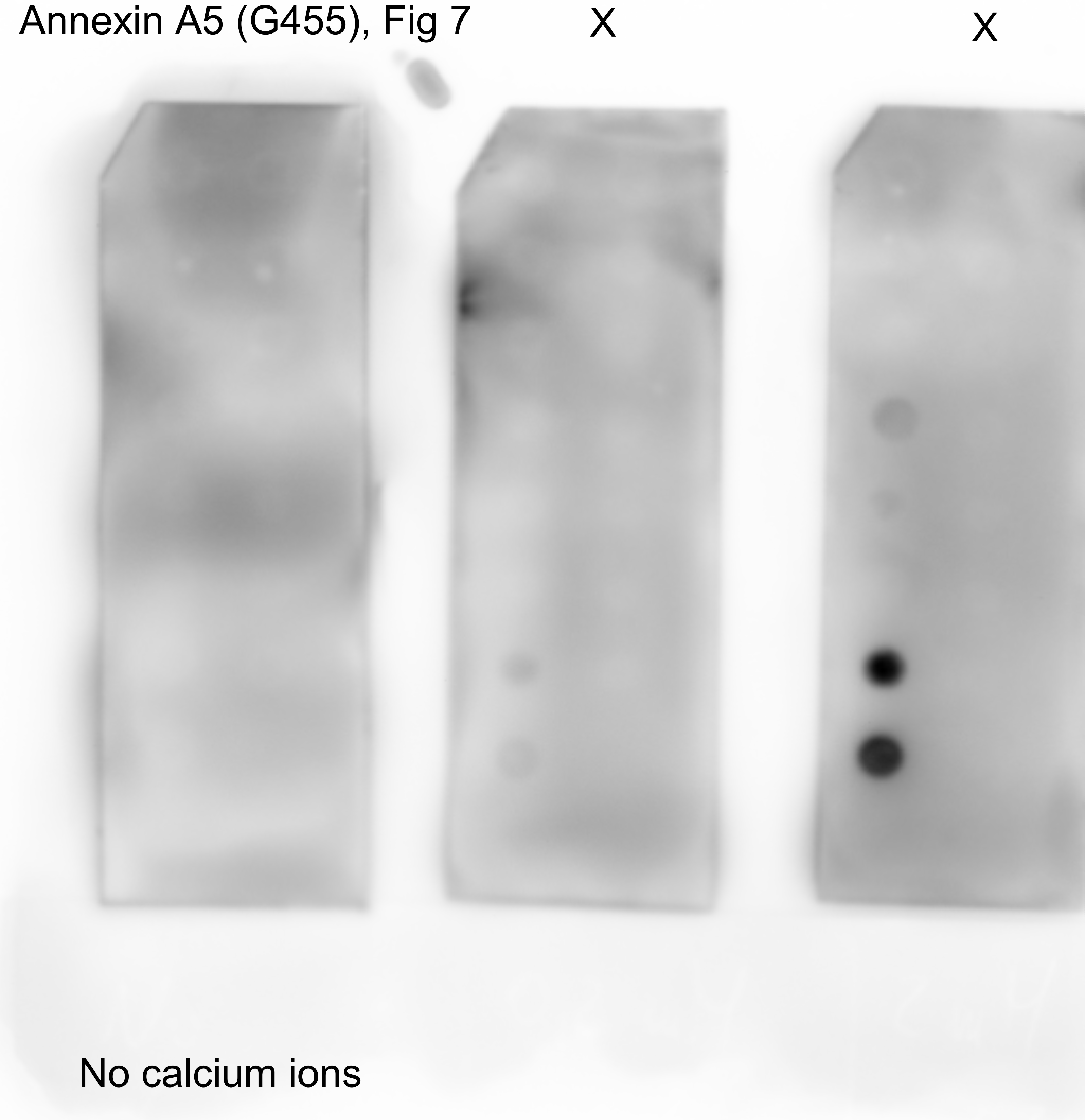

Chemiluminescence

Annexin A5 (G455), Fig 7

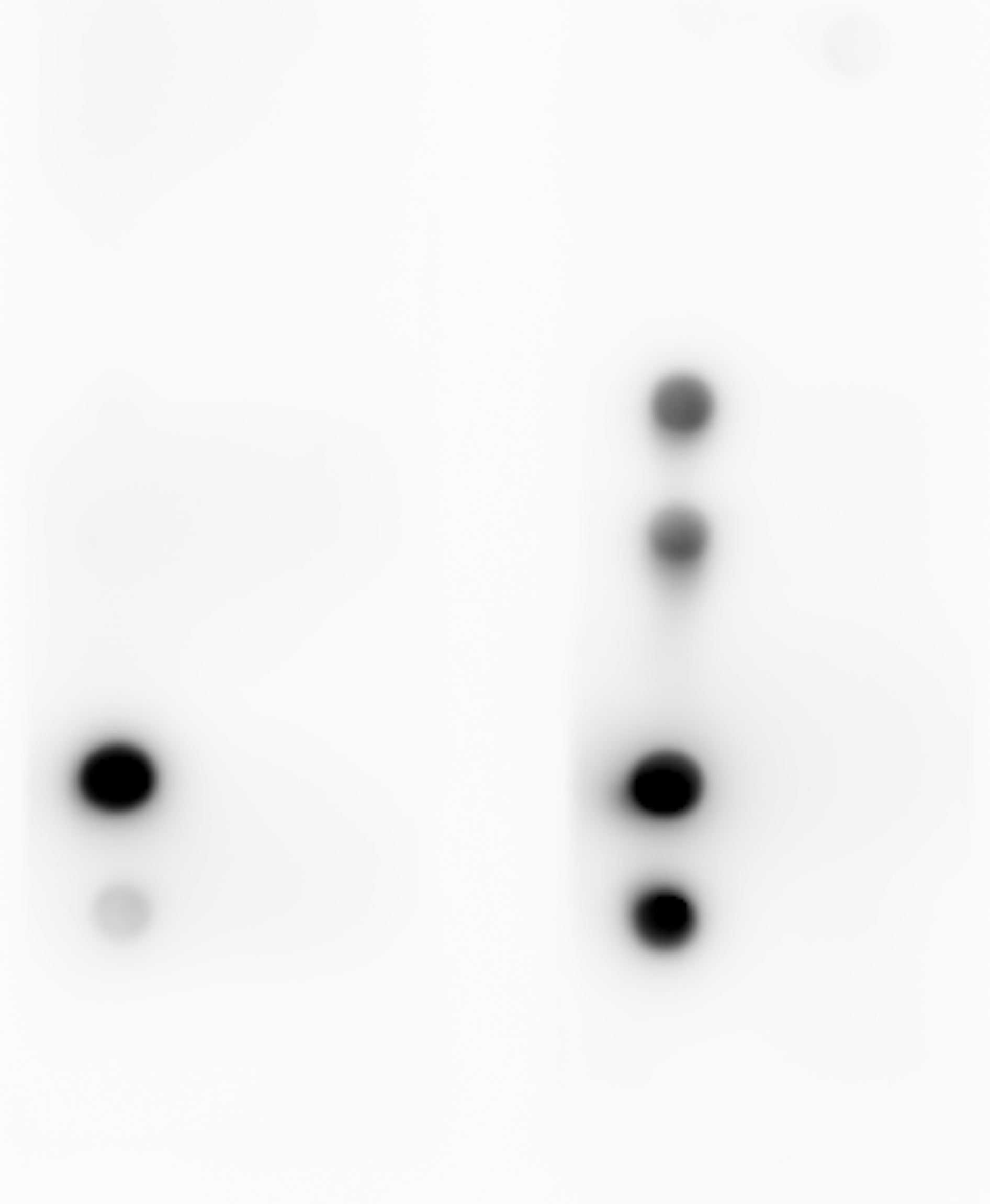

0.1 mM 1 mM

Calcium ions concentration

Chemiluminescence

Alpha-1 giardin (G25), Fig 6

ΔAlpha-1 giardin (G283), Fig 7

X

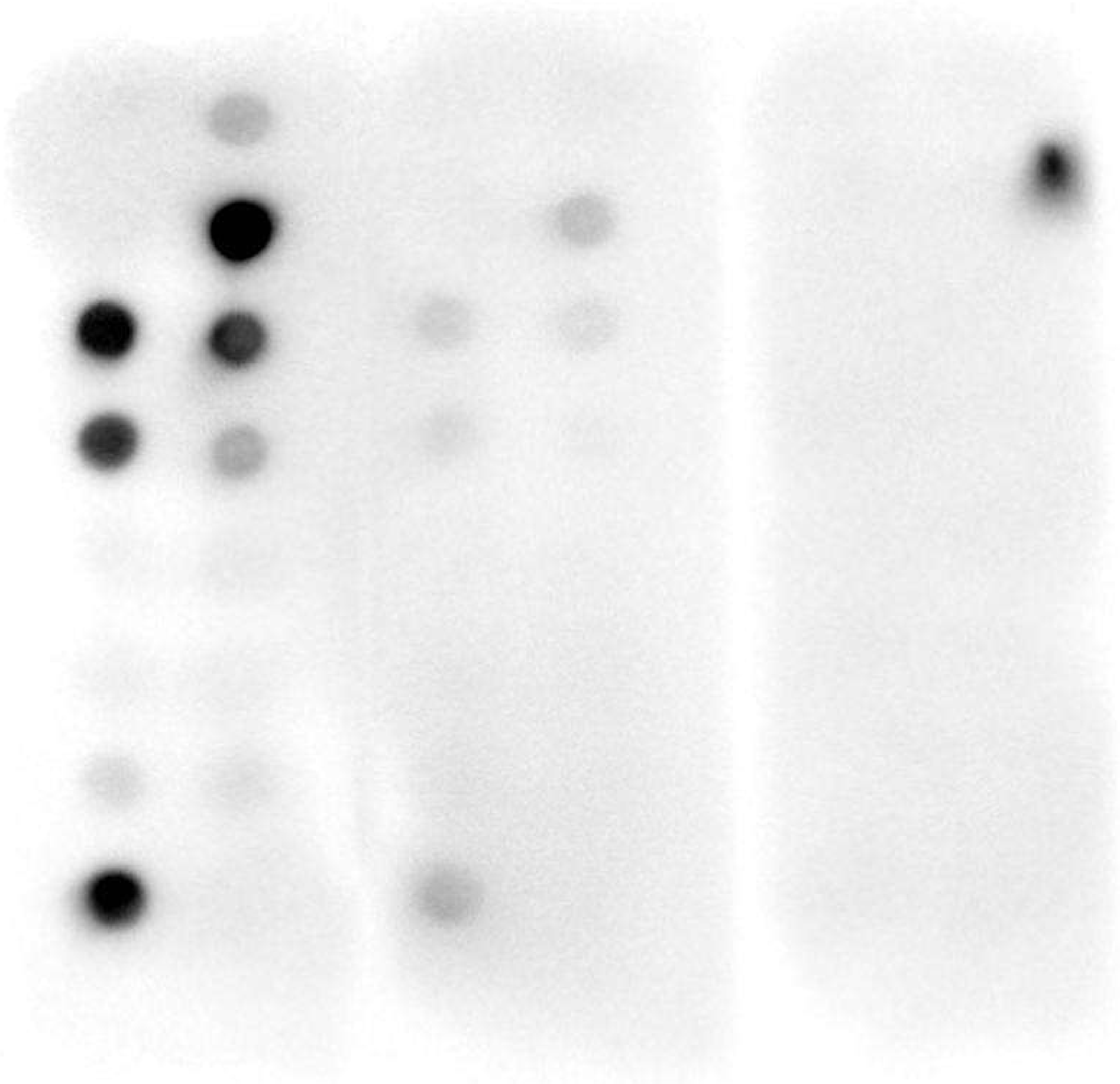

Chemiluminescence

Alpha-1 giardin produced in E. coli, Sup Fig 3

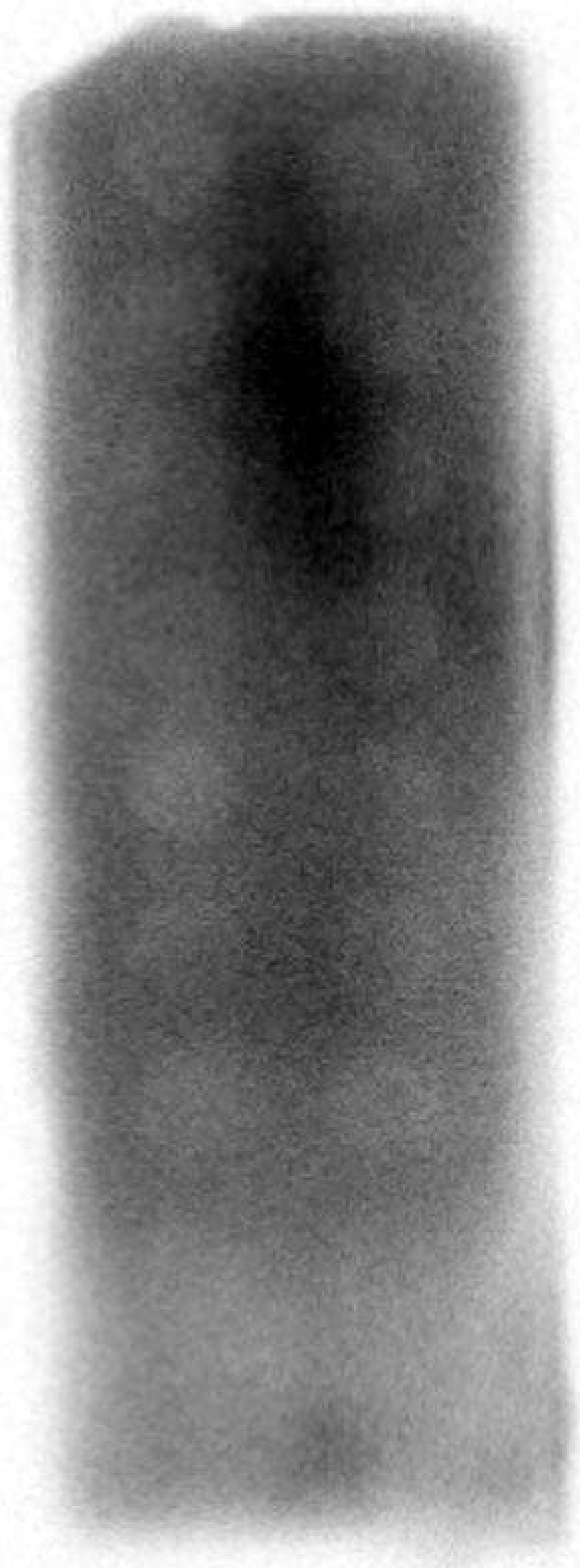

Chemiluminescence

Alpha-1 giardin produced in S. cerevisiae, Sup Fig 3

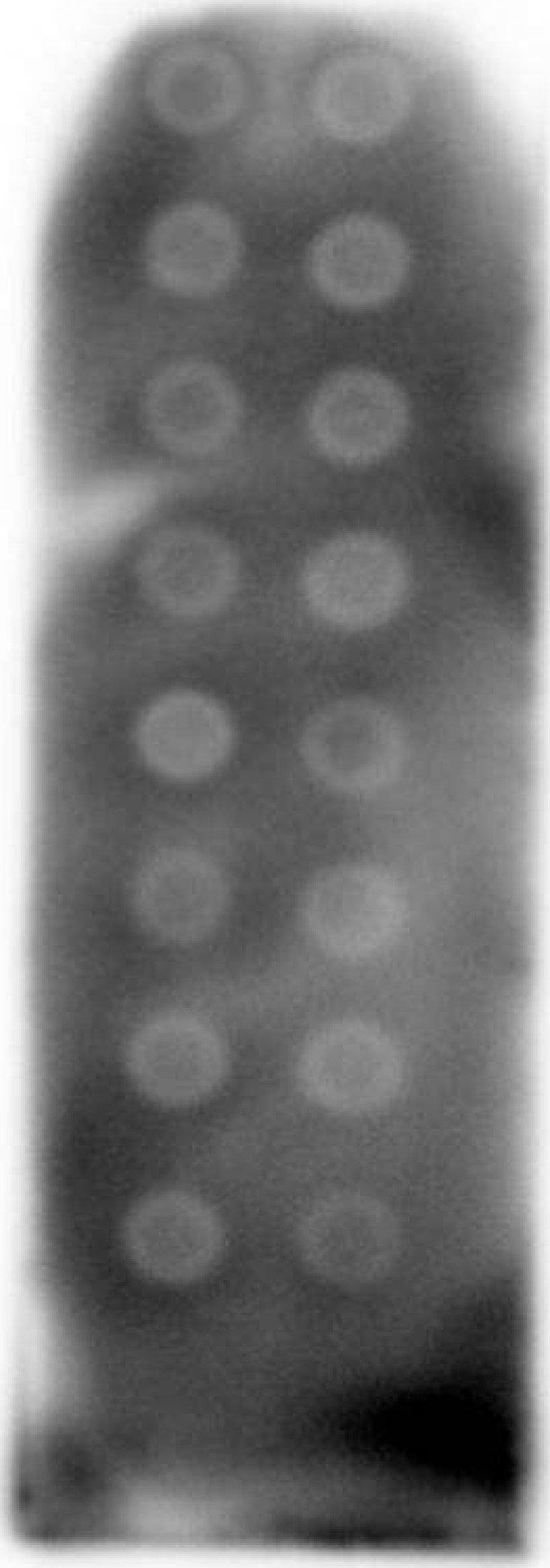

Chemiluminescence

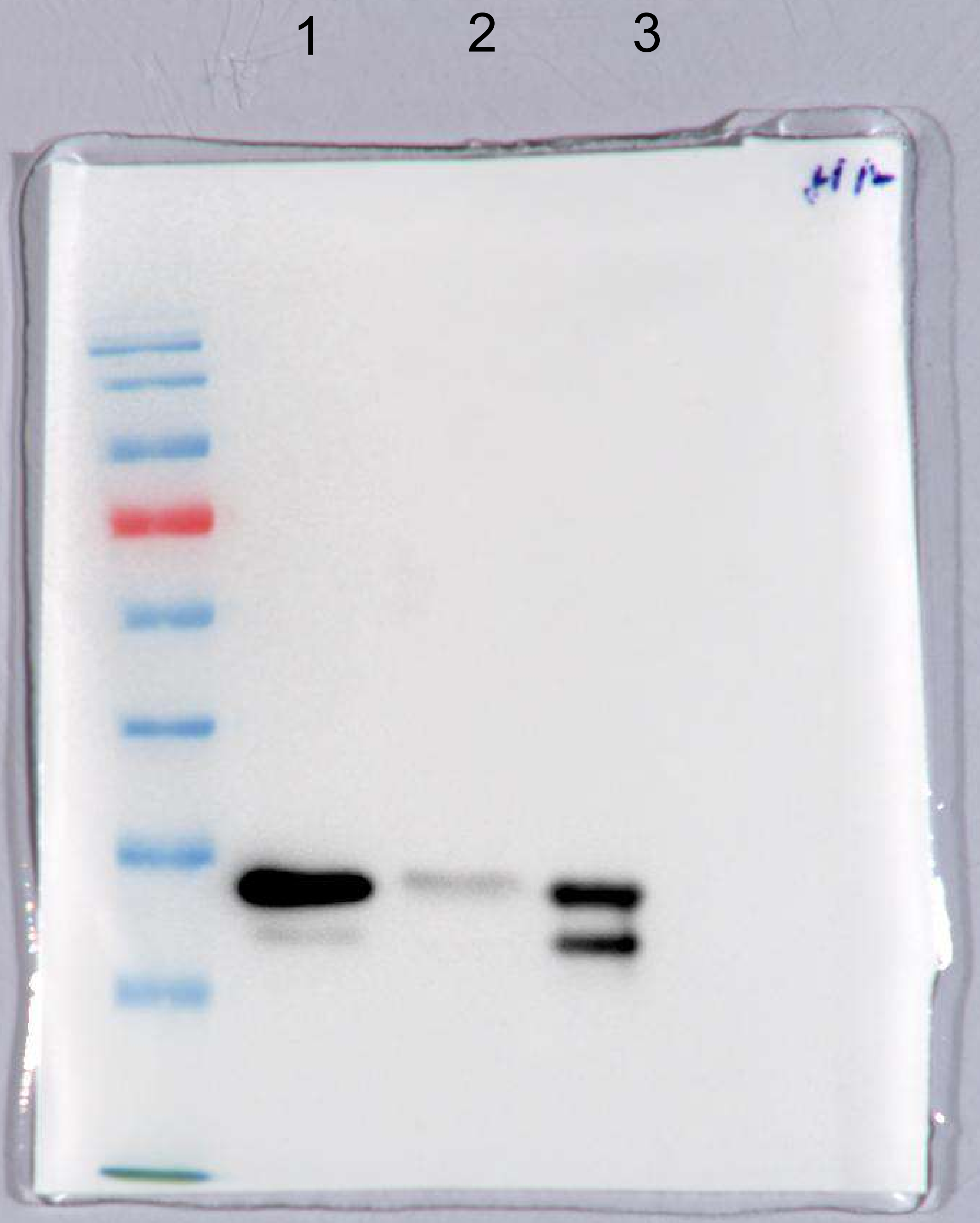

Alpha-1 giardin produced in: 1. G.lamblia 2. S. cerevisiae 3.E.coli, Sup Fig 3

Sample numbers correspond to the lane loading order

Chemiluminescence

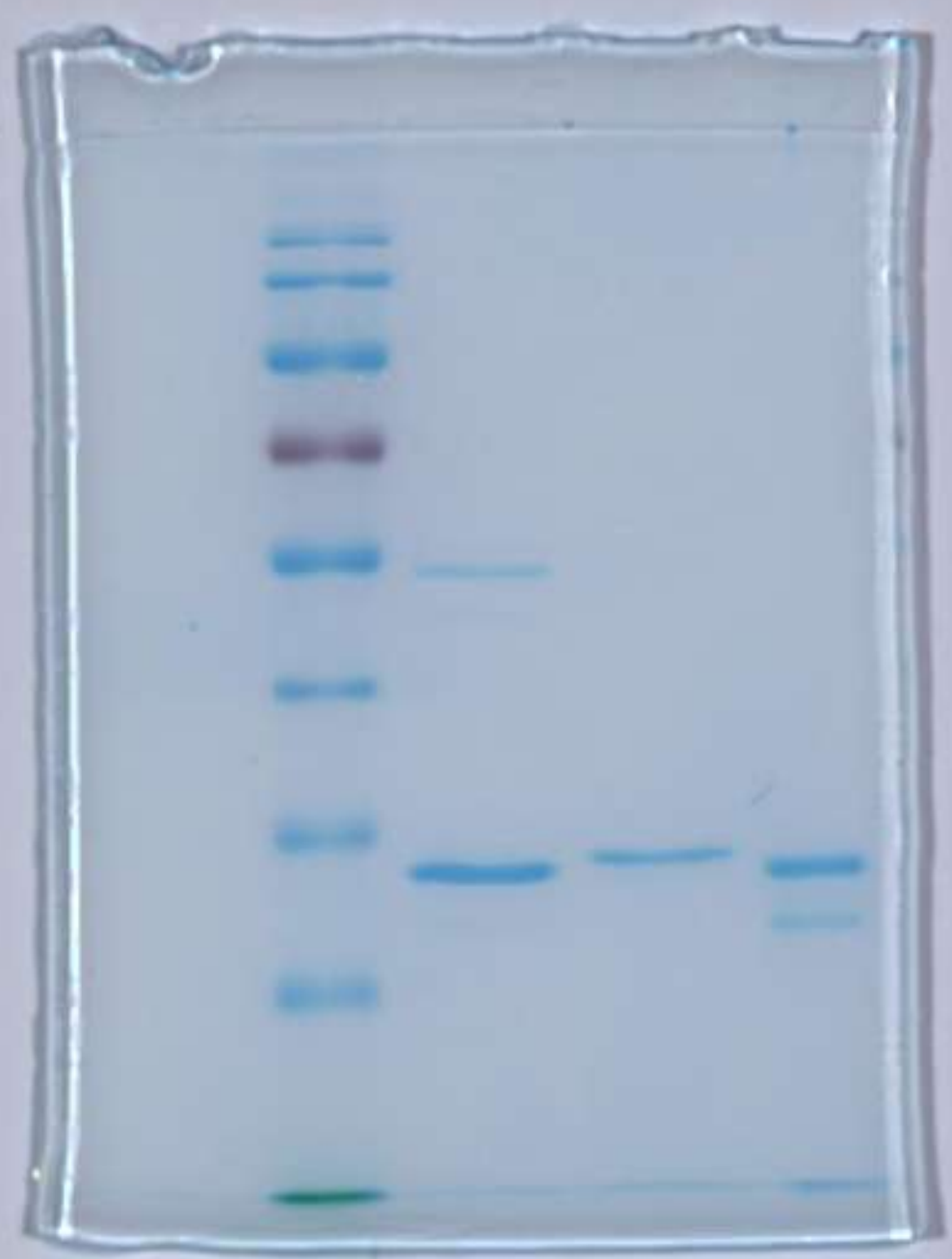

Alpha-1 giardin produced in: 1. G.lamblia 2. S. cerevisiae
3.E.coli, Sup Fig 3,
Sample numbers correspond to the lane loading order

White-light imaging

Alpha-1 giardin (G25), Sup Fig 3

V5-Alpha-1 giardin (G219), Fig 6

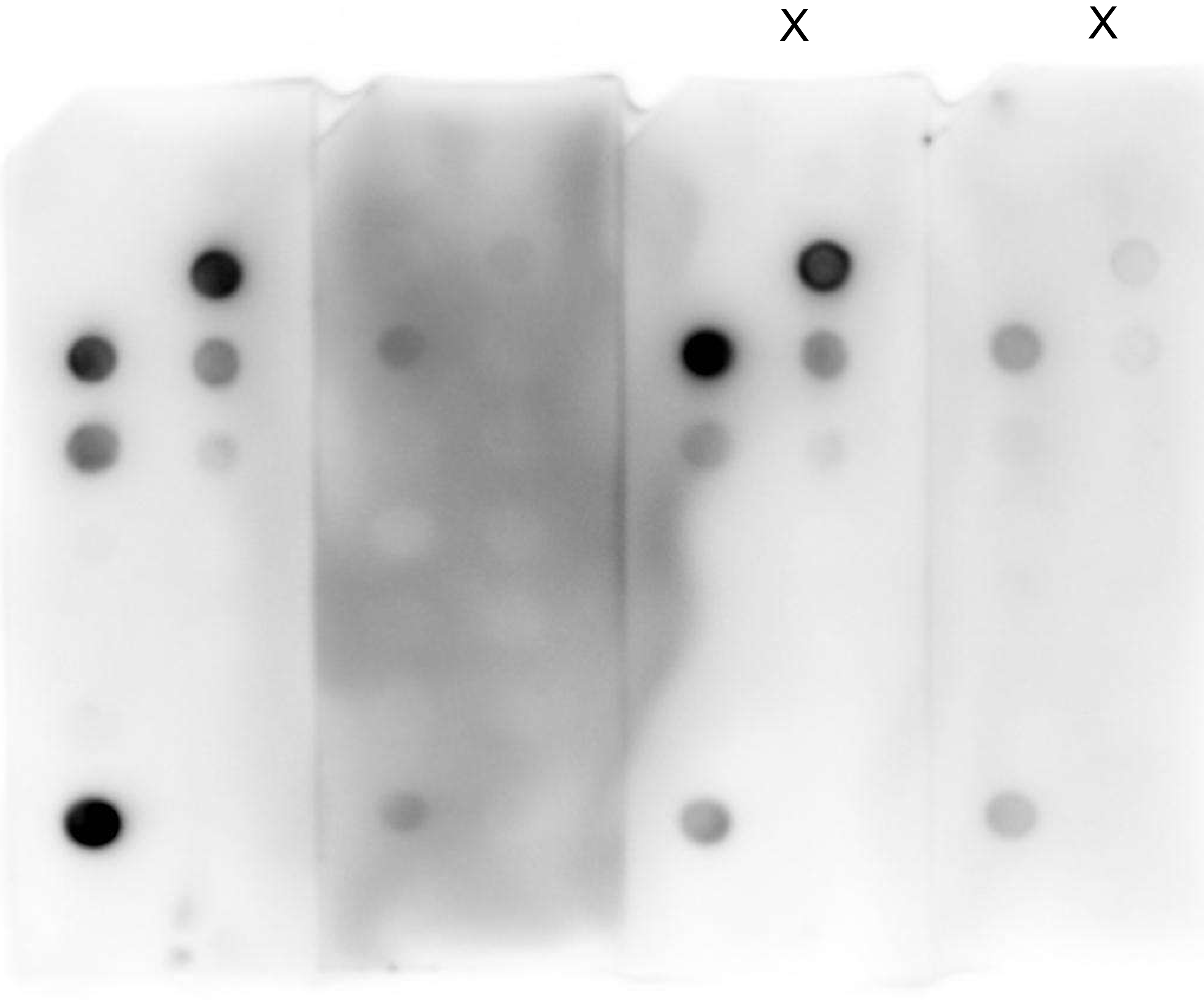

Chemiluminescence
