# Supplementary material for: The N-terminus of alpha-1 giardin, a parasitic annexin orthologue, is essential for oligomerization and lipid-binding activity
Source: PLoS Pathog. 2026 Oct 14;22(10):e1014407. doi: 10.1371/journal.ppat.1014407 (PMC13686171; doi:10.1371/journal.ppat.1014407)
Supplement: Supplementary file 2 [file ppat.1014407.s040.docx]

**Editorial Office**

**PLoS Pathogens**

Bern, 10. June 2026

**RE:** review manuscript PPATHOGENS-D-26-00541

Dear Dr. Pavel Dolezal,

On behalf of my colleagues Dawid Warmus, Corina Wirdnam, Tendai Muronzi, Erina A. Balmer, Manfred Heller and Özlem Tastan-Bishop, I am very pleased to submit a **revised** version of our manuscript **PPATHOGENS-D-26-00541** titled, “The N’ terminus of Alpha-1 Giardin, a parasitic annexin orthologue, is essential for oligomerization and lipid-binding activity”.

My colleagues and I have addressed all comments and provide both a tracked changes and clean version of the manuscript. We have added two extra supplementary figures and amended figure callouts. We have furthermore addressed requests from the editorial office, also included here.

We thank the reviewers for helping us improve our initial submission, we appreciate the care placed in reviewing this manuscript. We hope that you will consider this manuscript for acceptance, and we look forward to hearing from you in due course. If there is any further information that you require, please do not hesitate to contact me.

Kind regards,

Carmen Faso

**Rebuttal_Manuscript PPATHOGENS-D-26-00541**

**Journal Requirements:**

1) We ask that a manuscript source file is provided at Revision. Please upload your manuscript file as a .doc, .docx, .rtf or .tex. If you are providing a .tex file, please upload it under the item type u2018LaTeX Source Fileu2019 and leave your .pdf version as the item type u2018Manuscriptu2019.

*>> provided*

2) Please provide an Author Summary. This should appear in your manuscript between the Abstract (if applicable) and the Introduction, and should be 150-200 words long. The aim should be to make your findings accessible to a wide audience that includes both scientists and non-scientists. Sample summaries can be found on our website under Submission Guidelines:
<https://journals.plos.org/plospathogens/s/submission-guidelines#loc-parts-of-a-submission>

*>>provided*

3) Please upload all main figures as separate Figure files in .tif or .eps format. For more information about how to convert and format your figure files please see our guidelines: 
<https://journals.plos.org/plospathogens/s/figures>

*>> addressed*

4) We are unable to open the following Supporting Information file: Supplementary Data 8_plasmid sequences.zip. Please kindly revise as necessary and re-upload.

*>> We are not sure what the problem is here. We can correctly download and extract all sequence files from the zipped file linked straight from the submission. In any case, we have reuploaded these files and included their corresponding .APE versions for opening directly in any cloning management tool.*

5) Please amend your detailed Financial Disclosure statement. This is published with the article. It must therefore be completed in full sentences and contain the exact wording you wish to be published.
State what role the funders took in the study. If the funders had no role in your study, please state: "The funders had no role in study design, data collection and analysis, decision to publish, or preparation of the manuscript.".
If you did not receive any funding for this study, please simply state: u201cThe authors received no specific funding for this work.u201d

**Reviewers' Comments:**

Reviewer's Responses to Questions

**Part I - Summary**
Please use this section to discuss strengths/weaknesses of study, novelty/significance, general execution and scholarship.

Reviewer #1: While alpha-giardins were discovered 40 years ago and later identified as adherents and vaccine targets, their biochemistry and structure, which are the focus here, have never been characterized. Here excellent investigators use lipid-binding assays, cross-linking mass spectrometry, site-directed mutagenesis, confocal microscopy, and mass photometry to show the importance of a short N-terminal region of alpha1-giardin for unconventional protein expression. The figures are well-drawn; the methods, results, and discussion are well-written; and mass spec data deposited in PRIDE. The only weakness is the absence in the Introduction of the questions addressed in the paper.

*>> Thank you for the appreciation! As another reviewer noted, the last third of our introduction was not included in the pdf (we have no idea how that can even happen). The complete introduction, clearly stating the questions we address in this work, is now included in the revised version of the manuscript and shown below:*

“*At membranes, annexins can form various structures (Figure 1), ranging from simple aggregates to complex networks, depending on the specific annexin and membrane conditions ^8,23–25^. The specific mechanism of membrane binding varies among different annexins and is influenced by factors such as membrane lipid composition, presence of Ca^2+^ ions and pH ^1,4,26,27^. Following membrane binding, annexins have been speculated to insert themselves into the target membrane ^1,14,28^. This process is thought to involve a conformational change in annexin structure, leading to lipid bilayer destabilization.*

*Annexins from the intestinal protist parasite Giardia lamblia (syn. intestinalis, duodenalis) are called alpha-giardins (αGs) ^1,2,29–36^.Two members of the 21-strong protein family, alpha 1-giardin and alpha 11-giardin (α1G and α11G) were previously identified as putative UPS substrates following their detection in the surface proteome ^37^ and secretome ^38,39^ of axenic G. lamblia cultures. Both proteins carry no bona fide signal sequence for secretion ^37–39^. A recent investigation localized α1G and α11G to the parasite’s peripheral endocytic compartments ^19^ (PECs, formerly known as peripheral vacuoles), single membrane-bound endo-lysosomal organelles ^40–43^, previously implicated in protein release ^19,44,45^. Furthermore, PECs-associated α1G and α11G participate in a protein complex which includes other UPS substrates, some of them recognized virulence factors in G. lamblia ^19^. Importantly, α1G is currently considered one of the strongest candidates for vaccine development for giardiasis ^34,37,46,47^, a worldwide water-borne diarrheal disease widespread in areas where water sanitation is problematic and safe drinking water cannot be guaranteed.*

*In this report, we present a broad functional characterization of αGs in terms of their subcellular location in the G. lamblia system, their lipid-binding behaviour, and their protein interactomes and predicted configurations using XL-MS ^48,49^. Given its importance as a vaccine candidate for giardiasis and its implication in virulence-related UPS mechanisms in G. lamblia, we narrow our focus to α1G. Using site-directed mutagenesis coupled to confocal microscopy and mass photometry (MP), we dissect the contribution of the short (less than five residues) N-terminal region of α1G and investigate oligomeric forms of both wild type (wt) and mutant α1G variants in solution. These data are then used to inform simulations of molecular dynamics of α1G variants, to predict the behaviour of α1G variants at PEC membranes in terms of oligomeric stability and occupancy, and to develop a new working model for α1G function at PEC membranes.”*

Reviewer #2: This manuscript presents a comprehensive analysis of α-giardins, with a particular focus on α1-giardin and its role in lipid binding, oligomerization, and subcellular localization. The study integrates multiple complementary approaches (confocal microscopy, co-IP, XL-MS, mass photometry, and molecular dynamics), which represents a clear strength.

Although the work is thorough, technically sound, and well presented, with a robust experimental design, it primarily constitutes a further characterization of α1-giardin rather than providing a fundamentally novel conceptual advance (for example, in terms of functional insight).

*>> we see the reviewer’s point and indeed, our focus is primarily on α1G, as also shown in our choice of title. However, we provide evidence for the essentiality of the N terminus of α1G while also presenting important insight into the behaviour of other alpha-giardins. Furthermore, we biochemically distinguish between different members of this protein family while using mutagenesis and molecular simulation data to define a possible mechanistic sequence leading to recruitment to PECs-PVs.*

Reviewer #3: Warmus et al have studied the localization, lipid binding and oligomerization of alpha-giardins (aGs), mainly alpha-1 giardin (a1G). Several aGs localize to peripheral endocytic compartments (PECs) in Giardia, including a1G. The N terminal region of a1G is important for lipidbinding and oligomerization. A model for the role of a1G and other PEC related aGs is suggested. It is a paper with many interesting results but the presentation can be improved. I have the following comments:

**Part II – Major Issues: Key Experiments Required for Acceptance**
Please use this section to detail the key new experiments or modifications of existing experiments that should be absolutely required to validate study conclusions.

Generally, there should be no more than 3 such required experiments or major modifications for a "Major Revision" recommendation. If more than 3 experiments are necessary to validate the study conclusions, then you are encouraged to recommend "Reject".

Reviewer #1: None.

Reviewer #2: 1. Terminology: PVs vs PECs
The term “peripheral endocytic compartments (PECs)” is problematic because it is not widely adopted in the field. Instead, the consensus terminology in recent literature—including primary research articles and reviews from the last five years—is “peripheral vacuoles (PVs)”, reflecting their well-established identity as endo-lysosomal organelles with multifunctional roles.
For example, recent studies consistently describe these structures as PVs and highlight their:
-localization beneath the plasma membrane,
-role in endocytosis, digestion, acidification, and
-functional equivalence to endosomal/lysosomal compartments since it also contains lysosomal proteins (e.g., cathepsins and RNase).
Furthermore, contemporary literature acknowledges the use of dual terminology but still prioritizes PVs. For instance, PVs are described as “peripheral vacuoles/peripheral endocytic compartments (PVs/…)”, explicitly indicating that PVs are the primary accepted term.
Importantly, recent reviews and system-level analyses of Giardia cell biology also consistently use PVs as standard nomenclature when describing trophozoite organization and subcellular compartments.
Taken together, these references support the view that:
PVs are not merely endocytic compartments, but rather hybrid endo-lysosomal organelles with additional MVB-like properties, as widely recognized in the field.
Recommendation: The authors should adopt the term “peripheral vacuoles (PVs)” throughout the manuscript.

*>> We agree with the reviewer that the term “peripheral vacuoles (PVs)” is historically widely used in the literature. However, the use of the term “vacuole” is not substantiated by the data generated so far, neither in terms of function nor in terms of molecular machinery associated to these organelles. There are no obvious correlations, for example, between Giardia’s organelles and plant/yeast vacuole, nor are there any parallels or similarities with parasitophorous vacuoles, as seen in Apicomplexa.*

*The obvious function for these organelles is uptake from the extracellular space, with growing evidence for a role in release too. For this reason, and given all data concerning their molecular complement, in Santos et al., 2022 we proposed the renaming of these organelles to peripheral endocytic compartments, since this is essentially the most faithful description of their function. However, since this might cause confusion while we transition into use of the new term, we agree to add PV to every mention of PEC in this manuscript.*

2. Variability in expression levels
The manuscript reports variable expression levels for the same giardin constructs (e.g., Fig. 2), but no explanation is provided. This raises several concerns:
• Which vectors/promoters were used for each construct?

*>> we take the reviewer’s point and have prepared a new supplementary figure 1 showing the vector backbones we used for cloning. Furthermore, we provide all construct sequences in both a fasta and .ape format in supplementary data 8.*

• Are these episomal or integrated constructs?
• Could expression variability influence localization or biochemical assays?

*>>All our lines are episomal transfectants, we state this in materials and methods (line 371 of the revised manuscript) and in the main body of the revised manuscript (line 113). Since expression variability can indeed affect readouts, we specifically chose to work with lines which present comparable expression levels, which unfortunately meant abandoning others which did not.*

Additionally, the rationale for performing immunoprecipitation before lipid strip assays (Fig. 3) should be clarified. Is this due to low expression levels or to enriched complexes?

*>> As we explain in the manuscript and show in the revised supplementary figure 3 (formerly supp figure 1) our efforts to express alpha 1-giardin in either E. coli or baker’s yeast, were not successful. This means our only current source of alpha 1-giardin are transgenic Giardia cells expressing an HA-tagged variant for this protein.*

*To obtain enough purified alpha 1-giardin to interrogate lipid binding behaviour specifically, without any other component, we have to IP to isolate and enrich for alpha 1-giardin.*

3. Inconsistent use of constructs
The manuscript states that G25 and G282 are essentially equivalent constructs expressing α1G, differing only minimally (page ~14). However:
• G25 is used in some experiments (e.g., Fig. 6),
• G282 in others (e.g., Fig. 7).
A clear justification is required for this inconsistency. If constructs are interchangeable, consistency should be maintained.

*>>Due to the cloning strategy, the 5' UTR in the construct used to generate line G283 was not absolutely identical to that of the corresponding construct used for line G25. We felt that this could lead to small expression differences that would affect the interpretation of results. Therefore, we created G282 as an additional control. Whenever we analyzed G283 by microscopy we employed G282 as the most accurate control. For experiments involving purified proteins, G25 and G282 are identical.*

4. Lack of experimental validation of oligomerization
A central claim of the manuscript is the oligomerization of α1G. However:
• This is primarily inferred from mass photometry and XL-MS, not directly demonstrated.
• No biochemical validation is provided.
The authors should strengthen this claim by:
• performing native (non-denaturing) Western blot,
• or complementary biochemical approaches.
Additionally, since G25 is used in several assays, it would be informative to specifically test the oligomerization of this construct.

*>> We take the reviewer’s point that the experimental validation of oligomerization could be strengthened. Since functional i.e. lipid-binding alpha1-giardin cannot be expressed in E. coli and baker’s yeast, we do not have access to large amounts of isolated protein. This is necessary for biochemical validation intended as a direct test of isolated protein behavior.*

*We have attempted to run native Western blots, but, even though we always observed differences between the native and the mutated giardin, we did not feel we had the situation completely under control. for example, we cannot claim exactly which band is a trimer and whether the higher bands contain pure giardin or a mixture of interacting partners. Therefore, we do not want to show such data in the main paper.*

*An example of such a gel is shown here:*

5. Overinterpretation of oligomerization
The manuscript repeatedly links oligomerization with function and PEC association (e.g., lines 271–272), yet:
• no direct functional assays demonstrate this relationship,
• oligomerization itself is not experimentally validated beyond indirect evidence.
The statement:
“MP and XL-MS data do point towards a major role for oligomerization in functionality and PECs association.”
is therefore an overstatement and should be toned down.

*>> We take the reviewer’s point. However, analysis of mutant alpha1-Giardin variants such as the V5-tagged version and the one lacking the first 3 N terminal residues demonstrate that functionality because of these manipulations is severely affected. We show these data and report on subcellular localization, lipid-binding behaviour and oligomer formation as detected using mass photometry.*

6. Title overstatement
The title claims:
“...is essential for oligomerization and lipid-binding activity”
However:
• oligomerization is not directly demonstrated experimentally,
• its causal relationship with lipid binding is inferred.
The title should be revised to reflect the level of evidence presented.

*>> We take the reviewer’s point. However, we provide orthogonal data showing that the elimination of three residues which constitute the (extremely short) N terminus of alpha 1-Giardin, as defined by the consensus on the location of the annexin fold domains, causes the protein to lose binding affinity to specific lipids and loss of higher order structure. This means that these residues are essential to ensure correct binding and higher order structure formation. We therefore stand by the title of this work which summarizes this observation.*

7. Calcium dependence interpretation
In the Discussion (lines ~281–294), the authors state that α1G lipid binding is not affected by calcium, in contrast to annexin A5.
However, the cited reference (Saroja et al., 2022) indicates:
• α1-giardin disengages from membranes at high calcium concentrations, potentially due to dimer formation.
This appears contradictory. The authors should:
• clarify this discrepancy,
• or revise the interpretation accordingly.

*>> We do not fully understand this point. We do not provide data on alpha 1-Giardin’s dependance on calcium ions for binding. We therefore do not contradict any previously published data.*

8. Comparison with annexin A5
The conclusion emphasizes similarities between α1G and annexin A5. However:
• the data actually highlight significant differences (e.g., calcium dependence, behavior).
• Therefore, the statement:
“highlighting unique characteristics of α1G... and showing how G. lamblia can be a useful heterologous model...”
is not fully supported and should be reconsidered or softened.

*>> We take the reviewer’s point. We wish to underline that expression of a non-Giardia protein in Giardia rarely results in an outcome that is consistent with the native protein’s behaviour. However, given the obvious differences between these proteins, we agree to tone down our statement, as shown in the revised version of the manuscript (lines 356-7*

Reviewer #3: (No Response)

**Part III – Minor Issues: Editorial and Data Presentation Modifications**
Please use this section for editorial suggestions as well as relatively minor modifications of existing data that would enhance clarity.

Reviewer #1: none.

Reviewer #2: 9. Insufficient description of expression constructs and vectors
Although vector sequences are provided in FASTA format, it is difficult to evaluate the experimental system. Critical information is missing, including vector backbone, promoters, and selection markers. Either a schematic representation or a detailed description is necessary.

*>> We fully agree with this comment and now provide schematics of all construct categories in a new supplementary figure 1. We have corrected figure callouts to account for this and other additions.*

10. Inconsistency in co-IP results
It is unclear why α6-giardin is not recovered in its own top 10 pull-down. This should be explained.

*>> We agree with this comment and have generated a new version of this figure which is now correct and hopefully improved.*

Reviewer #3: Line 51. The Introduction needs serious re-writing and extension. To me it does not look like an Introduction of a paper, it is shorter than the abstract and Giardia and alpha-giardins are not even mentioned! Here it is important to explain for the reader what Giardia is, why is it important to study. What is already known about alpha-giardins and specially a1G when it comes to disease.

*>> For reasons we do not understand, during conversion to a pdf, the last section of the introduction was cut. This is now corrected and addresses the reviewer’s concerns.*

Line 60. Remove Fig. 1, it is not needed, annexins can be discussed without a figure.

*>> Given the introduction has now been fully restored, we still think figure 1 is important and would like to include it.*

Line 94. “In Giardia trophozoites”, other cell stages are not studied.

*>> addressed*

Line 96. Is the gene driven by promoter of each alpha-giardin and is it episomal or integrated constructs? Important info.

*>> addressed with new supplementary figure 1 and clarified in the revised manuscript (lines 355-6).*

Line 101. A C terminal epitope HA-tagging approach is used here to localize the alpha-giardins. Many alpha-giardins have been localized before with epitope tags of different kinds and with specific antibodies. The results in this study is in line with some of that published data but not all.

I suggest to make a table with the localization in this paper and a comparison with all earlier published localizations to get a more clear picture of the localizations.

It should be noted that certain alpha-giardins have C terminal extensions: The sizes of the C-terminal extensions are 17 (α8G), 26 (α14G), and 77 amino acids (α19G).
By using polyclonal serum, α8G was located on the plasma membrane and flagella, not cytoplasm as here. See doi: 10.1016/j.exppara.2010.05.028. ,

*>> We take the reviewer’s point. Expression of an epitope-tagged α8G variant was consistently localized to the cytosol. We detect routinely with a monoclonal anti-HA antibody (not a polyclonal serum) which has shown extreme robustness as a detection tool across several years and publications from various groups.*

a14G binds lipid strips as seen in this paper doi: 10.1128/mSphere.00032-15. and it adheres to microtubules of the flagella via self-assembly that may regulated by Ser/Thr-phosphorylation. doi: 10.1007/s00436-007-0758-6. doi: 10.1016/j.jmb.2008.11.012.

alpha19-giardin, carries also an N-terminal sequence extension of MGCXXS, alpha19-giardin exclusively localizes to the ventral pair of the giardial flagella. A mutant in which the putatively myristoylated N-terminal glycine residue was replaced by alanine lost this specific localization. It has been suggested that the dual lipidation of alpha19-giardin is responsible for its specific flagellar localization. Has membrane association. doi: 10.1128/EC.00136-09.
Thus, the localization in this paper does not fit with earlier data and one should be careful with the cytoplasmic type of localization seen with the alpha-giardins with C terminal extension like a8G and a19G. There is a risk that the HA tag causes mislocalization to the cytoplasm and then the correct modifications and interaction with cellular partners is not seen either, see below.

*>> We take the reviewer’s point. We note that in Weiland 2005 (Annexin-like alpha giardins: a new cytoskeletal gene family in Giardia lamblia*) a tag was used at the C terminus (AU-1) and this may have also impacted the data shown in this publication. We acknowledge that differences will always be there, given tools and approaches are different. For example, compared to what was done in Einarsson et al., 2016 (doi: 10.1128/mSphere.00032-15), we do not have a GST tag at the N terminus, which might also affect behaviour. We agree that the presence of C terminal extensions in certain members of this protein family should be more carefully examined. However, we also localize and report on alpha 19-Giardin at flagella, as previously published in doi : 10.1128/EC.00136-09, so there is no discrepancy on these data.*

*In our hands and with our constructs, alpha 14-giardin expression levels were too low to make any conclusion as to its subcellular localization. For the same reason, we did not test it on lipid strips. We therefore do not contradict previous reports.*

Line 105 and 106. Use numbering in the right order. Do the same through-out the paper, see also line 118.

*>> addressed and corrected*

Line 113. Was the lipid binding in Fig. 3 dependent on the calcium level?

>> no, Calcium ion supplementation was not required for binding. All data for lipid binding, unless stated as in the case of anxA5, was generated in the absence of calcium ion addition. We state this in line 130 of the revised manuscript and in the materials and methods section, lines 458-60.

Fig. 3B is not refered to as far as I can see.

>> we thank the reviewer for spotting this omission. We have now incorporated the following paragraph in the revised version of the manuscript, lines 134-6:

*“α1G affinity for PIP residues was further analyzed using a PIP array (Figure 3B), highlighting PI(3)P, PI(5)P and PI(3,5)P as preferred targets for binding, in these experimental conditions.”*

The proteomics data show that many proteins co-purify with the tagged alpha-giardins. How do you know that it is the tagged alpha- giardin that bind lipids and not the interaction partner?

*>> This is an important point and we thank the reviewer for raising it. We have done so too in our discussion.* While we cannot exclude the possibility, we strongly suspect giardins to be responsible for binding due to the following:

-	*The co-IP results for lines G282 vs. G283 show that all interaction partners except for one are maintained, the only other difference being the mutant alpha giardin.*

-	The interacting partners are present at low concentrations in the IP eluate, making unlikely the strong and consistent detection that we observe.

Line 115+116. This does not fit with earlier data for a8G.

*>> this comment was addressed in a previous section.*

Line 117. Explain what the numbers in paranthesis are.

*>> we realize the use of G for giardin and G for Giardia line can be unclear. We have corrected by adding the term “line” prior to every reference to a transgenic line.*

Line 121. There must be a mistake in Fig. 4B, it is the same data for a6 and a11. Add the orf numbers of the protein, this does not say anything since there are so many variants of some of the proteins.

*>> We agree with this comment and have generated a new version of this figure which is now correct and hopefully improved.*

Fig.5D. There should be a dashed line between a11G and a1G according to the data presented here and earlier.

*>> Since we do not discuss XL-MS data pertaining to α11G, we decided to simplify this figure by representing only XL-MS data for α1G.*

Line 155.Fig. 6. Maybe a Table with the start and stop of the different proteins is better? To me the coding of the constructs is confusing, maybe one can use a1G 4-295, a1G 71-295 etc.

*>> we take the reviewer’s point but we refer to our line numbers consistently, and especially in the supplementary files carrying all raw MS data, so we prefer to keep our line numbers in this figure.*

Line 160 the point mutation data can be in supplementary since there are no changes in localization. The whole figure 6 can focus on the deletions since they are the ones giving effects.

*>> we agree (in part) with the reviewer and have now moved all putative lipid binding mutants to a new supplementary figure 2.*

Line 193. How do you know that the larger peaks in a1G is not other proteins in complex with a1G? The other controls does not seem to interact specifically with proteins so that’s why they do not show any higher peaks than a dimer.

*>> the IP data tell us that the next most enriched protein in the alpha1-Giardin co-IP is xaa-dipeptidase, at 16% rIBAQ. The predicted size is ca. 50kDa, thus not in line with the sizes of the detected peaks. Enrichment of larger proteins is several fold lower than enrichment for alpha 1, thus decreasing the likelihood of impact in MP experiments.*

Line 208-222. Remove together with Fig. 7, not giving much to this paper dealing with a1G.

*>> Expressing well described heterologous proteins is a widely used method to investigate various cellular processes. In our study, observing the localization of anxA5 allowed us to favor the hypothesis that giardins can bind membranes even in the absence of an interacting complex and that they are most likely the initiators of this binding. To our knowledge, this is a rare occurrence of a heterologous gene that, when expressed in Giardia, produces a functional protein. This indicates that no extra factors are needed for the correct folding of the annexin, or that they are like the ones present in Giardia. In view of our result, heterologous expression of annexins in Giardia seems a useful tool to study the behavior and properties of these proteins in the absence of their natural partners and cellular context.*

Line 250. How do the different aGs differ in the N terminal? Is a1G and a2G different and does that explain differences in function? Certain aGs are modified in the N terminal, se above, can that modify function-localization?

*>> We take the reviewers’s point but believe this request is beyond scope for this paper and has been addressed in previous publications:*

1. Davids, B. J., Palm, D., Schraner, E. M., Faso, C., & Hehl, A. B. (2011). Identification of alpha-11 giardin, a novel alpha-giardin with Ca2+-dependent phospholipid-binding properties. *Molecular and Biochemical Parasitology*, 179(2), 89–97. [DOI:10.1016/j.molbiopara.2011.06.008]
1. Palm, D., et al. (2003). Identification and characterization of alpha-giardin, a member of a novel multigene family encoding annexin-like proteins in Giardia lamblia. *Molecular and Biochemical Parasitology*, 131(1), 27–33.
2. Feely, D. E., & Conrad, J. T. (2008). Structural characterization of the N-terminal domains of alpha-giardin proteins in *Giardia lamblia*. *Journal of Parasitology Research*, Article ID 482943.
3. Morgan, B. et al. (2013). Functional analysis of the N-terminal motifs of alpha giardin family proteins reveals their role in membrane association. *Parasites & Vectors*, 6:112.

*We included a6G and a11G in our XL-MS analyses (Supplementary data 2) but no intra-links exceeding the molecular interaction distance, as found in a1G, were detected. We also show this in figure 5B. For this reason, we did not embark on a truncation analysis of other aGs.*
Line 282. Tagged and bacterial expressed alpha-4 binds lipids , see above.

*>> The reviewer refers probably to a14G. We have addressed this point in a previous response.*
Line 287-295. What does this mean biologically? Interesting finding but annexin 5 will never end up in Giardia.

*>> It is rare for a non-Giardia protein expressed in Giardia to maintain aspects of its native behaviour. We think this is meaningful because it informs on a certain degree of conservation despite the obvious divergence of our experimental model system.* *Furthermore, and as previously written, this indicates that no extra factors are needed for the correct folding of the annexin, or that they are similar to the ones present in Giardia. In view of our result, heterologous expression of annexins in Giardia seems a useful tool to study the behavior and properties of these proteins in the absence of their natural partners and cellular context.*

Line 308. The modelling can be tested by mutations.

*>> we fully agree with the reviewer, bearing in mind the limitation of working in a wt background, as shown in supplementary figure 2 in the revised manuscript.*

**Editorial requests**

1) Please upload the separate figure files in numerical order.

>> addressed

2) We have amended your Competing Interest statement to comply with journal style. We kindly ask that you double check the statement and let us know if anything is incorrect.

>> this is now correct

3) Please ensure that the Title in your manuscript file and the Title provided in your online submission form are the same.

NOTE: ( N’ terminus / N-terminus ; Giardin / giardin )

>> corrected

4) Please ensure that the affiliations of the authors listed on the manuscript title page (CITY) do exactly match with the affiliations provided in the online submission form

NOTE: Affiliations should include a department (if applicable), an institution, a CITY, and a country

>> addressed

5) We do not publish any copyright or trademark symbols that usually accompany proprietary names, eg ©, ®, or TM (e.g. next to drug or reagent names). Therefore please remove all instances of trademark/copyright symbols throughout the text, including: CultureWell™ on line no 460.

>> removed

6) The file inventory includes files for Figures 4A to 4E, 5A to 5D, 6A to 6C, 7A,7B.. We would recommend either combining these into a single Figure file with separate internal panels, or renumbering them as individual figures , as we are not able to publish multiple components of a single figure as separate files.

NOTE: Please ensure that all included figures are referenced to ensure that your paper is fully reviewed.

>> addressed

7) Thank you for stating "Supplementary Data : All these data can be found by following this link https://eur03.safelinks.protection.outlook.com/?url=https%3A%2F%2Fdrive.google.com%2Fdrive%2Ffolders%2F1gTjcd1uUm9pap4UEjNseQa6UVDkSJznH%3Fusp%3Ddrive_link&data=05%7C02%7Ccarmen.faso%40unibe.ch%7Cd699d84311094183afd808dec5d7551d%7Cd400387a212f43eaac7f77aa12d7977e%7C1%7C0%7C639165725823337064%7CUnknown%7CTWFpbGZsb3d8eyJFbXB0eU1hcGkiOnRydWUsIlYiOiIwLjAuMDAwMCIsIlAiOiJXaW4zMiIsIkFOIjoiTWFpbCIsIldUIjoyfQ%3D%3D%7C0%7C%7C%7C&sdata=vz80Jog%2BW3JfvkqTDy%2B9QPRQuU1rJUqsN9OLyywRpYo%3D&reserved=0

Supplementary videos: All these data can be found by following this link https://eur03.safelinks.protection.outlook.com/?url=https%3A%2F%2Fdrive.google.com%2Fdrive%2Ffolders%2F1gTjcd1uUm9pap4UEjNseQa6UVDkSJznH%3Fusp%3Ddrive_link&data=05%7C02%7Ccarmen.faso%40unibe.ch%7Cd699d84311094183afd808dec5d7551d%7Cd400387a212f43eaac7f77aa12d7977e%7C1%7C0%7C639165725823375563%7CUnknown%7CTWFpbGZsb3d8eyJFbXB0eU1hcGkiOnRydWUsIlYiOiIwLjAuMDAwMCIsIlAiOiJXaW4zMiIsIkFOIjoiTWFpbCIsIldUIjoyfQ%3D%3D%7C0%7C%7C%7C&sdata=%2BqSvc4uYvIIJjyq7BrvGZr2sxpW3XRJsUtFXms0lyt0%3D&reserved=0

Unfortunately, Google drive does not qualify as an acceptable data repository according to PLOS's standards.

At this time, please upload the minimal data set necessary to replicate your study's findings to a stable, public repository (such as figshare or Dryad) and provide us with the relevant URLs, DOIs, or accession numbers that may be used to access these data. For a list of recommended repositories and additional information on PLOS standards for data deposition, please see

https://eur03.safelinks.protection.outlook.com/?url=https%3A%2F%2Fjournals.plos.org%2Fplospathogens%2Fs%2Frecommended-repositories&data=05%7C02%7Ccarmen.faso%40unibe.ch%7Cd699d84311094183afd808dec5d7551d%7Cd400387a212f43eaac7f77aa12d7977e%7C1%7C0%7C639165725823395712%7CUnknown%7CTWFpbGZsb3d8eyJFbXB0eU1hcGkiOnRydWUsIlYiOiIwLjAuMDAwMCIsIlAiOiJXaW4zMiIsIkFOIjoiTWFpbCIsIldUIjoyfQ%3D%3D%7C0%7C%7C%7C&sdata=aRZZocxpVAyvX1dIu9fLh56kLesMxd%2B7ThYdCoUkutw%3D&reserved=0

>> addressed; all supplementary data files and figures are part of the submission. Supplementary videos 1-24 have been uploaded to Drayad and carry the following DOI: https://doi.org/10.5061/dryad.02v6wwqk4

8) Thank you for stating "All proteomics datasets are deposited to the ProteomeXchange Consortium via the PRIDE partner repository. Access coordinates are included in the manuscript."

Please note that your Data Availability Statement is currently missing the DOI/accession number of each dataset OR a direct link to access each database]. If your manuscript is accepted for publication, you will be asked to provide these details on a very short timeline. We therefore suggest that you provide this information now, though we will not hold up the peer review process if you are unable.

NOTE: Please update your Data Availability Statement in the Online Submission Form

>> addressed, the DOI is now part of the revised manuscript and the online submission form. It shall be made public following acceptance.

9) Please amend your detailed Financial Disclosure statement. This is published with the article, therefore should be completed in full sentences and contain the exact wording you wish to be published.

i). State what role the funders took in the study. If the funders had no role in your study, please state: “The funders had no role in study design, data collection and analysis, decision to publish, or preparation of the manuscript.”

ii). If any authors received a salary from any of your funders, please state which authors and which funders.

>> addressed

10) Please ensure that the funders and grant numbers match between the Financial Disclosure field and the Funding Information tab in your submission form. Note that the funders must be provided in the same order in both places as well.

Currently, " The scientific exchanges funding scheme of the Africa-Europe Cluster of Research Excellence-Genomics for Health in Africa (CoREGHA), and further supported by the Swiss Society for Tropical Medicine and Parasitology, and the Graduate School for Cellular and Biomedical Sciences of the University of Bern" are missing from the Funding Information and the grant number "PSTD2204072582" is missing from the financial disclosure.

>>corrected

11) PLOS now requires that authors provide the original uncropped and unadjusted images underlying all blot or gel results reported in a submission’s figures or Supporting Information files. This policy and the journal’s other requirements for blot/gel reporting and figure preparation are described in detail at https://eur03.safelinks.protection.outlook.com/?url=https%3A%2F%2Fjournals.plos.org%2Fplosone%2Fs%2Ffigures%23loc-blot-and-gel-reporting-requirements&data=05%7C02%7Ccarmen.faso%40unibe.ch%7Cd699d84311094183afd808dec5d7551d%7Cd400387a212f43eaac7f77aa12d7977e%7C1%7C0%7C639165725823415680%7CUnknown%7CTWFpbGZsb3d8eyJFbXB0eU1hcGkiOnRydWUsIlYiOiIwLjAuMDAwMCIsIlAiOiJXaW4zMiIsIkFOIjoiTWFpbCIsIldUIjoyfQ%3D%3D%7C0%7C%7C%7C&sdata=56GM2nFQ6cX63xAMLSx8N1GD0pkvmoQS8wNogzpmnx0%3D&reserved=0 and https://eur03.safelinks.protection.outlook.com/?url=https%3A%2F%2Fjournals.plos.org%2Fplosone%2Fs%2Ffigures%23loc-preparing-figures-from-image-files&data=05%7C02%7Ccarmen.faso%40unibe.ch%7Cd699d84311094183afd808dec5d7551d%7Cd400387a212f43eaac7f77aa12d7977e%7C1%7C0%7C639165725823432268%7CUnknown%7CTWFpbGZsb3d8eyJFbXB0eU1hcGkiOnRydWUsIlYiOiIwLjAuMDAwMCIsIlAiOiJXaW4zMiIsIkFOIjoiTWFpbCIsIldUIjoyfQ%3D%3D%7C0%7C%7C%7C&sdata=esQwOE29ys5CuZyWTLzsZZpytd9EuO9CuIH76vVNALI%3D&reserved=0.

When you submit your revised manuscript, please ensure that your figures adhere fully to these guidelines and provide the original underlying images for all blot or gel data reported in your submission. See the following link for instructions on providing the original image data: https://eur03.safelinks.protection.outlook.com/?url=https%3A%2F%2Fjournals.plos.org%2Fplosone%2Fs%2Ffigures%23loc-original-images-for-blots-and-gels&data=05%7C02%7Ccarmen.faso%40unibe.ch%7Cd699d84311094183afd808dec5d7551d%7Cd400387a212f43eaac7f77aa12d7977e%7C1%7C0%7C639165725823445879%7CUnknown%7CTWFpbGZsb3d8eyJFbXB0eU1hcGkiOnRydWUsIlYiOiIwLjAuMDAwMCIsIlAiOiJXaW4zMiIsIkFOIjoiTWFpbCIsIldUIjoyfQ%3D%3D%7C0%7C%7C%7C&sdata=Ne1m1kwTW8%2BVzKZyA0cxdt6il%2B8Tt0QCyPG1c0lnyQ8%3D&reserved=0.

In your cover letter, please note whether your blot/gel image data are in Supporting Information or posted at a public data repository, provide the repository URL if relevant, and provide specific details as to which raw blot/gel images, if any, are not available. Email us at customercare@plos.org if you have any questions.

>>this file is now a new supplementary data 10, and has been uploaded as part of the submission.
