# Supplementary material for: The N-terminus of alpha-1 giardin, a parasitic annexin orthologue, is essential for oligomerization and lipid-binding activity
Source: PLoS Pathog. 2026 Oct 14;22(10):e1014407. doi: 10.1371/journal.ppat.1014407 (PMC13686171; doi:10.1371/journal.ppat.1014407)
Supplement: Peer review history

# Peer review: Decision Letter 0

**DOI:** 10.1371/journal.ppat.1014407.r001

**Authors:** Dominique Soldati-Favre, Pavel Doležal

21 Apr 2026

-->PPATHOGENS-D-26-00541

The N’ terminus of Alpha-1 Giardin, a parasitic annexin orthologue, is essential for oligomerization and lipid-binding activity

PLOS Pathogens

Dear Dr. Faso,

Thank you for submitting your manuscript to PLOS Pathogens. After careful consideration, we feel that it has merit but does not fully meet PLOS Pathogens' publication criteria as it currently stands. Therefore, we invite you to submit a revised version of the manuscript that addresses the points raised during the review process.

Please submit your revised manuscript by Jun 20 2026 11:59PM. If you will need more time than this to complete your revisions, please reply to this message or contact the journal office at plospathogens@plos.org. When you're ready to submit your revision, log on to https://www.editorialmanager.com/ppathogens/ and select the 'Submissions Needing Revision' folder to locate your manuscript file.

Please include the following items when submitting your revised manuscript:

* A letter that responds to each point raised by the editor and reviewer(s). You should upload this letter as a separate file labeled 'Response to Reviewers'. This file does not need to include responses to any formatting updates and technical items listed in the 'Journal Requirements' section below.

* A marked-up copy of your manuscript that highlights changes made to the original version. You should upload this as a separate file labeled 'Revised Manuscript with Track Changes'.

* An unmarked version of your revised paper without tracked changes. You should upload this as a separate file labeled 'Manuscript'.

If you would like to make changes to your financial disclosure, competing interests statement, or data availability statement, please make these updates within the submission form at the time of resubmission. Guidelines for resubmitting your figure files are available below the reviewer comments at the end of this letter.

We look forward to receiving your revised manuscript.

Kind regards,

Pavel Doležal

Guest Editor

PLOS Pathogens

Dominique Soldati-Favre

Section Editor

PLOS Pathogens

Sumita Bhaduri-McIntosh

Editor-in-Chief

PLOS Pathogens

orcid.org/0000-0003-2946-9497

Michael Malim

Editor-in-Chief

PLOS Pathogens

orcid.org/0000-0002-7699-2064

**Additional Editor Comments:**

Dear Carmen,

As you will see your manuscript was seen by three independent reviewers. They all appreaciate submitted work but some improvements and modifications are also recomended. These concern mainly better introduction, integration of the new data with the previous works in the field and also better data presentation.

We are looking forward to receiving your revised manuscript.

Best regards,

Pavel Dolezal

**Journal Requirements:**

1) We ask that a manuscript source file is provided at Revision. Please upload your manuscript file as a .doc, .docx, .rtf or .tex. If you are providing a .tex file, please upload it under the item type u2018LaTeX Source Fileu2019 and leave your .pdf version as the item type u2018Manuscriptu2019.

2) Please provide an Author Summary. This should appear in your manuscript between the Abstract (if applicable) and the Introduction, and should be 150-200 words long. The aim should be to make your findings accessible to a wide audience that includes both scientists and non-scientists. Sample summaries can be found on our website under Submission Guidelines:

https://journals.plos.org/plospathogens/s/submission-guidelines#loc-parts-of-a-submission

3) Please upload all main figures as separate Figure files in .tif or .eps format. For more information about how to convert and format your figure files please see our guidelines:

https://journals.plos.org/plospathogens/s/figures

4) We are unable to open the following Supporting Information file: Supplementary Data 8_plasmid sequences.zip. Please kindly revise as necessary and re-upload.

5) Please amend your detailed Financial Disclosure statement. This is published with the article. It must therefore be completed in full sentences and contain the exact wording you wish to be published.

State what role the funders took in the study. If the funders had no role in your study, please state: "The funders had no role in study design, data collection and analysis, decision to publish, or preparation of the manuscript.".

If you did not receive any funding for this study, please simply state: u201cThe authors received no specific funding for this work.u201d

**Reviewers' Comments:**

Reviewer's Responses to Questions

**Part I - Summary**

Please use this section to discuss strengths/weaknesses of study, novelty/significance, general execution and scholarship.

Reviewer #1: While alpha-giardins were discovered 40 years ago and later identified as adherents and vaccine targets, their biochemistry and structure, which are the focus here, have never been characterized. Here excellent investigators use lipid-binding assays, cross-linking mass spectrometry, site-directed mutagenesis, confocal microscopy, and mass photometry to show the importance of a short N-terminal region of alpha1-giardin for unconventional protein expression. The figures are well-drawn; the methods, results, and discussion are well-written; and mass spec data deposited in PRIDE. The only weakness is the absence in the Introduction of the questions addressed in the paper.

Reviewer #2: This manuscript presents a comprehensive analysis of α-giardins, with a particular focus on α1-giardin and its role in lipid binding, oligomerization, and subcellular localization. The study integrates multiple complementary approaches (confocal microscopy, co-IP, XL-MS, mass photometry, and molecular dynamics), which represents a clear strength.

Although the work is thorough, technically sound, and well presented, with a robust experimental design, it primarily constitutes a further characterization of α1-giardin rather than providing a fundamentally novel conceptual advance (for example, in terms of functional insight).

Reviewer #3: Warmus et al have studied the localization, lipid binding and oligomerization of alpha-giardins (aGs), mainly alpha-1 giardin (a1G). Several aGs localize to peripheral endocytic compartments (PECs) in Giardia, including a1G. The N terminal region of a1G is important for lipidbinding and oligomerization. A model for the role of a1G and other PEC related aGs is suggested. It is a paper with many interesting results but the presentation can be improved. I have the following comments:

**********

**Part II – Major Issues: Key Experiments Required for Acceptance**

Please use this section to detail the key new experiments or modifications of existing experiments that should be absolutely required to validate study conclusions.

Generally, there should be no more than 3 such required experiments or major modifications for a "Major Revision" recommendation. If more than 3 experiments are necessary to validate the study conclusions, then you are encouraged to recommend "Reject".

Reviewer #1: None.

Reviewer #2: 1. Terminology: PVs vs PECs

The term “peripheral endocytic compartments (PECs)” is problematic because it is not widely adopted in the field. Instead, the consensus terminology in recent literature—including primary research articles and reviews from the last five years—is “peripheral vacuoles (PVs)”, reflecting their well-established identity as endo-lysosomal organelles with multifunctional roles.

For example, recent studies consistently describe these structures as PVs and highlight their:

-localization beneath the plasma membrane,

-role in endocytosis, digestion, acidification, and

-functional equivalence to endosomal/lysosomal compartments since it also contains lysosomal proteins (e.g., cathepsins and RNase).

Furthermore, contemporary literature acknowledges the use of dual terminology but still prioritizes PVs. For instance, PVs are described as “peripheral vacuoles/peripheral endocytic compartments (PVs/…)”, explicitly indicating that PVs are the primary accepted term.

Importantly, recent reviews and system-level analyses of Giardia cell biology also consistently use PVs as standard nomenclature when describing trophozoite organization and subcellular compartments.

Taken together, these references support the view that:

PVs are not merely endocytic compartments, but rather hybrid endo-lysosomal organelles with additional MVB-like properties, as widely recognized in the field.

Recommendation: The authors should adopt the term “peripheral vacuoles (PVs)” throughout the manuscript.

2. Variability in expression levels

The manuscript reports variable expression levels for the same giardin constructs (e.g., Fig. 2), but no explanation is provided. This raises several concerns:

• Which vectors/promoters were used for each construct?

• Are these episomal or integrated constructs?

• Could expression variability influence localization or biochemical assays?

Additionally, the rationale for performing immunoprecipitation before lipid strip assays (Fig. 3) should be clarified. Is this due to low expression levels or to enriched complexes?

3. Inconsistent use of constructs

The manuscript states that G25 and G282 are essentially equivalent constructs expressing α1G, differing only minimally (page ~14). However:

• G25 is used in some experiments (e.g., Fig. 6),

• G282 in others (e.g., Fig. 7).

A clear justification is required for this inconsistency. If constructs are interchangeable, consistency should be maintained.

4. Lack of experimental validation of oligomerization

A central claim of the manuscript is the oligomerization of α1G. However:

• This is primarily inferred from mass photometry and XL-MS, not directly demonstrated.

• No biochemical validation is provided.

The authors should strengthen this claim by:

• performing native (non-denaturing) Western blot,

• or complementary biochemical approaches.

Additionally, since G25 is used in several assays, it would be informative to specifically test the oligomerization of this construct.

5. Overinterpretation of oligomerization

The manuscript repeatedly links oligomerization with function and PEC association (e.g., lines 271–272), yet:

• no direct functional assays demonstrate this relationship,

• oligomerization itself is not experimentally validated beyond indirect evidence.

The statement:

“MP and XL-MS data do point towards a major role for oligomerization in functionality and PECs association.”

is therefore an overstatement and should be toned down.

6. Title overstatement

The title claims:

“...is essential for oligomerization and lipid-binding activity”

However:

• oligomerization is not directly demonstrated experimentally,

• its causal relationship with lipid binding is inferred.

The title should be revised to reflect the level of evidence presented.

7. Calcium dependence interpretation

In the Discussion (lines ~281–294), the authors state that α1G lipid binding is not affected by calcium, in contrast to annexin A5.

However, the cited reference (Saroja et al., 2022) indicates:

• α1-giardin disengages from membranes at high calcium concentrations, potentially due to dimer formation.

This appears contradictory. The authors should:

• clarify this discrepancy,

• or revise the interpretation accordingly.

8. Comparison with annexin A5

The conclusion emphasizes similarities between α1G and annexin A5. However:

• the data actually highlight significant differences (e.g., calcium dependence, behavior).

• Therefore, the statement:

“highlighting unique characteristics of α1G... and showing how G. lamblia can be a useful heterologous model...”

is not fully supported and should be reconsidered or softened.

Reviewer #3: (No Response)

**********

**Part III – Minor Issues: Editorial and Data Presentation Modifications**

Please use this section for editorial suggestions as well as relatively minor modifications of existing data that would enhance clarity.

Reviewer #1: none.

Reviewer #2: 9. Insufficient description of expression constructs and vectors

Although vector sequences are provided in FASTA format, it is difficult to evaluate the experimental system. Critical information is missing, including vector backbone, promoters, and selection markers. Either a schematic representation or a detailed description is necessary.

10. Inconsistency in co-IP results

It is unclear why α6-giardin is not recovered in its own top 10 pull-down. This should be explained.

Reviewer #3: Line 51. The Introduction needs serious re-writing and extension. To me it does not look like an Introduction of a paper, it is shorter than the abstract and Giardia and alpha-giardins are not even mentioned! Here it is important to explain for the reader what Giardia is, why is it important to study. What is already known about alpha-giardins and specially a1G when it comes to disease.

Line 60. Remove Fig. 1, it is not needed, annexins can be discussed without a figure.

Line 94. “In Giardia trophozoites”, other cell stages are not studied.

Line 96. Is the gene driven by promoter of each alpha-giardin and is it episomal or integrated constructs? Important info.

Line 101. A C terminal epitope HA-tagging approach is used here to localize the alpha-giardins. Many alpha-giardins have been localized before with epitope tags of different kinds and with specific antibodies. The results in this study is in line with some of that published data but not all.

I suggest to make a table with the localization in this paper and a comparison with all earlier published localizations to get a more clear picture of the localizations.

It should be noted that certain alpha-giardins have C terminal extensions: The sizes of the C-terminal extensions are 17 (α8G), 26 (α14G), and 77 amino acids (α19G).

By using polyclonal serum, α8G was located on the plasma membrane and flagella, not cytoplasm as here. See doi: 10.1016/j.exppara.2010.05.028. ,

a14G binds lipid strips as seen in this paper doi: 10.1128/mSphere.00032-15. and it adheres to microtubules of the flagella via self-assembly that may regulated by Ser/Thr-phosphorylation. doi: 10.1007/s00436-007-0758-6. doi: 10.1016/j.jmb.2008.11.012.

alpha19-giardin, carries also an N-terminal sequence extension of MGCXXS, alpha19-giardin exclusively localizes to the ventral pair of the giardial flagella. A mutant in which the putatively myristoylated N-terminal glycine residue was replaced by alanine lost this specific localization. It has been suggested that the dual lipidation of alpha19-giardin is responsible for its specific flagellar localization. Has membrane association. doi: 10.1128/EC.00136-09.

Thus, the localization in this paper does not fit with earlier data and one should be careful with the cytoplasmic type of localization seen with the alpha-giardins with C terminal extension like a8G and a19G. There is a risk that the HA tag causes mislocalization to the cytoplasm and then the correct modifications and interaction with cellular partners is not seen either, see below.

Line 105 and 106. Use numbering in the right order. Do the same through-out the paper, see also line 118.

Line 113. Was the lipid binding in Fig. 3 dependent on the calcium level? Fig. 3B is not refered to as far as I can see. The proteomics data show that many proteins co-purify with the tagged alpha-giardins. How do you know that it is the tagged alpha- giardin that bind lipids and not the interaction partner?

Line 115+116. This does not fit with earlier data for a8G.

Line 117. Explain what the numbers in paranthesis are.

Line 121. There must be a mistake in Fig. 4B, it is the same data for a6 and a11. Add the orf numbers of the protein, this does not say anything since there are so many variants of some of the proteins.

Fig.5D. There should be a dashed line between a11G and a1G according to the data presented here and earlier.

Line 155.Fig. 6. Maybe a Table with the start and stop of the different proteins is better? To me the coding of the constructs is confusing, maybe one can use a1G 4-295, a1G 71-295 etc.

Line 160 the point mutation data can be in supplementary since there are no changes in localization. The whole figure 6 can focus on the deletions since they are the ones giving effects.

Line 193. How do you know that the larger peaks in a1G is not other proteins in complex with a1G? The other controls does not seem to interact specifically with proteins so that’s why they do not show any higher peaks than a dimer.

Line 208-222. Remove together with Fig. 7, not giving much to this paper dealing with a1G.

Line 250. How do the different aGs differ in the N terminal? Is a1G and a2G different and does that explain differences in function? Certain aGs are modified in the N terminal, se above, can that modify function-localization?

Line 282. Tagged and bacterial expressed alpha-4 binds lipids , see above.

Line 287-295. What does this mean biologically? Interesting finding but annexin 5 will never end up in Giardia.

Line 308. The modelling can be tested by mutations.

**********

PLOS authors have the option to publish the peer review history of their article (what does this mean?). If published, this will include your full peer review and any attached files.

If you choose “no”, your identity will remain anonymous but your review may still be made public.

**Do you want your identity to be public for this peer review?**  For information about this choice, including consent withdrawal, please see our Privacy Policy.

Reviewer #1: **Yes:** John Samuelson

Reviewer #2: No

Reviewer #3: No

[NOTE: If reviewer comments were submitted as an attachment file, they will be attached to this email and accessible via the submission site. Please log into your account, locate the manuscript record, and check for the action link "View Attachments". If this link does not appear, there are no attachment files.]

**Figure resubmission:**

-->While revising your submission, we strongly recommend that you use PLOS’s NAAS tool (https://ngplosjournals.pagemajik.ai/artanalysis) to test your figure files. NAAS can convert your figure files to the TIFF file type and meet basic requirements (such as print size, resolution), or provide you with a report on issues that do not meet our requirements and that NAAS cannot fix.

After uploading your figures to PLOS’s NAAS tool - https://ngplosjournals.pagemajik.ai/artanalysis, NAAS will process the files provided and display the results in the "Uploaded Files" section of the page as the processing is complete. If the uploaded figures meet our requirements (or NAAS is able to fix the files to meet our requirements), the figure will be marked as "fixed" above. If NAAS is unable to fix the files, a red "failed" label will appear above. When NAAS has confirmed that the figure files meet our requirements, please download the file via the download option, and include these NAAS processed figure files when submitting your revised manuscript.

**Reproducibility:**

To enhance the reproducibility of your results, we recommend that authors of applicable studies deposit laboratory protocols in protocols.io, where a protocol can be assigned its own identifier (DOI) such that it can be cited independently in the future. Additionally, PLOS ONE offers an option to publish peer-reviewed clinical study protocols. Read more information on sharing protocols at https://plos.org/protocols?utm_medium=editorial-email&utm_source=authorletters&utm_campaign=protocols

# Author comment: Author response to Decision Letter 1

**DOI:** 10.1371/journal.ppat.1014407.r002

11 Jun 2026

Attachment Submitted filename: Warmus et al_Response to Reviewers.docx

# Editor report: Decision Letter 1

**DOI:** 10.1371/journal.ppat.1014407.r003

**Authors:** Dominique Soldati-Favre, Pavel Doležal, Dominique Soldati-Favre, Pavel Doležal

23 Jun 2026

Dear Prof. Dr Faso,

We are pleased to inform you that your manuscript 'The N-terminus of alpha-1 giardin, a parasitic annexin orthologue, is essential for oligomerization and lipid-binding activity' has been provisionally accepted for publication in PLOS Pathogens.

Before your manuscript can be formally accepted you will need to complete some formatting changes, which you will receive in a follow up email. A member of our team will be in touch with a set of requests.

Please note that your manuscript will not be scheduled for publication until you have made the required changes, so a swift response is appreciated.

IMPORTANT: The editorial review process is now complete. PLOS will only permit corrections to spelling, formatting or significant scientific errors from this point onwards. Requests for major changes, or any which affect the scientific understanding of your work, will cause delays to the publication date of your manuscript.

Should you, your institution's press office or the journal office choose to press release your paper, you will automatically be opted out of early publication. We ask that you notify us now if you or your institution is planning to press release the article. All press must be co-ordinated with PLOS.

Thank you again for supporting Open Access publishing; we are looking forward to publishing your work in PLOS Pathogens.

Best regards,

Pavel Doležal

Guest Editor

PLOS Pathogens

Dominique Soldati-Favre

Section Editor

PLOS Pathogens

Sumita Bhaduri-McIntosh

Editor-in-Chief

PLOS Pathogens

orcid.org/0000-0003-2946-9497

Michael Malim

Editor-in-Chief

PLOS Pathogens

orcid.org/0000-0002-7699-2064

***********************************************************

Dear Dr. Faso,

Thank you for submitting the revised version of your manuscript, "The N’ terminus of Alpha-1 Giardin, a parasitic annexin orthologue, is essential for oligomerization and lipid-binding activity."

We have carefully considered the revised manuscript, the reviewers' comments, and your detailed responses. The revision has improved the manuscript and the authors have addressed the reviewers concerns including revisions to figures and supplementary material, and appropriate modifications to the text.

I am therefore pleased to inform you that your manuscript is accepted for publication in PLOS Pathogens, pending any final editorial and production requirements.

Congratulations to you and your co-authors:-).

Sincerely,

Pavel Doležal

Guest Editor

PLOS Pathogens

Reviewer Comments (if any, and for reference):

# Editor report: Acceptance letter

**DOI:** 10.1371/journal.ppat.1014407.r004

**Authors:** Dominique Soldati-Favre, Pavel Doležal, Dominique Soldati-Favre, Pavel Doležal

Dear Prof. Dr Faso,

We are delighted to inform you that your manuscript, "The N-terminus of alpha-1 giardin, a parasitic annexin orthologue, is essential for oligomerization and lipid-binding activity," has been formally accepted for publication in PLOS Pathogens.

We have now passed your article onto the PLOS Production Department who will complete the rest of the pre-publication process. All authors will receive a confirmation email upon publication.

The corresponding author will soon be receiving a typeset proof for review, to ensure errors have not been introduced during production. Please review the PDF proof of your manuscript carefully, as this is the last chance to correct any scientific or type-setting errors. Please note that major changes, or those which affect the scientific understanding of the work, will likely cause delays to the publication date of your manuscript. Note: Proofs for Front Matter articles (Pearls, Reviews, Opinions, etc...) are generated on a different schedule and may not be made available as quickly.

Soon after your final files are uploaded, the early version of your manuscript, if you opted to have an early version of your article, will be published online. The date of the early version will be your article's publication date. The final article will be published to the same URL, and all versions of the paper will be accessible to readers.

For Research Articles, you will receive an invoice from PLOS for your publication fee after your manuscript has reached the completed accept phase. If you receive an email requesting payment before acceptance or for any other service, this may be a phishing scheme. Learn how to identify phishing emails and protect your accounts at https://explore.plos.org/phishing.

Thank you again for supporting open-access publishing; we are looking forward to publishing your work in PLOS Pathogens.

Best regards,

Sumita Bhaduri-McIntosh

Editor-in-Chief

PLOS Pathogens

orcid.org/0000-0003-2946-9497

Michael Malim

Editor-in-Chief

PLOS Pathogens

orcid.org/0000-0002-7699-2064
